# The Contribution of Preschool Meals to the Diet of Finnish Preschoolers

**DOI:** 10.3390/nu11071531

**Published:** 2019-07-05

**Authors:** Liisa Korkalo, Kaija Nissinen, Essi Skaffari, Henna Vepsäläinen, Reetta Lehto, Riikka Kaukonen, Leena Koivusilta, Nina Sajaniemi, Eva Roos, Maijaliisa Erkkola

**Affiliations:** 1 Department of Food and Nutrition, University of Helsinki, P.O. Box 66, 00014 Helsinki, Finland; 2 Seinäjoki University of Applied Sciences, Kampusranta 11, 60101 Seinäjoki, Finland; 3 Folkhälsan Research Center, Topeliuksenkatu 20, 00250 Helsinki, Finland; 4 Department of Social Research, Faculty of Social Sciences, Assistentinkatu 7, University of Turku, 20014 Turku, Finland; 5 Faculty of Educational Sciences, P.O. Box 8, University of Helsinki, 00014 Helsinki, Finland; 6 School of Applied Educational Sciences and Teacher Education, Philosophical Faculty, University of Eastern Finland, P.O. Box 111, 80101 Joensuu, Finland; 7 Department of Public Health, Clinicum, University of Helsinki, P.O. Box 20, 00014 Helsinki, Finland

**Keywords:** preschool-aged children, kindergarten, day care center, catering, food consumption, dietary intake

## Abstract

Preschool meals may influence the formation of children’s dietary habits and health. We assessed the contribution of preschool meals to the diet of Finnish children. We used food record data from the cross-sectional DAGIS survey and selected recording days which included all three meals (breakfast, lunch, afternoon snack) at preschool. We analyzed the diet of three- to four-year-olds (*n* = 324) and five- to six-year-olds (*n* = 233). Preschool meals accounted for 54% of the weekday’s energy intake in both age groups, and provided ≥60% of total fiber, polyunsaturated fatty acids, and vitamins D and E. More than 60% of fish dishes but only one third of total daily fresh fruit were consumed at preschool. The mean (SD) percentages of energy from protein and fat at preschool were 17% (3%) and 30% (7%) in the younger and 17% (3%) and 31% (6%) in the older age group, respectively. The mean proportions of energy from added sugar at preschool were below 5% in both age groups. On average, salt intake exceeded recommendations and 60% of salt came from preschool food. Tackling high salt intake should be a future goal of guidance for early childhood education and care food services.

## 1. Introduction

Food behaviors are learnt during childhood and track into adulthood [1,2]. Dietary habits influence health over the long term [3,4] and for this reason, ensuring health-promoting food habits both at home and in early childhood education and care (ECEC) is of vital importance. Studies reporting the food consumption and/or nutrient intake of the same children both at home and in ECEC are limited [5,6,7,8,9] and only a few have been conducted in Europe [6,8]. A study in Finland [10] found that the diet of three-year-old children attending ECEC outside the home was closer to the Finnish nutrition recommendations than the diet of home-cared children. Studies conducted in the USA have reported that lunches at ECEC are, regarding some micronutrients, more nutrient-dense than lunches at home or away [11] or dinners at home [9]. In contrast, Gubbels et al. [8] reported that more vegetables were consumed at home than in day care in the Netherlands. The same study found that sweet snacks were mostly eaten in day care. In the study by Gubbels et al. [8], all food in the participating day care centers was provided by the center (Jessica Gubbels, personal communication 9 May 2019). The results regarding the contribution of ECEC to food consumption and nutrient intake are contradictory. The discrepancies may be due to differences in food culture and food service systems, regulations, recommendations, and laws, which need to be taken into account when comparing studies.

The aim of this study was to assess the contribution of preschool meals to the diet of children who attend full-time care in municipal preschools in Finland. We specifically aimed to calculate what is the percentage contribution of each meal to the total daily intake of energy and nutrients on weekdays when the child eats three meals at preschool and to describe the amounts of foods consumed and the sources of nutrients at preschool and at outside preschool. In this paper, we use the term preschool to mean municipally arranged center-based ECEC, which in Finland is voluntary until the age of six, and compulsory for one year before school starts at the age of seven.

## 2. Materials and Methods

### 2.1. Early Childhood Education and Care and Food Services in Finland

In Finland, parents can choose municipal (public) or private ECEC. In addition to preschools, children can attend to group family day care, or family day care, which is typically in the caregiver’s home. Of one- to six-year old Finnish children in ECEC, 76% are in preschools [12]. The ECEC fee is moderate but depends on family’s size and income level. Children from all socio-economic backgrounds are entitled to high-quality ECEC throughout the country. Early education is based on the Act on Early Childhood Education and Care [13] and the National Core Curriculum for Early Childhood Education and Care [14].

According to Finnish law, municipal preschools must provide healthy food which fulfils the child’s nutritional requirements [13]. If a child is in full-time care, three meals (breakfast, lunch, and afternoon snack) must be offered. A target has been set that these three meals should cover two thirds of a child’s daily energy intake [15]. Food for preschool is provided by either the municipality’s own food service or an external food service provider. Food recommendations with meal-specific nutritional criteria are available to guide ECEC food services. The recent updates to children’s food recommendations [16] and the first national food recommendations for ECEC [15] were not yet in effect at the time of the data collection for this study, but older nutrition recommendations for families with children were valid [17].

Lunch generally consists of typical Finnish foods: a warm main course with salad or a main course soup; bread and spreadable fat; and a drink (milk, sour milk, or non-dairy milk substitute suitable for special diets). Breakfast typically consists of porridge with milk and/or berry soup, fruit puree, or jam; and/or bread, spreadable fat and a cold cut; and possibly a piece of fruit or vegetable. A typical afternoon snack is a combination of two or more of the following: bread, yogurt, Finnish cultured milk (*’viili’*), quark (*’rahka’*, also a cultured milk product), smoothie, berry soup, flavored porridge, pancakes, a piece of fruit or vegetable, a cold cut, and milk or juice. All meals are included in the client fee and no separate fees can be charged. This catering is part of the national effort to establish good nutrition, health, and welfare for children. According to the guidelines [15], mealtimes are part of early childhood education. They must be appropriately organized and supervised. The health-related and social role of meals, the objectives of nutritional education and learning manners and food culture, as well as the recreational aspect of eating occasions should be taken into account when arranging mealtimes. Meals are used as a pedagogical tool. The entire educational community should have commonly determined objectives and implementation policies for food education [15].

### 2.2. Study Participants

**Figure 1:**
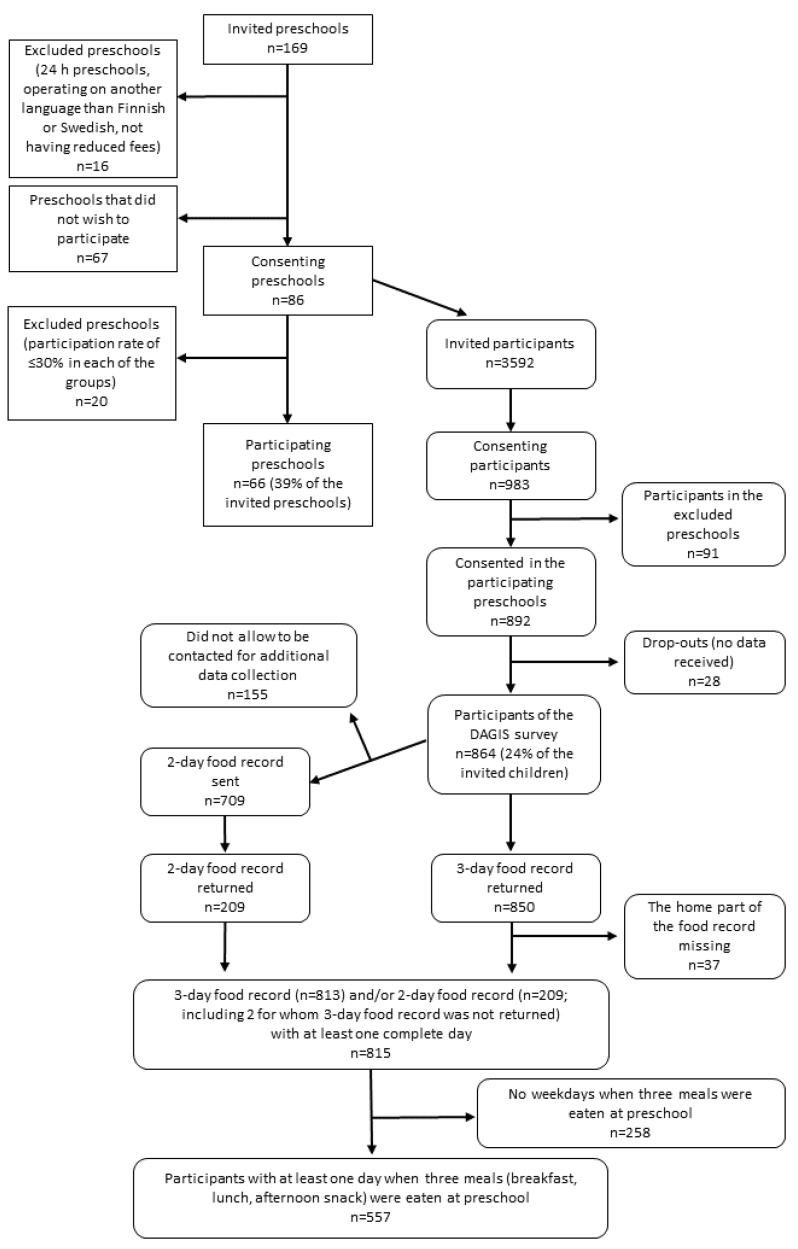
Participation of preschools and children in the DAGIS survey and the selection of participants for this paper.

We used data from the Increased Health and Wellbeing in Preschools (DAGIS) research project. As a part of the larger DAGIS study, a cross-sectional survey of preschool children was conducted in 2015–2016. Details of the sampling process are described in open access format elsewhere [18]. In short, the cross-sectional survey was conducted in eight municipalities. Five of these were in Southern Finland and three were in Western Finland. Altogether 86 municipal (public) preschools consented to participate (Figure 1). From these preschools, all children in the target age of three to six years (*n* = 3592) and their families were invited to participate through an invitation letter. Children in preschools with a low participation rate (≤30% in each of the preschool groups for three- to six-year-olds) were excluded. The final sample consisted of 864 children (24% of those invited) from 66 preschools. These preschools operated from Monday to Friday. We excluded preschools operating 24 h a day from the sample.

A parent or legal guardian of each participating child provided written informed consent. We asked each family if we could contact them again for additional data collection. All procedures involving human subjects were approved by the University of Helsinki Ethical Review Board in the Humanities and Social and Behavioral Sciences on 24 February 2015 (Statement 6/2015).

### 2.3. Anthropometric and Background Data

Trained researchers measured weight and height at the preschool. The children removed their shoes and heavy clothing. The clothes that the child was wearing during the weight measurement were recorded and later deducted accordingly, creating a corrected weight variable. Body weight was measured to the nearest 0.01 kg using CAS portable bench scales (CAS PB-100/200). Height was measured to the nearest 0.1 cm using stadiometers (SECA 217). We used the extended international body mass index cut-offs for thinness and overweight [19]. Other background data such as the parents’ level of education and the child’s special diets were gathered via questionnaires.

### 2.4. Food Record Data

We sent each participating family a three-day food record including instructions. Exact dates (two weekdays and one weekend day) for filling in the food record were assigned for each family. As the aim was that all the days of the week would be well-represented in the data, these three days were not always consecutive. In some cases, when the parents felt that the dates were unsuitable for keeping a food record (for example due to illness in the family or a holiday trip), they contacted the study group and renegotiated the dates. The three-day food records were kept between September 2015 and April 2016.

To capture seasonal variation in the diet, after about six months, the families who had agreed to be contacted for additional data collection were sent an invitation to fill in a second food record (*n* = 709). This time it was a two-day food record and the families were assigned a week during which they should choose two days for recording (with preferably at least one day being a weekday). When necessary, parents also took the record and instructions to preschool. The two-day food records were kept between June 2016 and September 2016. The time between the two food records ranged from 4 to 11 months.

The instruction page of the food records advised parents to record all foods and beverages that their child consumed during the recording days, except for what they consumed at preschool. An example page was also included. We provided the families with a validated [20] Children’s Food Picture Book [21], specifically designed for use in this project to assist in portion size estimation. The parents were instructed to estimate the portion sizes eaten using the picture book, weighing, household measures such as teaspoons or tablespoons, or package labels. The instruction was to list all the ingredients of composite dishes. For packed food products, the exact brand and product name was required. They were also asked to record the place and time of consumption.

The preschool personnel were given a separate pre-coded food record for recording food consumption at the preschool on the dates matching the home food record. The researchers/research assistants instructed the early educators orally, and the food record included written instructions. Breakfast, lunch, afternoon snack, and possible additional snacks each had predefined sections. Different food groups, such as main courses, side dishes (potatoes, pasta, rice), and salad at lunch each had predetermined rows. The early educators were given the Children’s Food Picture Book [21] to help them record the portion sizes eaten. They could also estimate the amounts in household measures.

The research assistants checked the returned food records and, if necessary, made follow-up phone calls to complete missing details of foods consumed. Special attention was paid to vegetable, fruit, and sugary product consumption in the food record checking process. As an example, if the parent had forgotten to record the type of yogurt product, we asked if it had been natural yogurt or sugar-sweetened yogurt; or if the portion size was missing, we asked for more details.

The food data were recorded using AivoDiet dietary software. This software included the Fineli Food Composition Database Release 16 (2013) of the National Institute for Health and Welfare. New food items were also added to the database when necessary. We checked that the vitamin D values of foods fortified with vitamin D (fluid milk products, spreadable fats, and non-dairy milk substitutes) corresponded with the products on the market at the time of study. The database includes recipes for typical Finnish mixed dishes. For each individual meal, the research assistant used a suitable recipe from the database, modified an existing recipe, or created a new recipe according to the parents’ reports. The salt content of home dishes was also based on the recipes in the database and unless the parents stated otherwise in the record, main dishes, porridges, rice, pasta, and potatoes were assumed to have been cooked using salt. We asked the preschool food services if they were willing to give their recipes to the study group to enable more precise calculation of the children’s dietary intake at preschool. Out of the eight municipalities, five gave their recipes, one gave a part of their recipes and two municipalities declined the request. In cases in which the recipe was not available, we made estimations based on the recipes used in the other municipalities.

During the data entry, the research assistants coded each meal with a tag that specified the name of the meal and the place at which it was eaten. These nametags were: breakfast, lunch, dinner, snack, evening snack, and other. The research assistants decided on these based on the recorded time of day, the content of the meal, and general knowledge of Finnish food habits. The place tags were home, preschool, restaurant, and other.

After the data were entered, we checked for outlying values of food consumption in grams and outlying energy and nutrient intakes. After extracting the data from the software, each food code (food item or mixed dish) appearing in the data set was assigned to a food group and nutrient retention factors [22] were applied using a single factor per nutrient per food group. The food composition database did not include values for added sugar. As previously described [23], we estimated added sugar intake by first assigning each food item to a food group and then giving each food group that contained significant amounts of sugar a formula that represented the foods in that group. To estimate the relative amounts of naturally occurring and added sugar in a certain food, we used the information from package labels, the national food composition database and commonly used recipes.

The home and/or preschool food records of 850 (98% of the DAGIS survey sample) and 206 (29% of those invited) children were returned in the first and second round of food record data collection, respectively. However, the home part of the 3-day food record was missing for 37 children. Individual days of data were also excluded due to unrealistically long pauses between consecutive meals (>8 h). After data checking and entry, 815 children (94% of the DAGIS survey sample) had at least one day of food record data available for analysis.

### 2.5. Data Processing and Analysis

To assess the dietary contribution of preschool meals among children attending full-time care, we defined a ‘full preschool day’ and selected a sub-sample of the participants as follows. We selected each singular food recording day when a child had eaten all three preschool meals (breakfast, lunch, and afternoon snack). We defined this as >0 grams of consumption of any food or beverage during all these three meals. Using this criterion, we, in effect, discarded data for all weekend days and those weekdays when: (1) the child was home-cared (it is common in Finland for a child to only attend preschool four days per week) (2) the child came to preschool after preschool breakfast serving time (3) the child was away from preschool for the whole day or part of the day for other reasons, such as being ill or on vacation. The three meals were provided daily in all the participating preschools. Sometimes, when an additional snack was served during excursions or special events, we collapsed these with the regular afternoon snack in the data analysis. This approach yielded a sample of 557 children (64% of the DAGIS survey sample). Each of these children contributed to the data with one to four full preschool days; the total number of days in the data for this paper was 966. These children were from 489 families (423 families had one child in the sample, 64 had two, and 2 had three children in the sample). For analysis, we divided the data into two age groups. We used age at recruitment for this categorization (even though the children grew older between the first and second food recording period).

We rearranged all the meals in the data to fall under one of the seven possible meal categories (1) breakfast outside preschool, (2) breakfast at preschool, (3) lunch at preschool, (4) afternoon snack at preschool, (5) dinner outside preschool, (6) evening snack outside preschool, and (7) other snack outside preschool. We did not consider a glass of water or a chewing gum alone to be a meal and collapsed these with another meal. We first calculated the percentage of individual days in the data that included each meal. After that, we calculated an average day for each child and used these to calculate the mean intakes and population proportions [24] of energy and nutrients during each meal, during the preschool day in total, and during the whole day. We also calculated the mean amounts of foods consumed and the food group sources of energy and nutrients. This was also done after calculating an average day for each child. Finally, since not all the days included all the seven meals, we calculated the mean intake of energy and nutrients for each meal during the days when the meal was consumed after selecting these days and calculating an average day for each child. We used R version 3.5.2 for all analyses.

## 3. Results

The analyses included 557 children from 66 preschools, 264 (47%) of whom were girls. The majority (98%) of these children were in the target age group of three to six years. However, nine children were two years old and one was seven years old; for analysis, we included them in the closest age group. Of all the participants, 81% were categorized as being of normal weight. The participating children attended preschool on four and a half days per week on average (Table 1).

### 3.1. Food Consumption

We found that the majority (≥60%) of skimmed milk, potatoes and potato dishes, fish dishes, poultry dishes, sausage dishes, margarine and fat spread (In this paper, we use the European Union’s definitions [25] for the following spreadable fats: butter, margarine, fat spread, and blended spread. In short, fat spreads and margarines are similar to each other, but the total fat content differs. Blended spreads are obtained from a mixture of vegetable and animal fats and the milk-fat content is between 10% and 80%.), porridge, fruit and berry soups, dairy-based desserts, rye crispbread, and multi-grain bread were consumed at preschool (Table 2 and Table 3). The majority (≥60%) of fresh fruit, berries, sweet and savory bakery products, biscuits and muesli bars, blended spread, yogurt and Finnish cultured milk, and cheese were consumed at home (and elsewhere outside preschool). Furthermore, over 75% of the total amount of the ‘sweets and sugar’ group and sugar-sweetened juice and all soda were consumed outside preschool. Fresh vegetables and vegetable salads were consumed in roughly equal amounts at preschool and outside preschool.

At preschool, skimmed milk was the most commonly used milk. None of the preschools offered whole milk. Of the 66 preschools, 64 (97%) offered milk with and 2 (3%) without vitamin D fortification during the food recording days. The mean daily bread consumption was 65 g and 71 g among the three- to four-year-olds and five- to six-year-olds, respectively, and about two thirds of the bread consumption was at preschool (Table 2 and Table 3). Preschools offered margarine or fat spread with bread and their consumption was relatively high (mean 16 and 18 g/day, among the three- to four-year-olds and five- to six-year-olds, respectively), which is in line with their bread consumption pattern. The most common spreadable fat offered at preschool was margarine with a fat content of 60 g/100 g (data not shown).

### 3.2. Energy and Macronutrients

The mean energy intake for Monday to Friday was 5.6 MJ among the three- to four-year-olds (Table 4) and 6.4 MJ among the five- to six-year-olds (Table 5). All preschool meals together accounted for 54% of the total energy intake in both age groups. Lunch and dinner were the two main meals regarding energy intake. Breakfast at preschool, afternoon snack at preschool, and evening snack at home were all of similar importance regarding energy intake. Most children did not have a home breakfast before going to preschool, and thus, on average, home breakfast was only 2–3% of the energy intake (Table 4 and Table 5). Among those who did have a home breakfast, it contributed on average 0.50–0.57 MJ of energy (Supplementary Tables S1 and S2).

The mean proportions of energy from protein and saturated fatty acids in preschool meals were above those recommended for ECEC food services (Table 6). The different food groups are presented as sources of energy and nutrients in Supplementary Tables S3–S58. The main sources of energy were cereals and bakery products, milk and dairy products, and meat and meat dishes in both age groups, both at and outside preschool (Supplementary Tables S3 and S4). Lunch and dinner accounted for most of the protein and fat in the children’s diet (Table 4 and Table 5). Milk and dairy products, meat and meat dishes, and cereals and bakery products were the greatest sources of protein both at home and at preschool (Supplementary Tables S5 and S6). The ‘margarine and fat spread’ sub-group was the most important source of mono- and polyunsaturated fatty acids, but also of saturated fatty acids during preschool hours (Supplementary Tables S17–S22). The proportion of saturated fat from milk and dairy products was lower at preschool than outside preschool (Supplementary Tables S17 and S18). On average, dinner and lunch were the meals that contributed the highest amounts of saturated fatty acids.

The mean daily intake of fiber was 15.3 g and 16.7 g among the three- to four-year-olds and five- to six-year-olds, respectively. Of this, more than a third was contributed by cereals and bakery products consumed at preschool (Supplementary Tables S13 and S14). The mean percentage of energy from added sugar on Monday to Friday was 6.2 E% in the younger and 7.0 E% in the older age group (Table 6). Most of the sucrose and added sugar intake was outside preschool (Table 4 and Table 5). The meals accounting for the most added sugar intake on weekdays were the afternoon snack at preschool, evening snack, and other snacks outside preschool. The main source of added sugar at preschool was fruit and berry soups (Supplementary Tables S11 and S12), which were eaten at the afternoon snack and breakfast. However, the mean percentage of energy from added sugar at preschool was only 4–5 E% (Table 6).

### 3.3. Micronutrients

Preschool food was an important source of vitamins D and E (≥60% of total intake in both age groups; Table 4 and Table 5). Vitamin D fortified milk and margarine and fat spread were the main sources of vitamin D (Supplementary Tables S25 and S26). The sub-group ‘margarine and fat spread’ was the main source of vitamins A and E in preschool (Supplementary Tables S23, S24, S27, and S28). Calcium intake was distributed evenly between the five main meals of the day (Table 4 and Table 5) and milk was its main source (Supplementary Tables S49 and S50). Milk was also the most important source of riboflavin (Supplementary Tables S31 and S32) and iodine (Supplementary Tables S57 and S58). Folate came from a large variety of food groups in the diet, but at preschool, breads other than white bread were important sources (Supplementary Tables S37 and S38). Fresh fruit was a more important source of vitamin C at home than at preschool (Supplementary Tables S41 and S42). Preschool food contributed 60% to the total salt intake in both age groups; the mean salt intake at preschool was 3.2 and 3.6 g/day in the younger and older age group, respectively (Table 4 and Table 5). Meat and meat dishes, and cereals and bakery products were especially significant sources of salt (Supplementary Tables S43 and S44).

## 4. Discussion

We investigated the contribution of preschool meals to the weekdays’ total energy and nutrient intake among Finnish preschoolers who eat three meals at preschool. We also examined the amounts of the foods consumed and the food sources of nutrients during preschool and outside preschool hours. We found that preschool meals contributed significant shares of some of the food groups that are considered part of a health-promoting diet [16] (fish, fat-free dairy, whole-grain products, and vegetable-oil-based spreadable fats). Preschool meals were also low in sugary foods. Preschool food provided on average 54% of the total energy on a weekday, and ≥60% of the total fiber, polyunsaturated fatty acids, and vitamins D and E. Salt intake was too high overall, and 60% of salt came from preschool food.

The results show that the most typical meal pattern of a Finnish preschool child consists of five meals per day: breakfast, lunch, afternoon snack, dinner, and evening snack. Small snacking also occurred outside these meals. Although lunch and dinner were the main meals, more than half of the total daily energy was consumed during the other meals. In a study of Dutch children, lunch and dinner together had a higher share of total energy than that in our study and other meals accounted for about 44% of the daily energy [8]. In our study, each of the three smaller meals (breakfast at preschool, afternoon snack at preschool and evening snack outside preschool) provided a similar share of energy. Common anecdotes on the importance of breakfast should not obscure the fact that based on energy intake, afternoon and evening snacks are as important as breakfast in the diet of Finnish preschoolers. Thus, breakfast, afternoon snack, and evening snack deserve equal attention regarding healthy food choices.

In our study, the total daily energy intake on weekdays for three- to four-year-olds was 5.6 MJ, which is comparable with the results of Goldbohm et al. [26] in the Netherlands, in which the mean daily total energy intake for three-year-old children attending day care was 5.8 MJ. We found that preschool meals did not cover two thirds of the daily energy, which is the target level set in the Finnish recommendation for ECEC food services [15]. The distribution of energy between day care and home among three-year-old children in the Netherlands was similar to that in our findings; their energy intake in day care was about 3.0 MJ/d and the daily total 5.9 MJ [8].

The distribution of the consumption of fish between home and ECEC has seldom been reported. Lehtisalo et al. [10] found more consumers of fish among Finnish children who attended ECEC than among children taken care of at home. In our study, the consumption of fish dishes at preschool was about twice that consumed at home. Our results concerning fresh fruit consumption contradict those of previous studies in the Netherlands [8] and Oklahoma, USA [9], which found higher consumption in ECEC than at home. Overall, there is room to increase fruit intake at Finnish preschools.

Our study supports previous findings that more low-fat dairy products [9,10] and more vegetable oil-based spreadable fats [10] are consumed in ECEC than at home. Despite this positive finding, the mean proportion of energy from saturated fatty acids exceeded the recommendation for ECEC food services (<10 E%). Meat and meat dishes, along with spreadable fats, were important sources of saturated fatty acids. An increased amount of vegetarian main courses, if well accepted by the children and if prepared without ingredients high in saturated fatty acids such as cheese, might be one strategy to improve the fatty acid profile as well as to increase vegetable consumption at preschools. However, it should be noted that about half of the saturated fatty acid intake (but less than half of the energy intake) occurred outside preschool.

Bread consumption (largely as whole-grain bread) and along with it, also margarine/fat spread consumption, were relatively high at preschool. This combination provided large shares of nutrients such as fiber; folate; unsaturated fatty acids; and vitamins A, D, and E. On the other hand, bread with margarine/fat spread also contributed significantly to the saturated fatty acid and salt intakes, both of which should be reduced. Margarines with 60% fat content generally contain 16–18 g of saturated fatty acids/100 g [27] and the development of products with an even lower saturated fatty acid content could be useful in reducing the saturated fatty acid intake. Since flour is not fortified in Finland, white bread was not a relevant source of total folate (including folic acid).

We found that protein intake was higher at preschool than recommended, and milk and dairy products and meat and meat dishes were important sources of protein. Finnish food-based dietary guidelines state that over the day, 4 dL of fluid milk products (e.g., milk, sourmilk, yogurt, Finnish cultured milk) and one slice of cheese is enough for preschool-aged children [16]. Our results show that on average, this is exceeded on weekdays. It might be possible to adjust the protein content and type (animal vs. plant protein) of preschool meals by reducing the amount of milk and adding more vegetarian dishes to the menu.

In our study, the percentage of energy from added sugar at preschool was well within the limits of the recommendation (<10 E%) [15]. Strategies to limit the use of added sugar in the food services at the participating preschools included, for example, sweetening plain yogurt with only fruit and berry purees. The main sources of added sugar outside preschool were sugar-sweetened dairy products, beverages, bakery products, and sweets and chocolate. At preschool, a considerably smaller amount of added sugar came from these food groups. In contrast to our results, some previous studies have found that sweet snacks are more often consumed in ECEC than at home [8,11]. When interpreting the results of our study, it is important to note that we did not include weekend days, which are likely to include more sugary products, in the analysis. We found that the mean density of fiber was 3 g/MJ at preschool in both age groups. In the Netherlands, three-year-olds on average ate a total of 13 g of fiber per day, of which 7.2 g was at day care [8], which is slightly lower than in our sample.

The total daily salt intake of two- to ten-year-old children should not exceed 3 to 4 g/day [16] and the intake at preschool should not exceed 2 to 2.6 g/day [15]. We found that the mean salt intake on weekdays far exceeded these recommendations. In Belgium, the total sodium intake of preschool children also greatly exceeded the acceptable range [28]. In Portugal, children eating preschool meals had a total intake of salt of about 6.3 g/day [29]. In our study, cereals and bakery products were the main source of salt at preschool. Meat and meat dishes were also an important source of salt both at and outside preschool. Preschool food services should reduce the sodium content in their porridge and main course recipes, and offer low-sodium bread.

A limitation of the study was the low participation rate in the DAGIS cross-sectional survey (24%) and the fact that the strict inclusion criteria further limited the sample used in the analyses for this paper. The data also includes siblings, meaning that when means were calculated, some families had a larger representation than others. The estimate of salt intake at home may be more prone to bias than that at preschool due to the fact that in the home dishes, salt content estimates were based on typical Finnish recipes in the database, whereas at preschool, the salt content of the dishes was, for most municipalities, based on recipes provided by the food services. A strength of this study was its comparatively large sample. Its methodological strengths included the use of a picture book that was specifically designed for use in children’s portion size estimation. This has been used with similar accuracy by parents and ECEC personnel in a validity study [20]. Detailed information on the recipes and products used at preschools further aided the careful analysis of the diet.

Meal planning in ECEC food services may have an important influence on the formation of dietary habits and long-term effects on the health of children. Only a few previous studies have examined how different meals at ECEC and at home contribute to the diets of children. Although our study was a single-time cross-sectional study, the results are useful for the purposes of nutritional education, communication, and policy target setting for further developing the dietary quality of preschool meals. A long-term monitoring system of the actual dietary intake at preschools in Finland would be even more effective. As Lucas et al. [30] point out, such monitoring would allow the evaluation of how changes in policy and guidance actually affect children’s intakes. As an example, possible changes in milk consumption should be accompanied by an analysis of their effects on the overall diet, as milk is an important source of a variety of nutrients in Finnish children’s diets.

## 5. Conclusions

Finland provides preschool meals free of charge and the law requires the served food to be healthy. We found that Finnish children who attend full-time care eat preschool meals that contribute significant shares of favorable food groups and nutrients to their total weekday diet. Salt intake, however, was high at preschool and exceeded the recommendation. Thus, tackling salt intake is an important goal for guidance. In addition, fruit consumption and vegetarian food consumption at preschool could be increased. The intake of protein and saturated fatty acids also needs to be monitored.

## Figures and Tables

**Table 1:** Characteristics of sample (total *n* = 557).

Characteristics	3- to 4-Year-Olds (*n* = 324)		5- to 6-Year-Olds (*n* = 233)	
	Data Available, *n*	% or Mean (SD)	Data Available, *n*	% or Mean (SD)
**Child**				
Gender	324		233	
Girls		47.2		47.6
Boys		52.8		52.4
Age, years	324	4.1 (0.6)	233	5.6 (0.5)
				
Weight status [19]	301		224	
Underweight		8.0		5.8
Normal weight		83.1		78.6
Overweight or obese		9.0		15.7
				
Days in preschool/week	312	4.5 (0.8)	219	4.5 (0.8)
Hours in preschool/day	305	8.2 (0.8)	211	8.1 (0.8)
				
Special diets	322		226	
Food allergy or intolerance		8.4		7.5
Low lactose/lactose free		7.6		5.7
Gluten-free		0.9		1.3
Vegetarian or pescovegetarian		0.3		0.4
				
**Family**				
Highest educational level in family	315		221	
High school level or lower		19.1		18.0
Bachelor’s degree or equivalent		43.5		42.1
Master’s degree or higher		37.3		39.9
				
Number of < 18-year-old children living in the household, including participating child	315		221	
1		11.7		11.8
2		58.7		55.7
3 or more		29.5		32.6
				
**Preschool**				
Number of children in day care group	278	20 (6.8)	200	21 (7.0)

**Table 2:** Mean food consumption on preschool days (Monday to Friday) of 3- to 4-year-old Finnish children who eat three meals at preschool (*n* = 324).

	Outside Preschool		Preschool		Total
	g/Day	%	g/Day	%	g/Day
**Vegetables, vegetable dishes**	**50**	**50**	**50**	**50**	**100**
fresh vegetables and vegetable salads	35	53	32	47	67
vegetarian dishes	9	37	15	63	24
side dish vegetables	5	60	3	40	8
**Potatoes, potato dishes**	**20**	**32**	**42**	**68**	**62**
**Fruit, berries, fruit and berry products**	**93**	**52**	**85**	**48**	**179**
fresh fruit	66	71	27	29	93
berries	4	91	0.4	9	5
fruit and berry soups	6	15	33	85	39
100% juice	9	56	7	44	16
**Cereals, bakery products**	**93**	**34**	**180**	**66**	**273**
rye bread	6	37	11	63	17
rye crispbread	1	12	11	88	12
multi-grain bread	9	31	21	69	30
white bread	3	52	3	48	6
porridge	33	22	115	78	147
rice, pasta, etc.	20	67	10	33	30
buns, sweet bakery products	4	90	0.5	10	5
biscuits and muesli bars	3	84	1	16	4
savory bakery products, hamburgers, pizza	7	76	2	24	10
pancakes, crêpes	3	34	5	66	8
**Fats, oils, gravy**	**7**	**24**	**21**	**76**	**28**
margarine and fat spread	2	12	16	88	18
blended spread	2	100	0	0	2
butter	0.2	100	0	0	0.2
**Fish, fish dishes**	**11**	**30**	**25**	**70**	**35**
**Eggs, egg dishes**	**2**	**51**	**2**	**49**	**5**
**Meat, meat dishes**	**74**	**46**	**87**	**54**	**161**
cold cuts	3	51	2	49	5
red meat dishes	51	51	48	49	99
poultry dishes	15	39	24	61	39
sausage dishes	5	29	12	71	17
**Milk, dairy products**	**236**	**40**	**352**	**60**	**589**
skimmed milk	79	23	265	77	344
milk with 1-1.5% fat content	84	63	50	37	134
whole milk	5	100	0	0	5
sour milk	3	37	5	63	8
milk with cocoa	10	77	3	23	14
yogurt and Finnish cultured milk	37	78	10	22	47
cheese	7	64	4	36	11
dairy-based desserts	7	35	14	65	21
ice-cream	3	84	1	16	4
**Sugar, sweets**	**4**	**81**	**1**	**19**	**5**
**Drinks**	**100**	**68**	**47**	**32**	**147**
water	55	66	29	34	83
sugar-sweetened juice	23	82	5	18	28
sugar-sweetened soda	4	100	0	0	4
non-dairy milk substitutes	10	47	12	53	22
**Miscellaneous**	**7**	**74**	**2**	**26**	**9**

Main food groups (bold) and selected sub-groups are presented.

**Table 3:** Mean food consumption on preschool days (Monday to Friday) of 5- to 6-year-old Finnish children who eat three meals at preschool (*n* = 233).

	Outside Preschool		Preschool		Total
	g/Day	%	g/Day	%	g/Day
**Vegetables, vegetable dishes**	**53**	**46**	**62**	**54**	**115**
fresh vegetables and vegetable salads	38	49	39	51	77
vegetarian dishes	11	37	19	63	29
side dish vegetables	4	49	4	51	8
**Potatoes, potato dishes**	**22**	**30**	**51**	**70**	**73**
**Fruit, berries, fruit and berry products**	**113**	**57**	**86**	**43**	**199**
fresh fruit	64	68	30	32	93
berries	8	94	1	6	9
fruit and berry soups	12	31	28	69	40
100% juice	17	67	9	33	26
**Cereals, bakery products**	**102**	**32**	**212**	**68**	**314**
rye bread	7	43	10	57	17
rye crispbread	1	8	12	92	13
multi-grain bread	11	34	22	66	33
white bread	4	42	5	58	9
porridge	33	19	139	81	172
rice, pasta, etc.	22	66	11	33	34
buns, sweet bakery products	3	83	1	17	4
biscuits and muesli bars	3	70	1	30	4
savory bakery products, hamburgers, pizza	10	72	4	28	14
pancakes, crêpes	2	27	6	73	8
**Fats, oils, gravy**	**7**	**22**	**24**	**78**	**31**
margarine and fat spread	2	11	18	89	20
blended spread	2	99	0	1	2
butter	0.2	100	0	0	0.2
**Fish, fish dishes**	**16**	**37**	**27**	**63**	**42**
**Eggs, egg dishes**	**3**	**57**	**2**	**42**	**5**
**Meat, meat dishes**	**82**	**44**	**103**	**56**	**185**
cold cuts	3	47	3	53	6
red meat dishes	58	50	57	50	115
poultry dishes	15	32	31	68	46
sausage dishes	6	35	11	65	16
**Milk, dairy products**	**258**	**40**	**391**	**60**	**649**
skimmed milk	100	26	284	74	384
milk with 1-1.5% fat content	67	52	63	48	130
whole milk	6	100	0	0	6
sour milk	8	55	6	45	14
milk with cocoa	11	62	7	38	18
yogurt and Finnish cultured milk	44	83	9	17	53
cheese	8	65	4	35	12
dairy-based desserts	10	39	16	61	26
ice-cream	4	77	1	23	5
**Sugar, sweets**	**8**	**83**	**2**	**17**	**10**
**Drinks**	**95**	**64**	**53**	**36**	**148**
water	44	54	37	46	82
sugar-sweetened juice	29	77	9	23	38
sugar-sweetened soda	9	100	0	0	9
non-dairy milk substitutes	7	56	5	44	12
**Miscellaneous**	**7**	**75**	**2**	**25**	**10**

Main food groups (bold) and selected sub-groups are presented.

**Table 4:** Mean (SD) intake and population proportion [24] of energy and nutrients on weekdays among 3 to 4-year-old children who eat three meals at preschool (*n* = 324). Each child had data for 1 to 4 days; the total number of days was 561.

	Breakfast, Outside Preschool	Breakfast, Preschool	Lunch, Preschool	Afternoon Snack, Preschool	Dinner, Outside Preschool	Evening Snack, Outside Preschool	Other Snack, Outside Preschool	Preschool Food, Total	Total, per Day
% of days that included the meal	24	100	100	100	95	91	50		
Energy, MJ	0.13 (0.27)	0.77 (0.29)	1.37 (0.51)	0.90 (0.36)	1.15 (0.51)	0.89 (0.49)	0.37 (0.50)	3.04 (0.83)	5.59 (1.10)
Protein, g	1 (2)	7 (3)	15 (7)	8 (3)	15 (7)	8 (5)	2 (4)	31 (10)	56 (13)
Carbohydrates, g	4 (9)	24 (10)	35 (13)	29 (13)	26 (13)	28 (15)	13 (17)	88 (25)	159 (34)
Sucrose, g	1 (3)	2 (3)	2 (2)	7 (6)	3 (4)	7 (6)	5 (8)	11 (8)	27 (14)
Added sugar, g	1 (3)	1 (3)	1 (2)	6 (6)	2 (4)	6 (6)	5 (8)	8 (7)	21 (13)
Fiber, g	0.4 (0.9)	2.8 (1.5)	3.7 (1.5)	2.6 (1.5)	2.1 (1.4)	2.7 (2.0)	0.9 (1.5)	9.1 (3.1)	15.3 (4.6)
Fat, g	1 (2)	6 (4)	13 (6)	7 (4)	12 (6)	7 (6)	3 (5)	25 (9)	47 (13)
SAFA, g	0.4 (1.1)	1.8 (1.2)	4.1 (2.3)	2.5 (1.7)	4.3 (2.9)	2.9 (2.5)	1.3 (2.3)	8.4 (3.5)	17.3 (5.3)
MUFA, g	0.3 (0.8)	2 (1.5)	4.8 (2.6)	2.3 (1.4)	4.1 (2.5)	2.1 (2.1)	0.9 (1.8)	9.1 (3.7)	16.5 (5.1)
PUFA, g	0.1 (0.4)	1.2 (0.9)	2.5 (1.4)	1.3 (0.9)	1.6 (1.2)	0.9 (1.1)	0.4 (0.7)	4.9 (2.1)	8.0 (2.9)
Vitamin A, μg RAE	12 (52)	71 (56)	144 (126)	96 (104)	191 (517)	73 (104)	26 (79)	311 (185)	613 (568)
Vitamin D, μg	0.2 (0.6)	2.4 (1.2)	2.8 (1.5)	2.2 (1.1)	1.6 (1.5)	1.4 (1.2)	0.3 (0.7)	7.4 (2.8)	10.9 (3.7)
Vitamin E, mg	0.1 (0.4)	1.0 (0.6)	1.8 (1.0)	1.1 (0.7)	1.3 (0.8)	0.9 (0.9)	0.3 (0.6)	3.9 (1.5)	6.5 (2.1)
Thiamine, mg	0.02 (0.04)	0.10 (0.05)	0.27 (0.14)	0.11 (0.05)	0.20 (0.12)	0.12 (0.08)	0.03 (0.06)	0.47 (0.17)	0.84 (0.22)
Riboflavin, mg	0.04 (0.10)	0.29 (0.13)	0.39 (0.15)	0.30 (0.14)	0.37 (0.22)	0.28 (0.19)	0.07 (0.13)	0.98 (0.33)	1.74 (0.51)
Niacin eq., mg	0.4 (0.9)	2.5 (1.1)	5.5 (2.8)	2.4 (1.0)	5.6 (2.9)	2.5 (1.6)	0.7 (1.2)	10.4 (3.6)	19.5 (4.8)
Vitamin B6, mg	0.0 (0.1)	0.1 (0.1)	0.3 (0.2)	0.2 (0.1)	0.3 (0.1)	0.2 (0.2)	0.1 (0.1)	0.6 (0.2)	1.2 (0.3)
Folate, μg	4 (9)	21 (11)	42 (19)	24 (11)	34 (37)	23 (17)	7 (12)	87 (29)	154 (55)
Vitamin B12, μg	0.1 (0.2)	0.6 (0.3)	1.1 (0.7)	0.6 (0.3)	1.5 (2.5)	0.6 (0.9)	0.1 (0.3)	2.3 (1.0)	4.5 (2.9)
Vitamin C, mg	2 (6)	8 (9)	14 (9)	12 (18)	14 (15)	11 (14)	5 (11)	33 (23)	65 (35)
Salt, g	0.1 (0.2)	1.0 (0.5)	1.6 (0.7)	0.6 (0.3)	1.3 (0.7)	0.6 (0.4)	0.2 (0.3)	3.2 (1.1)	5.3 (1.4)
Potassium, mg	62 (130)	338 (126)	813 (332)	371 (152)	583 (303)	373 (211)	117 (177)	1521 (462)	2656 (610)
Phosphorous, g	26 (62)	198 (79)	291 (103)	194 (84)	250 (124)	181 (114)	46 (79)	684 (197)	1187 (281)
Calcium, mg	26 (67)	178 (80)	206 (90)	190 (94)	186 (116)	179 (129)	44 (87)	573 (197)	1007 (306)
Magnesium, mg	6 (14)	42 (17)	65 (22)	39 (17)	47 (23)	38 (23)	12 (18)	147 (41)	250 (54)
Iron, mg	0.2 (0.5)	1.2 (0.6)	1.9 (0.8)	0.9 (0.5)	1.7 (1.1)	1.1 (0.8)	0.3 (0.5)	4.1 (1.4)	7.3 (2.0)
Zinc, mg	0.2 (0.5)	1.2 (0.6)	2.1 (0.9)	1.2 (0.6)	2.0 (1.0)	1.2 (0.8)	0.3 (0.5)	4.5 (1.5)	8.1 (2.0)
Iodine, μg	3 (10)	40 (19)	47 (27)	27 (14)	46 (25)	26 (18)	6 (12)	114 (43)	195 (57)
Energy, %	2	14	24	16	21	16	7	54	100
Protein, %	2	13	27	14	27	13	4	54	100
Carbohydrates, %	3	15	22	18	16	18	8	55	100
Sucrose, %	4	8	8	25	10	26	19	41	100
Added sugar, %	4	7	4	28	9	27	22	39	100
Fiber, %	3	19	25	17	14	18	6	60	100
Fat, %	2	12	27	14	24	15	6	53	100
SAFA, %	2	11	24	14	25	17	8	49	100
MUFA, %	2	12	29	14	25	13	6	55	100
PUFA, %	2	15	30	16	20	12	5	61	100
Vitamin A, %	2	12	23	16	31	12	4	51	100
Vitamin D, %	2	22	25	20	15	13	3	68	100
Vitamin E, %	2	15	28	17	20	13	5	60	100
Thiamine, %	2	12	32	13	24	14	4	56	100
Riboflavin, %	2	17	22	17	21	16	4	56	100
Niacin eq., %	2	13	28	12	29	13	3	53	100
Vitamin B6, %	3	12	28	13	23	16	5	52	100
Folate, %	2	14	27	15	22	15	4	56	100
Vitamin B12, %	2	13	25	13	33	13	3	50	100
Vitamin C, %	3	12	21	18	21	18	7	50	100
Salt, %	1	20	29	11	24	11	3	60	100
Potassium, %	2	13	31	14	22	14	4	57	100
Phosphorous, %	2	17	25	16	21	15	4	58	100
Calcium, %	3	18	20	19	18	18	4	57	100
Magnesium, %	2	17	26	16	19	15	5	59	100
Iron, %	2	16	27	13	23	15	4	56	100
Zinc, %	2	15	26	14	24	14	4	56	100
Iodine, %	2	21	24	14	23	13	3	58	100

eq., equivalents; MUFA, monounsaturated fatty acids; PUFA, polyunsaturated fatty acids; RAE, retinol activity equivalents; SAFA, saturated fatty acids.

**Table 5:** Mean (SD) intake and population proportion [24] of energy and nutrients on weekdays among 5 to 6-year-old children who eat three meals at preschool (*n* = 233). Each child had data for 1 to 4 days; the total number of days was 405.

	Breakfast, Outside Preschool	Breakfast, Preschool	Lunch, Preschool	Afternoon Snack, Preschool	Dinner, Outside Preschool	Evening Snack, Outside Preschool	Other Snack, Outside Preschool	Preschool Food, Total	Total, per Day
% of days that included the meal	27	100	100	100	95	88	50		
Energy, MJ	0.17 (0.35)	0.85 (0.28)	1.58 (0.61)	1.03 (0.42)	1.34 (0.65)	0.93 (0.54)	0.47 (0.58)	3.46 (0.94)	6.37 (1.25)
Protein, g	1 (3)	8 (3)	18 (8)	9 (4)	17 (9)	8 (5)	2 (4)	35 (11)	64 (16)
Carbohydrates, g	6 (10)	27 (10)	40 (14)	33 (15)	31 (17)	29 (18)	17 (22)	99 (27)	182 (42)
Sucrose, g	2 (4)	2 (3)	3 (3)	8 (7)	3 (5)	7 (6)	7 (11)	13 (9)	32 (17)
Added sugar, g	1 (4)	1 (3)	2 (3)	7 (7)	3 (6)	5 (6)	7 (13)	10 (9)	27 (19)
Fiber, g	0.5 (0.9)	3.2 (1.7)	4.2 (1.7)	2.7 (1.4)	2.3 (1.5)	2.9 (2.3)	1.0 (1.5)	10.1 (3.4)	16.7 (4.7)
Fat, g	1 (4)	6 (3)	15 (7)	8 (4)	13 (8)	7 (6)	3 (6)	29 (10)	54 (14)
SAFA, g	0.5 (1.7)	1.9 (1.3)	4.8 (2.5)	3.0 (2.0)	4.8 (3.4)	3.0 (2.6)	1.4 (2.4)	9.8 (4.0)	19.5 (6.2)
MUFA, g	0.4 (1.2)	2.1 (1.3)	5.6 (2.9)	2.8 (1.7)	4.8 (3.1)	2.0 (1.9)	1.1 (2.0)	10.5 (4)	18.8 (5.3)
PUFA, g	0.2 (0.6)	1.2 (0.8)	3.0 (1.8)	1.5 (0.9)	2.0 (1.6)	1.0 (1.1)	0.5 (1.0)	5.7 (2.4)	9.4 (3.1)
Vitamin A, μg RAE	9 (26)	73 (54)	212 (289)	105 (87)	167 (584)	73 (123)	20 (44)	389 (315)	659 (654)
Vitamin D, μg	0.2 (0.7)	2.5 (1.2)	3.1 (2.4)	2.6 (1.3)	1.9 (1.9)	1.5 (1.3)	0.3 (0.7)	8.2 (3.7)	12.1 (4.7)
Vitamin E, mg	0.2 (0.5)	1.0 (0.6)	2.2 (1.4)	1.3 (0.6)	1.5 (1.2)	0.9 (0.9)	0.4 (0.6)	4.5 (1.8)	7.5 (2.4)
Thiamine, mg	0.02 (0.06)	0.11 (0.06)	0.30 (0.17)	0.12 (0.05)	0.23 (0.14)	0.13 (0.10)	0.04 (0.06)	0.53 (0.21)	0.95 (0.28)
Riboflavin, mg	0.05 (0.11)	0.31 (0.14)	0.45 (0.19)	0.34 (0.15)	0.40 (0.27)	0.30 (0.21)	0.08 (0.13)	1.10 (0.37)	1.93 (0.59)
Niacin eq., mg	0.5 (1.3)	2.8 (1.1)	6.2 (3.5)	2.7 (1.2)	6.2 (3.2)	2.8 (1.9)	0.8 (1.3)	11.7 (4.3)	22.0 (6.3)
Vitamin B6, mg	0.0 (0.1)	0.2 (0.1)	0.4 (0.2)	0.2 (0.1)	0.3 (0.2)	0.2 (0.2)	0.1 (0.2)	0.7 (0.3)	1.3 (0.4)
Folate, μg	5 (10)	24 (14)	51 (39)	25 (11)	35 (43)	26 (18)	8 (11)	100 (48)	174 (67)
Vitamin B12, μg	0.1 (0.3)	0.6 (0.4)	1.3 (1.1)	0.7 (0.3)	1.5 (2.9)	0.6 (0.4)	0.1 (0.3)	2.5 (1.3)	4.8 (3.4)
Vitamin C, mg	3 (10)	9 (11)	16 (9)	11 (9)	14 (13)	15 (20)	7 (14)	35 (19)	74 (38)
Salt, g	0.1 (0.4)	1.2 (0.5)	1.8 (0.8)	0.7 (0.3)	1.4 (0.7)	0.7 (0.5)	0.2 (0.3)	3.6 (1.2)	6.0 (1.6)
Potassium, mg	74 (155)	381 (148)	935 (399)	410 (167)	661 (345)	403 (264)	136 (188)	1726 (557)	2999 (797)
Phosphorous, g	32 (78)	217 (80)	333 (135)	217 (95)	280 (148)	196 (130)	50 (78)	767 (231)	1326 (347)
Calcium, mg	29 (74)	189 (89)	232 (100)	211 (114)	210 (142)	192 (141)	49 (85)	632 (228)	1113 (358)
Magnesium, mg	7 (14)	47 (17)	74 (28)	43 (18)	52 (26)	40 (28)	14 (19)	164 (46)	278 (65)
Iron, mg	0.2 (0.4)	1.3 (0.7)	2.2 (1.1)	1.0 (0.5)	1.9 (1.5)	1.1 (0.8)	0.4 (0.6)	4.6 (1.6)	8.2 (2.4)
Zinc, mg	0.2 (0.5)	1.4 (0.6)	2.4 (1.2)	1.3 (0.6)	2.3 (1.3)	1.3 (0.8)	0.3 (0.5)	5.0 (1.8)	9.1 (2.5)
Iodine, μg	5 (12)	45 (19)	53 (34)	31 (14)	52 (31)	28 (21)	7 (12)	128 (48)	220 (69)
Energy, %	3	13	25	16	21	15	7	54	100
Protein, %	2	13	28	14	27	13	4	55	100
Carbohydrates, %	3	15	22	18	17	16	9	54	100
Sucrose, %	5	7	9	24	11	22	22	39	100
Added sugar, %	5	6	6	26	10	21	27	37	100
Fiber, %	3	19	25	16	13	17	6	61	100
Fat, %	2	11	28	15	25	13	6	54	100
SAFA, %	3	10	25	15	25	15	7	50	100
MUFA, %	2	11	30	15	26	11	6	56	100
PUFA, %	2	13	32	16	21	10	5	61	100
Vitamin A, %	1	11	32	16	25	11	3	59	100
Vitamin D, %	2	20	26	21	16	12	2	68	100
Vitamin E, %	2	14	29	17	20	12	5	60	100
Thiamine, %	2	12	31	12	24	14	4	56	100
Riboflavin, %	3	16	23	17	21	16	4	57	100
Niacin eq., %	2	13	28	12	28	13	4	53	100
Vitamin B6, %	3	12	28	13	23	14	7	53	100
Folate, %	3	14	29	14	20	15	5	58	100
Vitamin B12, %	2	13	26	14	31	12	2	53	100
Vitamin C, %	5	12	21	14	19	20	9	48	100
Salt, %	2	19	29	12	23	11	3	60	100
Potassium, %	2	13	31	14	22	13	5	58	100
Phosphorous, %	2	16	25	16	21	15	4	58	100
Calcium, %	3	17	21	19	19	17	4	57	100
Magnesium, %	3	17	27	15	19	15	5	59	100
Iron, %	2	16	27	13	23	13	5	56	100
Zinc, %	2	15	26	14	25	14	4	55	100
Iodine, %	2	20	24	14	24	13	3	58	100

eq., equivalents; MUFA, monounsaturated fatty acids; PUFA, polyunsaturated fatty acids; RAE, retinol activity equivalents; SAFA, saturated fatty acids.

**Table 6:** Energy-yielding nutrient intakes [mean, (SD)] during preschool hours and the whole day (Monday to Friday) of children who eat three meals at preschool.

	Finnish Recommendation for Served Preschool Meals	3- to 4-Year-Olds (*n* = 324)		5- to 6-Year-Olds (*n* = 233)	
		Preschool	Total	Preschool	Total
Carbohydrates, E%	45–60	49 (6)	49 (5)	49 (5)	49 (5)
Sucrose, E%		6.1 (4)	8.1 (3.8)	6.2 (3.9)	8.5 (4.1)
Added sugar, E%	<10	4.5 (3.6)	6.2 (3.5)	4.8 (3.8)	7.0 (4.6)
Protein, E%	10–15	17 (3)	17 (2)	17 (3)	17 (3)
Fat, E%	30–40	30 (7)	31 (5)	31 (6)	31 (5)
Saturated fatty acids, E%	<10	10.1 (2.8)	11.4 (2.4)	10.3 (2.6)	11.3 (2.6)
Monounsaturated fatty acids, E%		11.0 (2.8)	10.9 (2.1)	11.1 (2.5)	10.9 (2.1)
Polyunsaturated fatty acids, E%		6.0 (1.8)	5.3 (1.3)	6.1 (1.7)	5.4 (1.4)
Fiber g/MJ		3.0 (0.8)	2.7 (0.7)	3.0 (0.7)	2.7 (0.7)

E%, percentage of the total energy.

## Supplementary Materials

The following are available online at https://www.mdpi.com/2072-6643/11/7/1531/s1: Tables S1 and S2: Mean (SD) intake and population proportion of energy and nutrients on weekdays among 3- to 4-year-old/5- to 6-year-old children who eat three meals at preschool. Results for days when the child ate the meal. Tables S3–S58: Sources of energy and nutrients (mean and population proportion) in the diet on weekdays among Finnish 3- to 4-year-old/5- to 6-year-old preschool children who eat three meals at preschool. Main food groups and selected food groups are presented.

## References

[1] Mikkilä V., Räsänen L., Raitakari O., Pietinen P., Viikari J. (2005). Consistent dietary patterns identified from childhood to adulthood: The cardiovascular risk in Young Finns Study. Br. J. Nutr..

[2] Craigie A.M., Lake A.A., Kelly S.A., Adamson A.J., Mathers J.C. (2011). Tracking of obesity-related behaviours from childhood to adulthood: A systematic review. Maturitas.

[3] Daniels S.R., Arnett D.K., Eckel R.H., Gidding S.S., Hayman L.L., Kumanyika S., Robinson T.N., Scott B.J., St Jeor S., Williams C.L. (2005). Overweight in children and adolescents: Pathophysiology, consequences, prevention, and treatment. Circulation.

[4] Kaikkonen J.E., Mikkilä V., Raitakari O.T. (2014). Role of childhood food patterns on adult cardiovascular disease risk. Curr. Atheroscler. Rep..

[5] Briley M.E., Jastrow S., Vickers J., Roberts-Gray C. (1999). Dietary intake at child-care centers and away: Are parents and care providers working as partners or at cross-purposes?. J. Am. Diet. Assoc..

[6] Sepp H., Lennernäs M., Pettersson R., Abrahamsson L. (2001). Children’s nutrient intake at preschool and at home. Acta Paediatr..

[7] Padget A., Briley M.E. (2005). Dietary intakes at child-care centers in central Texas fail to meet Food Guide Pyramid recommendations. J. Am. Diet. Assoc..

[8] Gubbels J., Raaijmakers L., Gerards S., Kremers S. (2014). Dietary intake by Dutch 1-to 3-year-old children at childcare and at home. Nutrients.

[9] Sisson S.B., Kiger A.C., Anundson K.C., Rasbold A.H., Krampe M., Campbell J., DeGrace B., Hoffman L. (2017). Differences in preschool-age children’s dietary intake between meals consumed at childcare and at home. Prev. Med. Rep..

[10] Lehtisalo J., Erkkola M., Tapanainen H., Kronberg-Kippilä C., Veijola R., Knip M., Virtanen S.M. (2010). Food consumption and nutrient intake in day care and at home in 3-year-old Finnish children. Public Health Nutr..

[11] Ziegler P., Briefel R., Ponza M., Novak T., Hendricks K. (2006). Nutrient intakes and food patterns of toddlers’ lunches and snacks: Influence of location. J. Am. Diet. Assoc..

[12] National Institute of Health and Welfare (2018). Tilastoraportti (Statistics Report) 32/2018. http://urn.fi/URN:NBN:fi-fe2018100937865.

[13] Act on Early Childhood Education and Care (540/2018). https://www.finlex.fi/fi/laki/ajantasa/2018/20180540.

[14] Finnish National Board of Education Regulation (2016). National Core Curriculum for Early Childhood Education and Care. https://www.ellibs.com/fi/book/9789521363290/national-corecurriculum-for-early-childhood-education-and-care-2016.

[15] National Nutrition Council (2018). Health and Joy from Food—Meal Recommendations for Early Childhood Education and Care.

[16] National Nutrition Council (2016). Eating Together—Food Recommendations for Families with Children.

[17] Hasunen K., Kalavainen M., Keinonen H., Lagström H., Lyytikäinen A., Nurttila A., Peltola T., Talvia S. (2004). Lapsi, perhe ja ruoka. Nutrition recommendations for infants and young children.

[18] Lehto E., Ray C., Vepsäläinen H., Korkalo L., Lehto R., Kaukonen R., Suhonen E., Nislin M., Nissinen K., Skaffari E. (2018). Increased Health and Wellbeing in Preschools (DAGIS) Study—Differences in Children’s Energy Balance-Related Behaviors (EBRBs) and in Long-Term Stress by Parental Educational Level. Int. J. Environ. Res. Public Health.

[19] Cole T.J., Lobstein T. (2012). Extended international (IOTF) body mass index cut-offs for thinness, overweight and obesity. Pediatr. Obes..

[20] Nissinen K., Korkalo L., Vepsäläinen H., Mäkiranta P., Koivusilta L., Roos E., Erkkola M. (2018). Accuracy in the estimation of children’s food portion sizes against a food picture book by parents and early educators. J. Nutr. Sci..

[21] Nissinen K., Sillanpää H., Korkalo L., Roos E., Erkkola M. (2015). Annoskuvakirja Lasten Ruokamäärien Arvioinnin Avuksi (The Children’s Food Picture Book).

[22] Vásquez-Caicedo A., Bell S., Hartmann B. Report on Collection of Rules on Use of Recipe Calculation Procedures including the Use of Yield and Retention Factors for Imputing Nutrient Values for Composite Foods. http://www.webcitation.org/78Kr2gJr7.

[23] Lehto R., Ray C., Vepsäläinen H., Korkalo L., Nissinen K., Skaffari E., Määttä S., Roos E., Erkkola M. (2019). Early educators’ practices and opinions in relation to pre-schoolers’ dietary intake at pre-school: Case Finland. Public Health Nutr..

[24] Krebs-Smith S., Kott P., Guenther P. (1989). Mean proportion and population proportion: Two answers to the same question?. J. Am. Diet. Assoc..

[25] REGULATION (EU) No 1308/2013 OF THE EUROPEAN PARLIAMENT AND OF THE COUNCIL of 17 December 2013 Establishing a Common Organisation of the Markets in Agricultural Products and Repealing Council Regulations (EEC) No 922/72, (EEC) No 234/79, (EC) No 1037/2001 and (EC) No 1234/2007. https://eur-lex.europa.eu/legal-content/EN/TXT/PDF/?uri=CELEX:32013R1308&rid=1.

[26] Goldbohm R., Rubingh C., Lanting C., Joosten K. (2016). Food consumption and nutrient intake by children aged 10 to 48 months attending day care in the Netherlands. Nutrients.

[27] National Institute for Health and Welfare Fineli Food Composition Database. https://fineli.fi.

[28] Huybrechts I., De Henauw S. (2007). Energy and nutrient intakes by pre-school children in Flanders-Belgium. Br. J. Nutr..

[29] Moreira T., Severo M., Oliveira A., Ramos E., Rodrigues S., Lopes C. (2015). Eating out of home and dietary adequacy in preschool children. Br. J. Nutr..

[30] Lucas P., Patterson E., Sacks G., Billich N., Evans C. (2017). Preschool and school meal policies: An overview of what we know about regulation, implementation, and impact on diet in the UK, Sweden, and Australia. Nutrients.

