# Supplementary material for: The Contribution of Preschool Meals to the Diet of Finnish Preschoolers
Source: Nutrients. 2019 Jul 5;11(7):1531. doi: 10.3390/nu11071531 (PMC6683016; doi:10.3390/nu11071531)
Supplement: Supplementary file 1 [file nutrients-11-01531-s001.pdf]

Table S 1: Mean (SD) intake and population proportion of energy and nutrients on weekdays among 3- to 4-year-old children who eat three meals at preschool. Results for days when the child ate the meal.

	**Breakfast, outside preschool n=94**	**Breakfast, preschool n=324**	**Lunch, preschool n=324**	**Afternoon snack, preschool n=324**	**Dinner, outside preschool n=317**	**Evening snack, outside preschool n=306**	**Other snack, outside preschool n=204**
Energy, MJ	0.50 (0.33)	0.77 (0.29)	1.37 (0.51)	0.90 (0.36)	1.21 (0.47)	0.98 (0.45)	0.76 (0.61)
Protein, g	4 (3)	7 (3)	15 (7)	8 (3)	16 (7)	8 (5)	4 (4)
Carbohydrate, g	17 (10)	24 (10)	35 (13)	29 (13)	27 (12)	31 (14)	26 (21)
Sucrose, g	4 (4)	2 (3)	2 (2)	7 (6)	3 (4)	8 (6)	11 (11)
Added sugar, g	3 (5)	1 (3)	1 (2)	6 (6)	2 (4)	6 (7)	10 (12)
Fiber, g	1.6 (1.3)	2.8 (1.5)	3.7 (1.5)	2.6 (1.5)	2.2 (1.4)	3 (1.9)	1.8 (1.8)
Fat, g	3 (4)	6 (4)	13 (6)	7 (4)	12 (6)	8 (6)	6 (7)
SAFA, g	1.5 (1.7)	1.8 (1.2)	4.1 (2.3)	2.5 (1.7)	4.5 (2.9)	3.2 (2.5)	2.7 (3.2)
MUFA, g	1 (1.3)	2.0 (1.5)	4.8 (2.6)	2.3 (1.4)	4.3 (2.4)	2.3 (2.1)	1.9 (2.3)
PUFA, g	0.5 (0.7)	1.2 (0.9)	2.5 (1.4)	1.3 (0.9)	1.7 (1.3)	1.1 (1.1)	0.7 (1.0)
Vitamin A, μg RAE	49 (102)	71 (56)	144 (126)	96 (104)	198 (522)	79 (106)	52 (109)
Vitamin D, μg	0.8 (0.9)	2.4 (1.2)	2.8 (1.5)	2.2 (1.1)	1.7 (1.5)	1.5 (1.3)	0.6 (1.0)
Vitamin E, mg	0.5 (0.7)	1.0 (0.6)	1.8 (1.0)	1.1 (0.7)	1.4 (0.9)	0.9 (0.9)	0.7 (0.7)
Thiamine, mg	0.06 (0.06)	0.10 (0.05)	0.27 (0.14)	0.11 (0.05)	0.21 (0.11)	0.13 (0.08)	0.06 (0.07)
Riboflavin, mg	0.16 (0.15)	0.29 (0.13)	0.39 (0.15)	0.3 (0.14)	0.38 (0.21)	0.3 (0.19)	0.14 (0.16)
Niacin equivalents, mg	1.4 (1.2)	2.5 (1.1)	5.5 (2.8)	2.4 (1.0)	5.8 (2.8)	2.7 (1.6)	1.3 (1.4)
Vitamin B6, mg	0.1 (0.1)	0.1 (0.1)	0.3 (0.2)	0.2 (0.1)	0.3 (0.1)	0.2 (0.2)	0.1 (0.2)
Folate, μg	14 (13)	21 (11)	42 (19)	24 (11)	35 (37)	25 (17)	14 (15)
Vitamin B12,μg	0.3 (0.3)	0.6 (0.3)	1.1 (0.7)	0.6 (0.3)	1.5 (2.6)	0.6 (0.9)	0.2 (0.4)
Vitamin C, mg	9 (11)	8 (9)	14 (9)	12 (18)	15 (15)	13 (14)	10 (16)
Salt, g	0.3 (0.3)	1.0 (0.5)	1.6 (0.7)	0.6 (0.3)	1.3 (0.6)	0.7 (0.4)	0.3 (0.4)
Potassium, mg	241 (152)	338 (126)	813 (332)	371 (152)	609 (287)	409 (200)	227 (199)
Phosphorous, g	98 (87)	198 (79)	291 (103)	194 (84)	261 (117)	198 (108)	89 (96)
Calcium, mg	100 (99)	178 (80)	206 (90)	190 (94)	193 (115)	195 (126)	84 (109)
Magnesium, mg	24 (18)	42 (17)	65 (22)	39 (17)	49 (21)	42 (21)	23 (21)
Iron, mg	0.6 (0.7)	1.2 (0.6)	1.9 (0.8)	0.9 (0.5)	1.7 (1.1)	1.2 (0.8)	0.6 (0.6)
Zinc, mg	0.7 (0.7)	1.2 (0.6)	2.1 (0.9)	1.2 (0.6)	2.0 (1.0)	1.3 (0.7)	0.6 (0.7)
Iodine, μg	13 (15)	40 (19)	47 (27)	27 (14)	48 (24)	29 (18)	12 (16)

Table S 2: Mean (SD) intake and population proportion of energy and nutrients on weekdays among 5- to 6-year-old children who eat three meals at preschool. Results for days when the child ate the meal.

	**Breakfast, outside preschool** n=76	**Breakfast, preschool** n=233	**Lunch, preschool** n=233	**Afternoon snack, preschool** n=233	**Dinner, outside preschool** n=224	**Evening snack, outside preschool** n=222	**Other snack, outside preschool** n=148
Energy, MJ	0.57 (0.40)	0.85 (0.28)	1.58 (0.61)	1.03 (0.42)	1.42 (0.59)	1.05 (0.49)	0.92 (0.67)
Protein, g	4 (4)	8 (3)	18 (8)	9 (4)	18 (8)	9 (5)	4 (5)
Carbohydrate, g	19 (11)	27 (10)	40 (14)	33 (15)	33 (16)	33 (17)	33 (26)
Sucrose, g	5 (6)	2 (3)	3 (3)	8 (7)	4 (6)	8 (6)	14 (14)
Added sugar, g	4 (6)	1 (3)	2 (3)	7 (7)	3 (6)	6 (6)	14 (17)
Fiber, g	1.6 (1.1)	3.2 (1.7)	4.2 (1.7)	2.7 (1.4)	2.4 (1.4)	3.2 (2.2)	1.9 (1.9)
Fat, g	4 (6)	6 (3)	15 (7)	8 (4)	14 (8)	8 (6)	7 (7)
SAFA, g	1.8 (2.6)	1.9 (1.3)	4.8 (2.5)	3.0 (2.0)	5.1 (3.3)	3.3 (2.7)	2.8 (3.1)
MUFA, g	1.3 (1.9)	2.1 (1.3)	5.6 (2.9)	2.8 (1.7)	5.1 (3)	2.3 (1.9)	2.2 (2.6)
PUFA, g	0.6 (0.9)	1.2 (0.8)	3 (1.8)	1.5 (0.9)	2.1 (1.6)	1.1 (1.2)	1.0 (1.3)
Vitamin A,μg RAE	31 (39)	73 (54)	212 (289)	105 (87)	176 (594)	82 (129)	41 (65)
Vitamin D,μg	0.8 (1.1)	2.5 (1.2)	3.1 (2.4)	2.6 (1.3)	2 (1.9)	1.7 (1.3)	0.6 (1.0)
Vitamin E, mg	0.6 (0.7)	1.0 (0.6)	2.2 (1.4)	1.3 (0.6)	1.7 (1.3)	1.0 (1.0)	0.8 (0.8)
Thiamine, mg	0.08 (0.09)	0.11 (0.06)	0.3 (0.17)	0.12 (0.05)	0.24 (0.13)	0.15 (0.11)	0.07 (0.07)
Riboflavin, mg	0.16 (0.16)	0.31 (0.14)	0.45 (0.19)	0.34 (0.15)	0.43 (0.26)	0.35 (0.22)	0.15 (0.17)
Niacin equivalents, mg	1.6 (1.9)	2.8 (1.1)	6.2 (3.5)	2.7 (1.2)	6.6 (3)	3.2 (1.9)	1.6 (1.6)
Vitamin B6, mg	0.1 (0.1)	0.2 (0.1)	0.4 (0.2)	0.2 (0.1)	0.3 (0.1)	0.2 (0.2)	0.2 (0.2)
Folate, μg	16 (15)	24 (14)	51 (39)	25 (11)	37 (43)	30 (19)	16 (15)
Vitamin B12, μg	0.3 (0.6)	0.6 (0.4)	1.3 (1.1)	0.7 (0.3)	1.6 (3.0)	0.6 (0.4)	0.2 (0.4)
Vitamin C, mg	12 (17)	9 (11)	16 (9)	11 (9)	15 (13)	17 (23)	12 (21)
Salt, g	0.4 (0.6)	1.2 (0.5)	1.8 (0.8)	0.7 (0.3)	1.5 (0.7)	0.8 (0.5)	0.4 (0.4)
Potassium, mg	245 (191)	381 (148)	935 (399)	410 (167)	704 (315)	456 (253)	266 (227)
Phosphorous, g	108 (110)	217 (80)	333 (135)	217 (95)	298 (136)	222 (125)	100 (102)
Calcium, mg	97 (108)	189 (89)	232 (100)	211 (114)	224 (138)	219 (144)	98 (115)
Magnesium, mg	24 (17)	47 (17)	74 (28)	43 (18)	55 (23)	46 (26)	28 (22)
Iron, mg	0.7 (0.6)	1.3 (0.7)	2.2 (1.1)	1.0 (0.5)	2.0 (1.5)	1.2 (0.8)	0.8 (0.7)
Zinc, mg	0.7 (0.7)	1.4 (0.6)	2.4 (1.2)	1.3 (0.6)	2.4 (1.2)	1.4 (0.8)	0.7 (0.7)
Iodine, μg	15 (19)	45 (19)	53 (34)	31 (14)	56 (30)	32 (21)	13 (16)

Table S 3: Sources of energy (mean and population proportion) in the diet on weekdays among Finnish 3- to 4-year-old preschool children who eat three meals at preschool. Main food groups and selected food groups are presented.

	Outside preschool		Preschool		Total	
	kJ/day	%	kJ/day	%	kJ/day	%
**Vegetables, vegetable dishes**	**102**	**4.0**	**106**	**3.5**	**208**	**3.7**
**Potatoes, potato dishes**	**80**	**3.2**	**133**	**4.4**	**213**	**3.8**
**Fruit, berries, fruit and berry products**	**213**	**8.4**	**203**	**6.7**	**416**	**7.4**
fresh fruit	151	5.9	62	2.0	213	3.8
fruit and berry soups	8	0.3	73	2.4	80	1.4
100% juice	15	0.6	13	0.4	29	0.5
**Cereals, bakery products**	**672**	**26.4**	**887**	**29.2**	**1559**	**27.9**
rye bread	66	2.6	112	3.7	178	3.2
rye crispbread	19	0.7	125	4.1	143	2.6
multi-grain bread	99	3.9	220	7.2	319	5.7
white bread	33	1.3	30	1.0	62	1.1
porridge	84	3.3	247	8.1	331	5.9
rice, pasta, etc.	95	3.7	44	1.4	139	2.5
buns, sweet bakery	65	2.6	8	0.3	74	1.3
biscuits and muesli bars	61	2.4	12	0.4	72	1.3
savoury bakery products, hamburgers, pizza	73	2.9	23	0.8	96	1.7
pancakes, crêpes	22	0.9	37	1.2	59	1.0
**Fats, oils, gravy**	**134**	**5.2**	**382**	**12.6**	**516**	**9.2**
margarine and fat spread	50	2.0	363	11.9	414	7.4
blended spread	63	2.5	0	0.0	63	1.1
**Fish, fish dishes**	**74**	**2.9**	**154**	**5.1**	**228**	**4.1**
**Eggs, egg dishes**	**17**	**0.7**	**14**	**0.5**	**31**	**0.5**
**Meat, meat dishes**	**458**	**18.0**	**474**	**15.6**	**932**	**16.7**
cold cuts	17	0.7	14	0.5	31	0.6
red meat dishes	293	11.5	257	8.4	550	9.8
poultry dishes	101	4.0	112	3.7	213	3.8
sausage dishes	45	1.8	88	2.9	133	2.4
**Milk, dairy products**	**587**	**23.1**	**628**	**20.7**	**1215**	**21.7**
skimmed milk	111	4.4	373	12.3	484	8.7
milk with 1-1.5% fat content	157	6.2	90	2.9	246	4.4
yoghurt and Finnish cultured milk	125	4.9	32	1.1	157	2.8
cheese	81	3.2	43	1.4	124	2.2
dairy-based desserts	35	1.4	67	2.2	102	1.8
ice-cream	29	1.1	6	0.2	35	0.6
**Sugar, sweets**	**70**	**2.7**	**13**	**0.4**	**83**	**1.5**
**Drinks**	**76**	**3.0**	**32**	**1.0**	**108**	**1.9**
sugar-sweetened juice	43	1.7	9	0.3	53	0.9
sugar-sweetened soda	6	0.2	0	0.0	6	0.1
non-dairy milk substitutes	18	0.7	22	0.7	40	0.7
**Miscellaneous**	**62**	**2.5**	**16**	**0.5**	**79**	**1.4**
**Total**	**2546**	**100.0**	**3041**	**100.0**	**5587**	**100.0**

Table S 4: Sources of energy (mean and population proportion) in the diet on weekdays among Finnish 5- to 6-year-old preschool children who eat three meals at preschool. Main food groups and selected food groups are presented.

	Outside preschool		Preschool		Total	
	kJ/day	%	kJ/day	%	kJ/day	%
**Vegetables, vegetable dishes**	**97**	**3.3**	**130**	**3.7**	**227**	**3.6**
**Potatoes, potato dishes**	**110**	**3.8**	**160**	**4.6**	**270**	**4.2**
**Fruit, berries, fruit and berry products**	**250**	**8.6**	**205**	**5.9**	**455**	**7.1**
fresh fruit	146	5.0	67	1.9	212	3.3
fruit and berry soups	18	0.6	61	1.8	79	1.2
100% juice	23	0.8	16	0.5	40	0.6
**Cereals, bakery products**	**727**	**25.0**	**1022**	**29.5**	**1749**	**27.5**
rye bread	77	2.6	99	2.9	176	2.8
rye crispbread	13	0.5	137	3.9	150	2.4
multi-grain bread	121	4.2	230	6.6	351	5.5
white bread	41	1.4	56	1.6	98	1.5
porridge	83	2.9	299	8.6	383	6.0
rice, pasta, etc.	103	3.5	52	1.5	155	2.4
buns, sweet bakery	52	1.8	12	0.4	64	1.0
biscuits and muesli bars	50	1.7	22	0.6	72	1.1
savoury bakery products, hamburgers, pizza	100	3.4	40	1.2	140	2.2
pancakes, crêpes	15	0.5	41	1.2	56	0.9
**Fats, oils, gravy**	**137**	**4.7**	**435**	**12.6**	**572**	**9.0**
margarine and fat spread	49	1.7	412	11.9	461	7.2
blended spread	59	2.0	1	0.0	60	0.9
**Fish, fish dishes**	**88**	**3.0**	**163**	**4.7**	**251**	**3.9**
**Eggs, egg dishes**	**17**	**0.6**	**11**	**0.3**	**28**	**0.4**
**Meat, meat dishes**	**531**	**18.3**	**563**	**16.3**	**1095**	**17.2**
cold cuts	20	0.7	19	0.5	39	0.6
red meat dishes	343	11.8	309	8.9	652	10.2
poultry dishes	108	3.7	153	4.4	261	4.1
sausage dishes	57	2.0	72	2.1	130	2.0
**Milk, dairy products**	**650**	**22.4**	**700**	**20.2**	**1350**	**21.2**
skimmed milk	140	4.8	400	11.5	539	8.5
milk with 1-1.5% fat content	126	4.3	113	3.3	238	3.7
yoghurt and Finnish cultured milk	149	5.1	26	0.8	175	2.7
cheese	90	3.1	46	1.3	135	2.1
dairy-based desserts	46	1.6	81	2.3	127	2.0
ice-cream	37	1.3	12	0.3	49	0.8
**Sugar, sweets**	**137**	**4.7**	**25**	**0.7**	**162**	**2.5**
**Drinks**	**83**	**2.8**	**27**	**0.8**	**110**	**1.7**
sugar-sweetened juice	55	1.9	17	0.5	72	1.1
sugar-sweetened soda	14	0.5	0	0.0	14	0.2
non-dairy milk substitutes	13	0.4	10	0.3	23	0.4
**Miscellaneous**	**80**	**2.7**	**22**	**0.6**	**102**	**1.6**
**Total**	**2907**	**100.0**	**3464**	**100.0**	**6371**	**100.0**

Table S 5: Sources of protein (mean and population proportion) in the diet on weekdays among Finnish 3- to 4-year-old preschool children who eat three meals at preschool. Main food groups and selected food groups are presented.

	**Outside preschool**		**Preschool**		**Total**	
	**g/day**	**%**	**g/day**	**%**	**g/day**	**%**
**Vegetables, vegetable dishes**	**0.8**	**3.3**	**0.8**	**2.7**	**1.7**	**3.0**
**Potatoes, potato dishes**	**0.4**	**1.7**	**0.7**	**2.4**	**1.1**	**2.0**
**Fruit, berries, fruit and berry products**	**0.6**	**2.2**	**0.5**	**1.7**	**1.1**	**1.9**
**Cereals, bakery products**	**4.6**	**17.9**	**6.9**	**22.4**	**11.4**	**20.4**
rye bread	0.5	2.1	0.9	3.1	1.5	2.6
rye crispbread	0.1	0.6	1.0	3.3	1.2	2.1
multi-grain bread	0.8	2.9	1.7	5.7	2.5	4.4
white bread	0.2	0.9	0.2	0.7	0.4	0.8
porridge	0.8	3.0	2.0	6.4	2.7	4.8
rice, pasta, etc.	0.7	2.6	0.3	1.1	1.0	1.8
**Fats, oils, gravy**	**0.1**	**0.3**	**0.0**	**0.1**	**0.1**	**0.2**
**Fish, fish dishes**	**1.4**	**5.6**	**2.5**	**8.2**	**3.9**	**7.0**
**Eggs, egg dishes**	**0.3**	**1.3**	**0.3**	**0.9**	**0.6**	**1.1**
**Meat, meat dishes**	**7.9**	**30.8**	**6.6**	**21.7**	**14.5**	**25.8**
cold cuts	0.5	1.8	0.5	1.8	1.0	1.8
red meat dishes	4.6	18.1	3.5	11.3	8.1	14.4
poultry dishes	2.3	8.8	1.7	5.5	3.9	7.0
sausage dishes	0.5	1.9	0.9	3.0	1.4	2.5
**Milk, dairy products**	**8.8**	**34.3**	**12.0**	**39.1**	**20.8**	**37.0**
skimmed milk	2.5	9.6	8.2	26.9	10.7	19.0
milk with 1-1.5% fat content	2.6	10.1	1.7	5.5	4.3	7.6
yoghurt and Finnish cultured milk	1.2	4.7	0.4	1.1	1.6	2.8
cheese	1.5	5.9	0.9	2.8	2.4	4.2
dairy-based desserts	0.4	1.4	0.6	1.8	0.9	1.6
**Sugar, sweets**	**0.2**	**0.6**	**0.0**	**0.1**	**0.2**	**0.3**
**Drinks**	**0.2**	**0.8**	**0.1**	**0.4**	**0.3**	**0.6**
**Miscellaneous**	**0.3**	**1.4**	**0.1**	**0.3**	**0.4**	**0.8**
**Total**	**25.6**	**100.0**	**30.6**	**100.0**	**56.2**	**100.0**

Table S 6: Sources of protein (mean and population proportion) in the diet on weekdays among Finnish 5- to 6-year-old preschool children who eat three meals at preschool. Main food groups and selected food groups are presented.

	Outside preschool		Preschool		Total	
	g/day	%	g/day	%	g/day	%
**Vegetables, vegetable dishes**	**0.8**	**2.9**	**1.0**	**3.0**	**1.9**	**2.9**
**Potatoes, potato dishes**	**0.5**	**1.8**	**0.8**	**2.4**	**1.4**	**2.2**
**Fruit, berries, fruit and berry products**	**0.7**	**2.3**	**0.5**	**1.5**	**1.2**	**1.9**
**Cereals, bakery products**	**5.2**	**18.0**	**7.6**	**21.9**	**12.8**	**20.1**
rye bread	0.6	2.2	0.8	2.4	1.5	2.3
rye crispbread	0.1	0.3	1.1	3.2	1.2	1.9
multi-grain bread	0.9	3.2	1.8	5.3	2.8	4.3
white bread	0.3	1.0	0.4	1.1	0.7	1.0
porridge	0.8	2.7	2.2	6.3	3.0	4.7
rice, pasta, etc.	0.7	2.6	0.4	1.1	1.1	1.8
**Fats, oils, gravy**	**0.1**	**0.4**	**0.1**	**0.1**	**0.2**	**0.3**
**Fish, fish dishes**	**1.6**	**5.7**	**2.7**	**7.9**	**4.4**	**6.9**
**Eggs, egg dishes**	**0.3**	**1.1**	**0.2**	**0.7**	**0.6**	**0.9**
**Meat, meat dishes**	**9.0**	**31.3**	**8.1**	**23.3**	**17.1**	**26.9**
cold cuts	0.6	2.0	0.7	2.0	1.3	2.0
red meat dishes	5.5	19.1	4.4	12.5	9.8	15.5
poultry dishes	2.3	7.9	2.2	6.3	4.5	7.0
sausage dishes	0.6	2.2	0.7	2.0	1.3	2.1
**Milk, dairy products**	**9.7**	**33.8**	**13.4**	**38.5**	**23.2**	**36.4**
skimmed milk	3.2	11.0	8.9	25.4	12.0	18.9
milk with 1-1.5% fat content	2.1	7.1	2.1	6.0	4.1	6.5
yoghurt and Finnish cultured milk	1.4	4.9	0.3	0.8	1.7	2.7
cheese	1.7	5.9	1.0	2.8	2.7	4.2
dairy-based desserts	0.5	1.8	0.8	2.2	1.3	2.0
**Sugar, sweets**	**0.3**	**0.9**	**0.0**	**0.1**	**0.3**	**0.5**
**Drinks**	**0.1**	**0.5**	**0.1**	**0.2**	**0.2**	**0.3**
**Miscellaneous**	**0.4**	**1.3**	**0.1**	**0.3**	**0.5**	**0.8**
**Total**	**28.8**	**100.0**	**34.9**	**100.0**	**63.7**	**100.0**

Table S 7: Sources of carbohydrates (mean and population proportion) in the diet on weekdays among Finnish 3- to 4-year-old preschool children who eat three meals at preschool. Main food groups and selected food groups are presented.

	**Outside preschool**		**Preschool**		**Total**	
	**g/day**	**%**	**g/day**	**%**	**g/day**	**%**
**Vegetables, vegetable dishes**	**2.8**	**4.0**	**3.3**	**3.7**	**6.1**	**3.9**
**Potatoes, potato dishes**	**3.2**	**4.5**	**6.1**	**6.9**	**9.3**	**5.8**
**Fruit, berries, fruit and berry products**	**10.3**	**14.4**	**10.1**	**11.6**	**20.5**	**12.9**
fresh fruit	7.3	10.3	3.0	3.4	10.3	6.5
fruit and berry soups	0.4	0.6	4.0	4.6	4.4	2.8
100% juice	0.8	1.2	0.7	0.8	1.6	1.0
**Cereals, bakery products**	**26.2**	**36.7**	**36.1**	**41.1**	**62.2**	**39.1**
rye bread	2.8	4.0	4.8	5.5	7.7	4.8
rye crispbread	0.7	1.0	4.8	5.5	5.6	3.5
multi-grain bread	4.1	5.7	9.0	10.3	13.1	8.2
white bread	1.3	1.9	1.4	1.6	2.7	1.7
porridge	3.2	4.5	9.8	11.2	13.0	8.2
rice, pasta, etc.	4.6	6.4	2.0	2.3	6.6	4.1
**Fats, oils, gravy**	**0.1**	**0.2**	**0.5**	**0.6**	**0.6**	**0.4**
**Fish, fish dishes**	**0.8**	**1.1**	**2.7**	**3.0**	**3.5**	**2.2**
**Eggs, egg dishes**	**0.0**	**0.0**	**0.0**	**0.0**	**0.0**	**0.0**
**Meat, meat dishes**	**4.8**	**6.8**	**8.1**	**9.3**	**13.0**	**8.2**
**Milk, dairy products**	**14.8**	**20.7**	**18.5**	**21.1**	**33.3**	**20.9**
skimmed milk	3.8	5.3	12.8	14.6	16.6	10.4
milk with 1-1.5% fat content	3.8	5.3	2.4	2.7	6.2	3.9
yoghurt and Finnish cultured milk	4.1	5.7	1.0	1.2	5.1	3.2
**Sugar, sweets**	**2.7**	**3.8**	**0.4**	**0.4**	**3.1**	**1.9**
**Drinks**	**3.8**	**5.3**	**1.4**	**1.6**	**5.2**	**3.3**
**Miscellaneous**	**1.8**	**2.5**	**0.6**	**0.7**	**2.3**	**1.5**
**Total**	**71.4**	**100.0**	**87.7**	**100.0**	**159.1**	**100.0**

Table S 8: Sources of carbohydrates (mean and population proportion) in the diet on weekdays among Finnish 5- to 6-year-old preschool children who eat three meals at preschool. Main food groups and selected food groups are presented.

	**Outside preschool**		**Preschool**		**Total**	
	**g/day**	**%**	**g/day**	**%**	**g/day**	**%**
**Vegetables, vegetable dishes**	**3.0**	**3.6**	**4.0**	**4.1**	**7.0**	**3.8**
**Potatoes, potato dishes**	**4.2**	**5.1**	**7.2**	**7.3**	**11.4**	**6.3**
**Fruit, berries, fruit and berry products**	**11.9**	**14.3**	**10.3**	**10.4**	**22.2**	**12.2**
fresh fruit	7.0	8.5	3.2	3.2	10.3	5.6
fruit and berry soups	0.9	1.1	3.4	3.4	4.3	2.4
100% juice	1.2	1.5	0.9	0.9	2.1	1.2
**Cereals, bakery products**	**28.5**	**34.3**	**41.2**	**41.5**	**69.7**	**38.2**
rye bread	3.3	4.0	4.3	4.3	7.6	4.2
rye crispbread	0.5	0.7	5.3	5.3	5.8	3.2
multi-grain bread	4.9	5.9	9.3	9.4	14.2	7.8
white bread	1.7	2.1	2.6	2.6	4.3	2.3
porridge	3.3	3.9	11.8	11.9	15.1	8.3
rice, pasta, etc.	4.8	5.8	2.4	2.4	7.3	4.0
**Fats, oils, gravy**	**0.2**	**0.2**	**0.6**	**0.6**	**0.8**	**0.4**
**Fish, fish dishes**	**1.1**	**1.3**	**2.6**	**2.6**	**3.7**	**2.0**
**Eggs, egg dishes**	**0.0**	**0.0**	**0.0**	**0.0**	**0.0**	**0.0**
**Meat, meat dishes**	**5.5**	**6.6**	**9.5**	**9.6**	**15.0**	**8.2**
**Milk, dairy products**	**16.4**	**19.8**	**20.7**	**20.8**	**37.1**	**20.3**
skimmed milk	4.7	5.7	13.7	13.8	18.4	10.1
milk with 1-1.5% fat content	3.1	3.7	3.0	3.0	6.0	3.3
yoghurt and Finnish cultured milk	4.8	5.7	0.8	0.8	5.6	3.1
**Sugar, sweets**	**5.9**	**7.1**	**1.0**	**1.0**	**6.9**	**3.8**
**Drinks**	**4.4**	**5.3**	**1.4**	**1.4**	**5.8**	**3.2**
**Miscellaneous**	**2.1**	**2.5**	**0.7**	**0.7**	**2.8**	**1.5**
**Total**	**83.2**	**100.0**	**99.3**	**100.0**	**182.4**	**100.0**

Table S 9: Sources of sucrose (mean and population proportion) in the diet on weekdays among Finnish 3- to 4-year-old preschool children who eat three meals at preschool. Main food groups and selected food groups are presented.

	Outside preschool		Preschool		Total	
	g/day	%	g/day	%	g/day	%
**Vegetables, vegetable dishes**	**0.4**	**2.6**	**0.6**	**5.9**	**1.1**	**3.9**
**Potatoes, potato dishes**	**0.0**	**0.0**	**0.0**	**0.1**	**0.0**	**0.1**
**Fruit, berries, fruit and berry products**	**3.5**	**21.9**	**5.0**	**45.7**	**8.5**	**31.6**
fresh fruit	2.2	13.8	0.9	8.6	3.1	11.7
berries	0.1	0.8	0.0	0.1	0.1	0.5
fruit and berry soups	0.2	1.3	2.5	23.2	2.8	10.3
berry and fruit pies	0.0	0.3	0.0	0.0	0.0	0.2
jams, marmalades	0.4	2.7	0.8	7.3	1.2	4.6
100% juice	0.2	1.4	0.2	1.6	0.4	1.5
smoothies	0.1	0.8	0.3	2.5	0.4	1.5
**Cereals, bakery products**	**2.8**	**17.7**	**1.7**	**15.1**	**4.5**	**16.6**
breakfast cereals	0.5	3.4	0.2	1.4	0.7	2.6
buns, sweet bakery	0.8	4.8	0.1	1.0	0.9	3.2
biscuits and mslibars	0.8	5.1	0.2	1.4	1.0	3.6
pancakes, crpes	0.1	0.4	0.1	0.7	0.1	0.6
**Fats, oils, gravy**	**0.0**	**0.1**	**0.1**	**0.6**	**0.1**	**0.3**
**Fish, fish dishes**	**0.0**	**0.1**	**0.0**	**0.3**	**0.1**	**0.2**
**Eggs, egg dishes**	**0.0**	**0.0**	**0.0**	**0.0**	**0.0**	**0.0**
**Meat, meat dishes**	**0.2**	**1.3**	**0.3**	**3.0**	**0.5**	**2.0**
**Milk, dairy products**	**3.9**	**24.4**	**1.7**	**16.0**	**5.6**	**21.0**
sweetened milk drinks (cocoa etc.)	0.4	2.5	0.1	0.8	0.5	1.8
yoghurt and Finnish cultured milk	2.4	15.2	0.6	5.2	3.0	11.1
dairy-based desserts	0.5	3.0	1.0	8.9	1.5	5.4
ice-cream	0.6	3.5	0.1	0.9	0.7	2.5
**Sugar, sweets**	**1.5**	**9.4**	**0.3**	**2.6**	**1.8**	**6.6**
sugar, honey	0.2	1.1	0.2	1.7	0.4	1.4
sweets	0.6	3.9	0.1	0.6	0.7	2.6
chocolate	0.7	4.4	0.0	0.3	0.7	2.7
**Drinks**	**3.1**	**19.4**	**1.0**	**8.8**	**4.0**	**15.1**
sugar-sweetened juice	2.1	13.5	0.5	4.6	2.6	9.9
sugar-sweetened soda	0.3	2.1		0.0	0.3	1.2
non-dairy milk substitutes	0.4	2.2	0.4	4.1	0.8	3.0
**Miscellaneous**	**0.5**	**3.1**	**0.2**	**2.0**	**0.7**	**2.7**
**Total**	**15.9**	**100.0**	**10.9**	**100.0**	**26.8**	**100.0**

Table S 10: Sources of sucrose (mean and population proportion) in the diet on weekdays among Finnish 5- to 6-year-old preschool children who eat three meals at preschool. Main food groups and selected food groups are presented.

	**Outside preschool**		**Preschool**		**Total**	
	**g/day**	**%**	**g/day**	**%**	**g/day**	**%**
**Vegetables, vegetable dishes**	**0.4**	**2.3**	**0.8**	**6.5**	**1.3**	**3.9**
**Potatoes, potato dishes**	**0.0**	**0.0**	**0.0**	**0.1**	**0.0**	**0.1**
**Fruit, berries, fruit and berry products**	**4.3**	**22.0**	**5.2**	**40.7**	**9.4**	**29.3**
fresh fruit	2.2	11.5	1.0	7.9	3.2	10.1
berries	0.1	0.5	0.0	0.1	0.1	0.4
fruit and berry soups	0.5	2.4	2.2	17.1	2.6	8.2
berry and fruit pies	0.2	0.9	0.0	0.1	0.2	0.6
jams, marmalades	0.5	2.6	1.2	9.6	1.7	5.3
100% juice	0.3	1.8	0.2	1.9	0.6	1.8
smoothies	0.2	1.1	0.2	1.4	0.4	1.2
**Cereals, bakery products**	**2.6**	**13.6**	**2.2**	**17.2**	**4.8**	**15.0**
breakfast cereals	0.8	3.9	0.2	1.3	0.9	2.9
buns, sweet bakery	0.5	2.8	0.2	1.5	0.7	2.3
biscuits and mslibars	0.7	3.6	0.3	2.7	1.0	3.2
pancakes, crpes	0.0	0.2	0.1	0.8	0.1	0.4
**Fats, oils, gravy**	**0.0**	**0.1**	**0.1**	**0.6**	**0.1**	**0.3**
**Fish, fish dishes**	**0.0**	**0.2**	**0.0**	**0.3**	**0.1**	**0.2**
**Eggs, egg dishes**	**0.0**	**0.0**	**0.0**	**0.0**	**0.0**	**0.0**
**Meat, meat dishes**	**0.2**	**1.1**	**0.4**	**3.0**	**0.6**	**1.8**
**Milk, dairy products**	**4.5**	**23.2**	**2.0**	**16.1**	**6.6**	**20.4**
sweetened milk drinks (cocoa etc.)	0.3	1.8	0.2	1.2	0.5	1.6
yoghurt and Finnish cultured milk	2.8	14.2	0.4	2.8	3.1	9.7
dairy-based desserts	0.7	3.8	1.3	10.3	2.0	6.4
ice-cream	0.6	3.3	0.2	1.6	0.9	2.6
**Sugar, sweets**	**3.1**	**15.8**	**0.7**	**5.6**	**3.8**	**11.8**
sugar, honey	0.2	1.2	0.4	2.9	0.6	1.9
sweets	1.9	9.8	0.2	1.6	2.1	6.6
chocolate	0.9	4.7	0.1	1.1	1.1	3.3
**Drinks**	**3.8**	**19.7**	**1.1**	**8.5**	**4.9**	**15.3**
sugar-sweetened juice	2.8	14.3	0.9	7.1	3.7	11.5
sugar-sweetened soda	0.8	4.0		0.0	0.8	2.5
non-dairy milk substitutes	0.2	1.3	0.2	1.4	0.4	1.3
**Miscellaneous**	**0.4**	**2.2**	**0.2**	**1.5**	**0.6**	**1.9**
**Total**	**19.5**	**100.0**	**12.7**	**100.0**	**32.1**	**100.0**

Table S 11: Sources of added sugar (mean and population proportion) in the diet on weekdays among Finnish 3- to 4-year-old preschool children who eat three meals at preschool. Main food groups and selected food groups are presented.

	Outside preschool		Preschool		Total	
	g/day	%	g/day	%	g/day	%
**Vegetables, vegetable dishes**	**0.0**	**0.0**	**0.0**	**0.0**	**0.0**	**0.0**
**Potatoes, potato dishes**	**0.0**	**0.0**	**0.0**	**0.0**	**0.0**	**0.0**
**Fruit, berries, fruit and berry products**	**0.8**	**6.4**	**3.9**	**47.7**	**4.7**	**22.5**
fruit and berry soups	0.2	1.8	2.8	34.4	3.0	14.5
berry and fruit pies	0.0	0.4	0.0	0.0	0.1	0.2
jams, marmalades	0.4	3.3	0.9	10.5	1.3	6.1
**Cereals, bakery products**	**2.7**	**21.6**	**1.4**	**17.5**	**4.2**	**20.0**
breakfast cereals	0.6	4.5	0.1	1.4	0.7	3.3
buns, sweet bakery	0.8	6.0	0.1	1.3	0.9	4.2
biscuits and mslibars	0.8	6.4	0.2	1.9	1.0	4.6
pancakes, crpes	0.1	0.5	0.1	1.0	0.1	0.7
**Fats, oils, gravy**	**0.0**	**0.1**	**0.1**	**0.8**	**0.1**	**0.3**
**Fish, fish dishes**	**0.0**	**0.0**	**0.0**	**0.0**	**0.0**	**0.0**
**Eggs, egg dishes**	**0.0**	**0.0**	**0.0**	**0.0**	**0.0**	**0.0**
**Meat, meat dishes**	**0.0**	**0.0**	**0.0**	**0.0**	**0.0**	**0.0**
**Milk, dairy products**	**3.9**	**30.6**	**1.7**	**20.9**	**5.6**	**26.8**
sweetened milk drinks (cocoa etc.)	0.4	3.1	0.1	1.1	0.5	2.3
yoghurt and Finnish cultured milk	2.4	19.0	0.5	5.8	2.9	13.9
dairy-based desserts	0.5	4.0	1.0	12.8	1.5	7.4
ice-cream	0.6	4.4	0.1	1.2	0.7	3.2
**Sugar, sweets**	**2.1**	**16.4**	**0.3**	**4.0**	**2.4**	**11.5**
sugar, honey	0.4	3.5	0.2	2.5	0.6	3.1
sweets	0.9	7.0	0.1	1.2	1.0	4.7
chocolate	0.7	5.9	0.0	0.3	0.8	3.7
**Drinks**	**2.7**	**21.4**	**0.5**	**6.4**	**3.2**	**15.6**
sugar-sweetened juice	2.2	17.5	0.5	6.0	2.7	13.0
sugar-sweetened soda	0.4	2.8	0.0	0.0	0.4	1.7
non-dairy milk substitutes	0.0	0.2	0.0	0.4	0.1	0.3
**Miscellaneous**	**0.5**	**3.6**	**0.2**	**2.7**	**0.7**	**3.2**
**Total**	**12.7**	**100.0**	**8.1**	**100.0**	**20.8**	**100.0**

Table S 12: Sources of added sugar (mean and population proportion) in the diet on weekdays among Finnish 5- to 6-year-old preschool children who eat three meals at preschool. Main food groups and selected food groups are presented.

	Outside preschool		Preschool		Total	
	g/day	%	g/day	%	g/day	%
**Vegetables, vegetable dishes**	**0.0**	**0.0**	**0.0**	**0.0**	**0.0**	**0.0**
**Potatoes, potato dishes**	**0.0**	**0.0**	**0.0**	**0.0**	**0.0**	**0.0**
**Fruit, berries, fruit and berry products**	**1.3**	**8.0**	**3.8**	**39.2**	**5.2**	**19.5**
fruit and berry soups	0.5	3.1	2.3	23.8	2.9	10.7
berry and fruit pies	0.2	1.1	0.0	0.2	0.2	0.7
jams, marmalades	0.5	3.0	1.2	12.6	1.7	6.6
**Cereals, bakery products**	**2.5**	**14.8**	**1.9**	**19.6**	**4.4**	**16.6**
breakfast cereals	0.7	4.4	0.1	1.2	0.9	3.3
buns, sweet bakery	0.5	3.3	0.2	2.0	0.7	2.8
biscuits and mslibars	0.7	4.2	0.3	3.4	1.0	3.9
pancakes, crpes	0.0	0.2	0.1	1.1	0.1	0.5
**Fats, oils, gravy**	**0.0**	**0.1**	**0.1**	**0.7**	**0.1**	**0.3**
**Fish, fish dishes**	**0.0**	**0.0**	**0.0**	**0.0**	**0.0**	**0.0**
**Eggs, egg dishes**	**0.0**	**0.0**	**0.0**	**0.0**	**0.0**	**0.0**
**Meat, meat dishes**	**0.0**	**0.0**	**0.0**	**0.0**	**0.0**	**0.0**
**Milk, dairy products**	**4.5**	**27.1**	**2.1**	**21.3**	**6.6**	**24.9**
sweetened milk drinks (cocoa etc.)	0.3	2.1	0.2	1.6	0.5	1.9
yoghurt and Finnish cultured milk	2.8	16.5	0.4	3.6	3.1	11.8
dairy-based desserts	0.8	4.6	1.4	13.9	2.1	8.1
ice-cream	0.7	3.9	0.2	2.1	0.9	3.2
**Sugar, sweets**	**4.3**	**25.8**	**0.8**	**8.4**	**5.2**	**19.4**
sugar, honey	0.5	3.1	0.4	3.9	0.9	3.4
sweets	2.8	16.8	0.3	3.0	3.1	11.7
chocolate	1.0	5.9	0.1	1.5	1.1	4.3
**Drinks**	**3.7**	**21.9**	**0.9**	**9.0**	**4.6**	**17.2**
sugar-sweetened juice	2.8	17.0	0.9	8.9	3.7	14.0
sugar-sweetened soda	0.8	4.9	0.0	0.0	0.8	3.1
non-dairy milk substitutes	0.0	0.1	0.0	0.1	0.0	0.1
**Miscellaneous**	**0.4**	**2.2**	**0.2**	**1.8**	**0.6**	**2.1**
**Total**	**16.8**	**100.0**	**9.8**	**100.0**	**26.6**	**100.0**

Table S 13: Sources of fiber (mean and population proportion) in the diet on weekdays among Finnish 3- to 4-year-old preschool children who eat three meals at preschool. Main food groups and selected food groups are presented.

	Outside preschool		Preschool		Total	
	g/day	%	g/day	%	g/day	%
**Vegetables, vegetable dishes**	**0.8**	**13.2**	**0.8**	**8.6**	**1.6**	**10.5**
**Potatoes, potato dishes**	**0.2**	**3.0**	**0.4**	**4.6**	**0.6**	**4.0**
**Fruit, berries, fruit and berry products**	**1.5**	**24.7**	**0.9**	**10.0**	**2.4**	**15.9**
fresh fruit	1.2	20.3	0.5	5.8	1.8	11.6
fruit and berry soups	0.0	0.4	0.2	1.9	0.2	1.3
**Cereals, bakery products**	**2.6**	**42.7**	**5.9**	**64.5**	**8.5**	**55.8**
rye bread	0.6	10.1	1.0	11.1	1.6	10.7
rye crispbread	0.2	3.9	1.9	20.9	2.2	14.1
multi-grain bread	0.5	7.8	1.0	11.4	1.5	9.9
white bread	0.1	1.3	0.1	0.8	0.2	1.0
porridge	0.4	5.8	1.5	16.0	1.8	11.9
rice, pasta, etc.	0.2	4.1	0.2	1.8	0.4	2.7
**Fats, oils, gravy**	**0.0**	**0.0**	**0.0**	**0.1**	**0.0**	**0.1**
**Fish, fish dishes**	**0.1**	**1.0**	**0.2**	**2.1**	**0.3**	**1.7**
**Eggs, egg dishes**	**0.0**	**0.0**	**0.0**	**0.0**	**0.0**	**0.0**
**Meat, meat dishes**	**0.4**	**6.9**	**0.7**	**7.4**	**1.1**	**7.2**
**Milk, dairy products**	**0.1**	**1.8**	**0.1**	**1.2**	**0.2**	**1.4**
**Sugar, sweets**	**0.0**	**0.6**	**0.0**	**0.0**	**0.0**	**0.2**
**Drinks**	**0.1**	**2.1**	**0.1**	**0.8**	**0.2**	**1.3**
**Miscellaneous**	**0.2**	**3.9**	**0.1**	**0.6**	**0.3**	**1.9**
**Total**	**6.1**	**100.0**	**9.1**	**100.0**	**15.3**	**100.0**

Table S 14: Sources of fiber (mean and population proportion) in the diet on weekdays among Finnish 5- to 6-year-old preschool children who eat three meals at preschool. Main food groups and selected food groups are presented.

	Outside preschool		Preschool		Total	
	g/day	%	g/day	%	g/day	%
**Vegetables, vegetable dishes**	**0.8**	**11.6**	**1.0**	**9.6**	**1.7**	**10.4**
**Potatoes, potato dishes**	**0.2**	**3.1**	**0.5**	**5.0**	**0.7**	**4.3**
**Fruit, berries, fruit and berry products**	**1.7**	**25.7**	**0.9**	**9.2**	**2.6**	**15.7**
fresh fruit	1.2	18.0	0.5	5.4	1.7	10.4
fruit and berry soups	0.1	1.0	0.1	1.3	0.2	1.2
**Cereals, bakery products**	**2.8**	**42.8**	**6.4**	**63.4**	**9.2**	**55.3**
rye bread	0.7	10.4	0.9	9.1	1.6	9.6
rye crispbread	0.2	2.4	2.1	20.9	2.3	13.6
multi-grain bread	0.6	8.7	1.1	10.7	1.7	9.9
white bread	0.1	1.7	0.1	1.3	0.2	1.5
porridge	0.4	5.8	1.6	16.2	2.0	12.1
rice, pasta, etc.	0.3	4.6	0.2	2.1	0.5	3.1
**Fats, oils, gravy**	**0.0**	**0.1**	**0.0**	**0.1**	**0.0**	**0.1**
**Fish, fish dishes**	**0.1**	**1.2**	**0.2**	**1.9**	**0.3**	**1.6**
**Eggs, egg dishes**	**0.0**	**0.0**	**0.0**	**0.0**	**0.0**	**0.0**
**Meat, meat dishes**	**0.5**	**7.3**	**0.8**	**8.4**	**1.3**	**7.9**
**Milk, dairy products**	**0.1**	**2.1**	**0.1**	**1.2**	**0.3**	**1.6**
**Sugar, sweets**	**0.0**	**0.5**	**0.0**	**0.0**	**0.0**	**0.2**
**Drinks**	**0.1**	**1.3**	**0.0**	**0.5**	**0.1**	**0.8**
**Miscellaneous**	**0.3**	**4.2**	**0.1**	**0.6**	**0.3**	**2.0**
**Total**	**6.6**	**100.0**	**10.1**	**100.0**	**16.7**	**100.0**

Table S 15: Sources of fat (mean and population proportion) in the diet on weekdays among Finnish 3- to 4-year-old preschool children who eat three meals at preschool. Main food groups and selected food groups are presented.

	**Outside preschool**		**Preschool**		**Total**	
	**g/day**	**%**	**g/day**	**%**	**g/day**	**%**
**Vegetables, vegetable dishes**	**0.9**	**3.8**	**0.8**	**3.0**	**1.6**	**3.4**
**Potatoes, potato dishes**	**0.4**	**1.9**	**0.3**	**1.2**	**0.7**	**1.5**
**Fruit, berries, fruit and berry products**	**0.3**	**1.3**	**0.3**	**1.0**	**0.5**	**1.1**
**Cereals, bakery products**	**3.5**	**15.5**	**2.9**	**11.7**	**6.4**	**13.5**
**Fats, oils, gravy**	**3.5**	**15.8**	**10.1**	**40.2**	**13.6**	**28.7**
margarine and fat spread	1.4	6.1	9.8	39.1	11.2	23.6
blended spread	1.7	7.6	0.0	0.0	1.7	3.6
butter	0.2	0.8	0.0	0.0	0.2	0.4
**Fish, fish dishes**	**1.0**	**4.3**	**1.7**	**6.9**	**2.7**	**5.7**
**Eggs, egg dishes**	**0.3**	**1.4**	**0.2**	**1.0**	**0.5**	**1.1**
**Meat, meat dishes**	**6.4**	**28.8**	**5.8**	**23.1**	**12.2**	**25.8**
cold cuts	0.2	1.0	0.1	0.5	0.4	0.7
red meat dishes	4.0	17.7	2.8	11.2	6.8	14.3
poultry dishes	1.3	6.0	1.3	5.1	2.6	5.5
sausage dishes	0.8	3.8	1.6	6.2	2.4	5.0
**Milk, dairy products**	**4.8**	**21.4**	**2.7**	**10.6**	**7.4**	**15.7**
milk with 1-1.5% fat content	1.3	5.6	0.5	2.1	1.8	3.7
whole milk	0.2	0.8	0.0	0.0	0.2	0.4
yoghurt and Finnish cultured milk	0.9	3.9	0.2	0.8	1.1	2.2
cheese	1.4	6.5	0.7	2.9	2.2	4.6
dairy-based desserts	0.3	1.3	0.7	2.9	1.0	2.2
**Sugar, sweets**	**0.5**	**2.2**	**0.0**	**0.1**	**0.5**	**1.1**
**Drinks**	**0.2**	**0.8**	**0.1**	**0.6**	**0.3**	**0.7**
**Miscellaneous**	**0.6**	**2.9**	**0.1**	**0.5**	**0.8**	**1.6**
**Total**	**22.3**	**100.0**	**25.1**	**100.0**	**47.4**	**100.0**

Table S 16: Sources of fat (mean and population proportion) in the diet on weekdays among Finnish 5- to 6-year-old preschool children who eat three meals at preschool. Main food groups and selected food groups are presented.

	Outside preschool		Preschool		Total	
	g/day	%	g/day	%	g/day	%
**Vegetables, vegetable dishes**	**0.7**	**2.7**	**0.9**	**3.2**	**1.6**	**3.0**
**Potatoes, potato dishes**	**0.7**	**2.8**	**0.4**	**1.5**	**1.1**	**2.1**
**Fruit, berries, fruit and berry products**	**0.4**	**1.7**	**0.2**	**0.8**	**0.7**	**1.2**
**Cereals, bakery products**	**3.6**	**14.3**	**3.8**	**12.9**	**7.3**	**13.6**
**Fats, oils, gravy**	**3.6**	**14.3**	**11.5**	**39.5**	**15.0**	**27.9**
margarine and fat spread	1.3	5.3	11.1	38.4	12.5	23.1
blended spread	1.6	6.4	0.0	0.1	1.6	3.0
butter	0.2	0.8	0.0	0.0	0.2	0.4
**Fish, fish dishes**	**1.1**	**4.4**	**1.9**	**6.5**	**3.0**	**5.5**
**Eggs, egg dishes**	**0.3**	**1.2**	**0.2**	**0.7**	**0.5**	**0.9**
**Meat, meat dishes**	**7.6**	**30.4**	**6.9**	**23.6**	**14.4**	**26.7**
cold cuts	0.3	1.1	0.2	0.6	0.4	0.8
red meat dishes	4.7	18.9	3.5	12.2	8.3	15.3
poultry dishes	1.4	5.6	1.8	6.1	3.2	5.9
sausage dishes	1.2	4.6	1.3	4.3	2.4	4.5
**Milk, dairy products**	**5.2**	**20.8**	**2.9**	**10.0**	**8.1**	**15.0**
milk with 1-1.5% fat content	1.0	4.0	0.7	2.3	1.7	3.1
whole milk	0.2	0.8	0.0	0.0	0.2	0.4
yoghurt and Finnish cultured milk	1.0	4.1	0.2	0.6	1.2	2.2
cheese	1.6	6.4	0.7	2.6	2.3	4.3
dairy-based desserts	0.3	1.3	0.8	2.7	1.1	2.1
**Sugar, sweets**	**0.8**	**3.1**	**0.1**	**0.3**	**0.9**	**1.6**
**Drinks**	**0.1**	**0.4**	**0.1**	**0.3**	**0.2**	**0.3**
**Miscellaneous**	**0.9**	**3.8**	**0.2**	**0.7**	**1.1**	**2.1**
**Total**	**25.0**	**100.0**	**29.0**	**100.0**	**54.0**	**100.0**

Table S 17: Sources of saturated fatty acids (mean and population proportion) in the diet on weekdays among Finnish 3- to 4-year-old preschool children who eat three meals at preschool. Main food groups and selected food groups are presented.

	**Outside preschool**		**Preschool**		**Total**	
	**g/day**	**%**	**g/day**	**%**	**g/day**	**%**
**Vegetables, vegetable dishes**	**0.2**	**2.5**	**0.2**	**2.5**	**0.4**	**2.5**
**Potatoes, potato dishes**	**0.2**	**1.9**	**0.1**	**1.5**	**0.3**	**1.7**
**Fruit, berries, fruit and berry products**	**0.1**	**0.6**	**0.1**	**1.1**	**0.1**	**0.8**
**Cereals, bakery products**	**1.1**	**12.8**	**0.7**	**8.6**	**1.9**	**10.8**
**Fats, oils, gravy**	**1.3**	**14.6**	**2.9**	**35.0**	**4.2**	**24.5**
margarine and fat spread	0.4	4.5	2.9	34.4	3.3	19.1
blended spread	0.7	7.9	0.0	0.0	0.7	4.0
butter	0.1	1.4	0.0	0.0	0.1	0.7
**Fish, fish dishes**	**0.2**	**2.3**	**0.4**	**5.0**	**0.6**	**3.6**
**Eggs, egg dishes**	**0.1**	**0.8**	**0.0**	**0.6**	**0.1**	**0.7**
**Meat, meat dishes**	**2.2**	**24.4**	**2.0**	**24.0**	**4.2**	**24.2**
cold cuts	0.1	0.9	0.0	0.5	0.1	0.7
red meat dishes	1.4	16.1	1.0	12.0	2.4	14.1
poultry dishes	0.3	3.7	0.4	4.5	0.7	4.0
sausage dishes	0.3	3.6	0.6	6.9	0.9	5.2
**Milk, dairy products**	**3.1**	**35.2**	**1.8**	**20.9**	**4.9**	**28.3**
milk with 1-1.5% fat content	0.9	9.9	0.4	4.3	1.2	7.2
whole milk	0.1	1.3	0.0	0.0	0.1	0.7
yoghurt and Finnish cultured milk	0.6	6.3	0.1	1.4	0.7	3.9
cheese	0.9	10.5	0.5	5.6	1.4	8.1
dairy-based desserts	0.2	2.1	0.5	5.7	0.7	3.8
**Sugar, sweets**	**0.3**	**3.1**	**0.0**	**0.2**	**0.3**	**1.7**
**Drinks**	**0.0**	**0.3**	**0.0**	**0.3**	**0.0**	**0.3**
**Miscellaneous**	**0.2**	**1.7**	**0.0**	**0.3**	**0.2**	**1.0**
**Total**	**8.9**	**100.0**	**8.4**	**100.0**	**17.3**	**100.0**

Table S 18: Sources of saturated fatty acids (mean and population proportion) in the diet on weekdays among Finnish 5- to 6-year-old preschool children who eat three meals at preschool. Main food groups and selected food groups are presented.

	**Outside preschool**		**Preschool**		**Total**	
	**g/day**	**%**	**g/day**	**%**	**g/day**	**%**
**Vegetables, vegetable dishes**	**0.2**	**2.2**	**0.3**	**2.9**	**0.5**	**2.6**
**Potatoes, potato dishes**	**0.2**	**2.4**	**0.2**	**1.9**	**0.4**	**2.2**
**Fruit, berries, fruit and berry products**	**0.1**	**1.0**	**0.1**	**0.8**	**0.2**	**0.9**
**Cereals, bakery products**	**1.1**	**11.6**	**1.1**	**10.8**	**2.2**	**11.2**
**Fats, oils, gravy**	**1.2**	**12.8**	**3.4**	**34.4**	**4.6**	**23.6**
margarine and fat spread	0.4	3.8	3.3	33.7	3.7	18.7
blended spread	0.7	6.7	0.0	0.1	0.7	3.4
butter	0.1	1.3	0.0	0.0	0.1	0.7
**Fish, fish dishes**	**0.3**	**2.7**	**0.4**	**4.4**	**0.7**	**3.5**
**Eggs, egg dishes**	**0.1**	**0.6**	**0.0**	**0.4**	**0.1**	**0.5**
**Meat, meat dishes**	**2.5**	**25.9**	**2.3**	**23.9**	**4.9**	**24.9**
cold cuts	0.1	1.0	0.1	0.6	0.2	0.8
red meat dishes	1.7	17.0	1.3	13.0	2.9	15.0
poultry dishes	0.3	3.3	0.5	5.2	0.8	4.2
sausage dishes	0.4	4.4	0.5	4.7	0.9	4.6
**Milk, dairy products**	**3.4**	**34.7**	**1.9**	**19.6**	**5.3**	**27.1**
milk with 1-1.5% fat content	0.7	7.2	0.5	4.8	1.2	6.0
whole milk	0.1	1.4	0.0	0.0	0.1	0.7
yoghurt and Finnish cultured milk	0.7	6.9	0.1	1.1	0.8	4.0
cheese	1.0	10.7	0.5	4.8	1.5	7.7
dairy-based desserts	0.2	1.8	0.5	5.2	0.7	3.5
**Sugar, sweets**	**0.4**	**4.5**	**0.1**	**0.5**	**0.5**	**2.5**
**Drinks**	**0.0**	**0.1**	**0.0**	**0.1**	**0.0**	**0.1**
**Miscellaneous**	**0.2**	**1.6**	**0.0**	**0.4**	**0.2**	**1.0**
**Total**	**9.8**	**100.0**	**9.8**	**100.0**	**19.5**	**100.0**

Table S 19: Sources of monounsaturated fatty acids (mean and population proportion) in the diet on weekdays among Finnish 3- to 4-year-old preschool children who eat three meals at preschool. Main food groups and selected food groups are presented.

	Outside preschool		Preschool		Total	
	g/day	%	g/day	%	g/day	%
**Vegetables, vegetable dishes**	**0.3**	**4.6**	**0.3**	**3.0**	**0.6**	**3.7**
**Potatoes, potato dishes**	**0.2**	**2.0**	**0.1**	**0.7**	**0.2**	**1.3**
**Fruit, berries, fruit and berry products**	**0.0**	**0.7**	**0.0**	**0.5**	**0.1**	**0.5**
**Cereals, bakery products**	**1.1**	**15.2**	**0.8**	**8.7**	**1.9**	**11.6**
**Fats, oils, gravy**	**1.3**	**17.3**	**4.2**	**46.3**	**5.5**	**33.3**
margarine and fat spread	0.6	7.4	4.1	44.7	4.6	27.9
blended spread	0.5	7.4	0.0	0.0	0.6	3.3
butter	0.0	0.6	0.0	0.0	0.0	0.3
**Fish, fish dishes**	**0.4**	**4.8**	**0.7**	**7.3**	**1.0**	**6.2**
**Eggs, egg dishes**	**0.1**	**1.4**	**0.1**	**0.8**	**0.2**	**1.1**
**Meat, meat dishes**	**2.5**	**33.3**	**2.3**	**24.8**	**4.7**	**28.6**
cold cuts	0.1	1.1	0.1	0.6	0.1	0.8
red meat dishes	1.5	19.8	1.1	11.9	2.6	15.5
poultry dishes	0.5	7.3	0.5	5.3	1.0	6.2
sausage dishes	0.3	4.7	0.6	7.0	1.0	6.0
**Milk, dairy products**	**1.1**	**14.9**	**0.6**	**6.7**	**1.7**	**10.4**
milk with 1-1.5% fat content	0.3	4.1	0.1	1.3	0.4	2.6
whole milk	0.0	0.5	0.0	0.0	0.0	0.2
yoghurt and Finnish cultured milk	0.2	2.5	0.0	0.4	0.2	1.4
cheese	0.3	4.5	0.2	1.8	0.5	3.1
dairy-based desserts	0.1	1.0	0.2	1.8	0.2	1.5
**Sugar, sweets**	**0.1**	**1.9**	**0.0**	**0.1**	**0.2**	**0.9**
**Drinks**	**0.1**	**0.8**	**0.1**	**0.7**	**0.1**	**0.7**
**Miscellaneous**	**0.2**	**3.2**	**0.0**	**0.5**	**0.3**	**1.7**
**Total**	**7.4**	**100.0**	**9.1**	**100.0**	**16.5**	**100.0**

Table S 20: Sources of monounsaturated fatty acids (mean and population proportion) in the diet on weekdays among Finnish 5- to 6-year-old preschool children who eat three meals at preschool. Main food groups and selected food groups are presented.

	Outside preschool		Preschool		Total	
	g/day	%	g/day	%	g/day	%
**Vegetables, vegetable dishes**	**0.2**	**2.4**	**0.3**	**2.9**	**0.5**	**2.7**
**Potatoes, potato dishes**	**0.3**	**3.3**	**0.1**	**1.0**	**0.4**	**2.0**
**Fruit, berries, fruit and berry products**	**0.1**	**1.0**	**0.0**	**0.3**	**0.1**	**0.6**
**Cereals, bakery products**	**1.2**	**13.8**	**1.0**	**10.0**	**2.2**	**11.7**
**Fats, oils, gravy**	**1.3**	**15.7**	**4.8**	**45.7**	**6.1**	**32.4**
margarine and fat spread	0.5	6.3	4.6	44.1	5.2	27.3
blended spread	0.5	6.2	0.0	0.1	0.5	2.8
butter	0.0	0.6	0.0	0.0	0.0	0.2
**Fish, fish dishes**	**0.4**	**5.0**	**0.7**	**6.7**	**1.1**	**5.9**
**Eggs, egg dishes**	**0.1**	**1.3**	**0.1**	**0.6**	**0.2**	**0.9**
**Meat, meat dishes**	**3.0**	**35.5**	**2.6**	**25.1**	**5.6**	**29.7**
cold cuts	0.1	1.3	0.1	0.7	0.2	1.0
red meat dishes	1.8	21.4	1.3	12.9	3.1	16.6
poultry dishes	0.6	6.9	0.6	6.2	1.2	6.5
sausage dishes	0.5	5.7	0.5	5.0	1.0	5.3
**Milk, dairy products**	**1.2**	**14.5**	**0.7**	**6.4**	**1.9**	**10.0**
milk with 1-1.5% fat content	0.2	2.9	0.2	1.5	0.4	2.1
whole milk	0.0	0.5	0.0	0.0	0.0	0.2
yoghurt and Finnish cultured milk	0.2	2.7	0.0	0.3	0.3	1.4
cheese	0.4	4.5	0.2	1.7	0.6	2.9
dairy-based desserts	0.1	1.0	0.2	1.7	0.3	1.4
**Sugar, sweets**	**0.2**	**2.7**	**0.0**	**0.2**	**0.2**	**1.3**
**Drinks**	**0.0**	**0.5**	**0.0**	**0.2**	**0.1**	**0.3**
**Miscellaneous**	**0.4**	**4.4**	**0.1**	**0.8**	**0.5**	**2.4**
**Total**	**8.4**	**100.0**	**10.5**	**100.0**	**18.8**	**100.0**

Table S 21: Sources of polyunsaturated fatty acids (mean and population proportion) in the diet on weekdays among Finnish 3- to 4-year-old preschool children who eat three meals at preschool. Main food groups and selected food groups are presented.

	**Outside preschool**		**Preschool**		**Total**	
	**g/day**	**%**	**g/day**	**%**	**g/day**	**%**
**Vegetables, vegetable dishes**	**0.2**	**5.6**	**0.2**	**3.6**	**0.4**	**4.4**
**Potatoes, potato dishes**	**0.1**	**1.9**	**0.0**	**0.7**	**0.1**	**1.2**
**Fruit, berries, fruit and berry products**	**0.1**	**1.8**	**0.0**	**0.7**	**0.1**	**1.1**
**Cereals, bakery products**	**0.7**	**21.6**	**0.7**	**14.1**	**1.4**	**17.0**
**Fats, oils, gravy**	**0.6**	**19.6**	**2.5**	**50.7**	**3.1**	**38.7**
margarine and fat spread	0.4	11.3	2.4	49.3	2.8	34.6
blended spread	0.2	6.4	0.0	0.0	0.2	2.5
butter	0.0	0.2	0.0	0.0	0.0	0.1
**Fish, fish dishes**	**0.3**	**8.7**	**0.5**	**9.4**	**0.7**	**9.1**
**Eggs, egg dishes**	**0.0**	**1.3**	**0.0**	**0.6**	**0.1**	**0.8**
**Meat, meat dishes**	**0.9**	**27.7**	**0.9**	**17.4**	**1.7**	**21.4**
cold cuts	0.0	0.9	0.0	0.4	0.0	0.6
red meat dishes	0.4	13.3	0.4	7.5	0.8	9.7
poultry dishes	0.3	9.4	0.2	5.0	0.5	6.7
sausage dishes	0.1	3.9	0.2	4.5	0.3	4.2
**Milk, dairy products**	**0.1**	**4.1**	**0.1**	**1.2**	**0.2**	**2.3**
milk with 1-1.5% fat content	0.0	0.8	0.0	0.2	0.0	0.4
whole milk	0.0	0.1	0.0	0.0	0.0	0.0
yoghurt and Finnish cultured milk	0.0	0.7	0.0	0.1	0.0	0.3
cheese	0.0	1.3	0.0	0.4	0.1	0.7
dairy-based desserts	0.0	0.5	0.0	0.4	0.0	0.4
**Sugar, sweets**	**0.0**	**0.5**	**0.0**	**0.0**	**0.0**	**0.2**
**Drinks**	**0.1**	**2.0**	**0.1**	**1.1**	**0.1**	**1.4**
**Miscellaneous**	**0.2**	**5.3**	**0.0**	**0.5**	**0.2**	**2.4**
**Total**	**3.1**	**100.0**	**4.9**	**100.0**	**8.0**	**100.0**

Table S 22: Sources of polyunsaturated fatty acids (mean and population proportion) in the diet on weekdays among Finnish 5- to 6-year-old preschool children who eat three meals at preschool. Main food groups and selected food groups are presented.

	Outside preschool		Preschool		Total	
	g/day	%	g/day	%	g/day	%
**Vegetables, vegetable dishes**	**0.2**	**4.4**	**0.2**	**3.5**	**0.4**	**3.9**
**Potatoes, potato dishes**	**0.1**	**3.3**	**0.1**	**0.9**	**0.2**	**1.8**
**Fruit, berries, fruit and berry products**	**0.1**	**2.3**	**0.0**	**0.6**	**0.1**	**1.2**
**Cereals, bakery products**	**0.7**	**20.3**	**0.8**	**14.4**	**1.6**	**16.7**
**Fats, oils, gravy**	**0.7**	**18.8**	**2.8**	**49.1**	**3.5**	**37.4**
margarine and fat spread	0.4	10.1	2.7	47.7	3.1	33.2
blended spread	0.2	5.2	0.0	0.0	0.2	2.0
butter	0.0	0.2	0.0	0.0	0.0	0.1
**Fish, fish dishes**	**0.3**	**8.0**	**0.6**	**10.1**	**0.9**	**9.3**
**Eggs, egg dishes**	**0.0**	**1.2**	**0.0**	**0.4**	**0.1**	**0.7**
**Meat, meat dishes**	**1.0**	**28.5**	**1.1**	**18.5**	**2.1**	**22.4**
cold cuts	0.0	1.0	0.0	0.4	0.1	0.7
red meat dishes	0.5	13.6	0.5	8.2	1.0	10.3
poultry dishes	0.3	9.2	0.4	6.2	0.7	7.4
sausage dishes	0.2	4.5	0.2	3.3	0.4	3.8
**Milk, dairy products**	**0.1**	**3.9**	**0.1**	**1.1**	**0.2**	**2.2**
milk with 1-1.5% fat content	0.0	0.5	0.0	0.2	0.0	0.4
whole milk	0.0	0.1	0.0	0.0	0.0	0.0
yoghurt and Finnish cultured milk	0.0	0.7	0.0	0.1	0.0	0.3
cheese	0.0	1.2	0.0	0.3	0.1	0.7
dairy-based desserts	0.0	0.6	0.0	0.4	0.0	0.5
**Sugar, sweets**	**0.0**	**0.6**	**0.0**	**0.0**	**0.0**	**0.3**
**Drinks**	**0.0**	**1.0**	**0.0**	**0.4**	**0.1**	**0.7**
**Miscellaneous**	**0.3**	**7.6**	**0.1**	**0.9**	**0.3**	**3.5**
**Total**	**3.6**	**100.0**	**5.7**	**100.0**	**9.4**	**100.0**

Table S 23: Sources of vitamin A (retinol activity equivalents) (mean and population proportion) in the diet on weekdays among Finnish 3- to 4-year-old preschool children who eat three meals at preschool. Main food groups and selected food groups are presented.

	Outside preschool		Preschool		Total	
	μg/day	%	μg/day	%	μg/day	%
**Vegetables, vegetable dishes**	**84**	**28**	**94**	**30**	**178**	**29**
fresh vegetables and vegetable salads	66	22	64	21	130	21
vegetarian dishes	8	3	24	8	32	5
side dish vegetables	9	3	6	2	15	2
**Potatoes, potato dishes**	**3**	**1**	**3**	**1**	**6**	**1**
**Fruit, berries, fruit and berry products**	**7**	**2**	**9**	**3**	**16**	**3**
**Cereals, bakery products**	**13**	**4**	**8**	**2**	**21**	**3**
**Fats, oils, gravy**	**34**	**11**	**128**	**41**	**162**	**26**
margarine and fat spread	18	6	127	41	145	24
blended spread	13	4	0	0	13	2
**Fish, fish dishes**	**3**	**1**	**6**	**2**	**9**	**1**
**Eggs, egg dishes**	**5**	**2**	**5**	**2**	**10**	**2**
**Meat, meat dishes**	**98**	**33**	**27**	**9**	**125**	**20**
red meat dishes	86	29	8	3	94	15
poultry dishes	5	2	8	2	13	2
liver and blood foods	6	2	10	3	16	3
**Milk, dairy products**	**40**	**13**	**22**	**7**	**62**	**10**
milk with 1-1.5% fat content	12	4	4	1	16	3
yoghurt and Finnish cultured milk	8	3	2	1	10	2
cheese	11	4	6	2	17	3
**Sugar, sweets**	**1**	**0**	**0**	**0**	**2**	**0**
**Drinks**	**2**	**1**	**2**	**1**	**4**	**1**
**Miscellaneous**	**12**	**4**	**8**	**3**	**19**	**3**
**Total**	**302**	**100**	**311**	**100**	**613**	**100**

Table S 24: Sources of vitamin A (retinol activity equivalents) (mean and population proportion) in the diet on weekdays among Finnish 5- to 6-year-old preschool children who eat three meals at preschool. Main food groups and selected food groups are presented.

	Outside preschool		Preschool		Total	
	μg/day	%	μg/day	%	μg/day	%
**Vegetables, vegetable dishes**	**85**	**32**	**118**	**30**	**203**	**31**
fresh vegetables and vegetable salads	65	24	77	20	142	22
vegetarian dishes	12	4	31	8	43	6
side dish vegetables	8	3	10	2	18	3
**Potatoes, potato dishes**	**3**	**1**	**4**	**1**	**7**	**1**
**Fruit, berries, fruit and berry products**	**7**	**3**	**5**	**1**	**12**	**2**
**Cereals, bakery products**	**12**	**4**	**11**	**3**	**23**	**3**
**Fats, oils, gravy**	**34**	**12**	**144**	**37**	**178**	**27**
margarine and fat spread	18	7	144	37	162	25
blended spread	12	4	0	0	12	2
**Fish, fish dishes**	**5**	**2**	**6**	**2**	**11**	**2**
**Eggs, egg dishes**	**5**	**2**	**4**	**1**	**9**	**1**
**Meat, meat dishes**	**66**	**24**	**63**	**16**	**129**	**20**
red meat dishes	56	21	11	3	68	10
poultry dishes	5	2	11	3	16	2
liver and blood foods	3	1	40	10	43	7
**Milk, dairy products**	**42**	**16**	**24**	**6**	**66**	**10**
milk with 1-1.5% fat content	9	3	6	2	15	2
yoghurt and Finnish cultured milk	9	3	2	0	11	2
cheese	12	4	6	1	17	3
**Sugar, sweets**	**2**	**1**	**0**	**0**	**2**	**0**
**Drinks**	**0**	**0**	**2**	**1**	**3**	**0**
**Miscellaneous**	**9**	**3**	**7**	**2**	**16**	**2**
**Total**	**270**	**100**	**389**	**100**	**659**	**100**

Table S 25: Sources of vitamin D (mean and population proportion) in the diet on weekdays among Finnish 3- to 4-year-old preschool children who eat three meals at preschool. Main food groups and selected food groups are presented.

	Outside preschool		Preschool		Total	
	**μg/day**	**%**	**μg/day**	**%**	**μg/day**	**%**
**Vegetables, vegetable dishes**	**0.0**	**1.0**	**0.0**	**0.4**	**0.1**	**0.6**
**Potatoes, potato dishes**	**0.0**	**0.8**	**0.0**	**0.1**	**0.0**	**0.4**
**Fruit, berries, fruit and berry products**	**0.0**	**0.1**	**0.0**	**0.5**	**0.0**	**0.4**
**Cereals, bakery products**	**0.2**	**5.2**	**0.2**	**2.4**	**0.4**	**3.3**
**Fats, oils, gravy**	**0.8**	**22.2**	**3.2**	**42.9**	**4.0**	**36.3**
margarine and fat spread	0.4	12.2	3.2	42.9	3.6	33.0
blended spread	0.3	9.6	0.0	0.0	0.3	3.1
**Fish, fish dishes**	**0.3**	**9.5**	**0.5**	**6.6**	**0.8**	**7.5**
**Eggs, egg dishes**	**0.1**	**1.6**	**0.1**	**0.7**	**0.1**	**1.0**
**Meat, meat dishes**	**0.2**	**5.1**	**0.1**	**1.6**	**0.3**	**2.7**
**Milk, dairy products**	**1.8**	**51.7**	**3.2**	**43.6**	**5.1**	**46.2**
skimmed milk	0.8	21.3	2.6	34.8	3.3	30.4
milk with 1-1.5% fat content	0.8	21.8	0.5	6.7	1.3	11.6
yoghurt and Finnish cultured milk	0.1	3.8	0.0	0.5	0.2	1.6
cheese	0.0	0.4	0.0	0.1	0.0	0.2
dairy-based desserts	0.0	0.4	0.0	0.4	0.0	0.4
**Sugar, sweets**	**0.0**	**0.0**	**0.0**	**0.0**	**0.0**	**0.0**
**Drinks**	**0.1**	**2.4**	**0.1**	**1.1**	**0.2**	**1.5**
non-dairy milk substitutes	0.1	1.7	0.1	1.0	0.1	1.3
**Miscellaneous**	**0.0**	**0.3**	**0.0**	**0.0**	**0.0**	**0.1**
**Total**	**3.5**	**100.0**	**7.4**	**100.0**	**10.9**	**100.0**

Table S 26: Sources of vitamin D (mean and population proportion) in the diet on weekdays among Finnish 5- to 6-year-old preschool children who eat three meals at preschool. Main food groups and selected food groups are presented.

	Outside preschool		Preschool		Total	
	**μg/day**	**%**	**μg/day**	**%**	**μg/day**	**%**
**Vegetables, vegetable dishes**	**0.1**	**1.5**	**0.0**	**0.5**	**0.1**	**0.8**
**Potatoes, potato dishes**	**0.0**	**0.8**	**0.0**	**0.2**	**0.0**	**0.4**
**Fruit, berries, fruit and berry products**	**0.0**	**1.2**	**0.0**	**0.5**	**0.1**	**0.7**
**Cereals, bakery products**	**0.2**	**5.0**	**0.2**	**2.6**	**0.4**	**3.4**
**Fats, oils, gravy**	**0.8**	**19.8**	**3.6**	**44.2**	**4.4**	**36.3**
margarine and fat spread	0.4	11.1	3.6	44.2	4.0	33.5
blended spread	0.3	8.0	0.0	0.0	0.3	2.6
**Fish, fish dishes**	**0.4**	**10.9**	**0.5**	**6.4**	**0.9**	**7.8**
**Eggs, egg dishes**	**0.1**	**1.5**	**0.0**	**0.5**	**0.1**	**0.8**
**Meat, meat dishes**	**0.2**	**4.9**	**0.2**	**2.0**	**0.4**	**2.9**
**Milk, dairy products**	**2.0**	**52.1**	**3.5**	**42.7**	**5.5**	**45.7**
skimmed milk	1.0	25.8	2.7	33.3	3.7	30.9
milk with 1-1.5% fat content	0.6	16.2	0.6	6.9	1.2	9.9
yoghurt and Finnish cultured milk	0.1	3.0	0.0	0.5	0.2	1.3
cheese	0.0	0.5	0.0	0.1	0.0	0.2
dairy-based desserts	0.0	0.7	0.0	0.3	0.1	0.5
**Sugar, sweets**	**0.0**	**0.0**	**0.0**	**0.0**	**0.0**	**0.0**
**Drinks**	**0.1**	**2.0**	**0.0**	**0.6**	**0.1**	**1.0**
non-dairy milk substitutes	0.1	2.0	0.0	0.5	0.1	1.0
**Miscellaneous**	**0.0**	**0.2**	**0.0**	**0.0**	**0.0**	**0.0**
**Total**	**3.9**	**100.0**	**8.2**	**100.0**	**12.1**	**100.0**

Table S 27: Sources of vitamin E (mean and population proportion) in the diet on weekdays among Finnish 3- to 4-year-old preschool children who eat three meals at preschool. Main food groups and selected food groups are presented.

	Outside preschool		Preschool		Total	
	mg/day	%	mg/day	%	mg/day	%
**Vegetables, vegetable dishes**	**0.29**	**11.2**	**0.25**	**6.3**	**0.54**	**8.2**
fresh vegetables and vegetable salads	0.17	6.4	0.12	3.0	0.29	4.4
**Potatoes, potato dishes**	**0.06**	**2.2**	**0.03**	**0.7**	**0.09**	**1.3**
**Fruit, berries, fruit and berry products**	**0.27**	**10.4**	**0.19**	**4.8**	**0.46**	**7.0**
**Cereals, bakery products**	**0.52**	**19.7**	**0.60**	**15.3**	**1.11**	**17.0**
**Fats, oils, gravy**	**0.39**	**15.1**	**1.78**	**45.5**	**2.18**	**33.3**
margarine and fat spread	0.24	9.2	1.73	44.2	1.97	30.2
blended spread	0.11	4.4	0.00	0.0	0.11	1.7
**Fish, fish dishes**	**0.17**	**6.4**	**0.36**	**9.2**	**0.53**	**8.1**
**Eggs, egg dishes**	**0.04**	**1.6**	**0.03**	**0.8**	**0.07**	**1.1**
**Meat, meat dishes**	**0.49**	**18.9**	**0.47**	**12.0**	**0.96**	**14.7**
red meat dishes	0.26	9.8	0.21	5.4	0.47	7.2
poultry dishes	0.21	7.9	0.20	5.0	0.40	6.2
**Milk, dairy products**	**0.14**	**5.5**	**0.09**	**2.3**	**0.23**	**3.6**
**Sugar, sweets**	**0.02**	**0.6**	**0.00**	**0.0**	**0.02**	**0.2**
**Drinks**	**0.07**	**2.8**	**0.08**	**1.9**	**0.15**	**2.3**
**Miscellaneous**	**0.15**	**5.6**	**0.05**	**1.3**	**0.20**	**3.0**
**Total**	**2.61**	**100.0**	**3.91**	**100.0**	**6.52**	**100.0**

Table S 28: Sources of vitamin E (mean and population proportion) in the diet on weekdays among Finnish 5- to 6-year-old preschool children who eat three meals at preschool. Main food groups and selected food groups are presented.

	Outside preschool		Preschool		Total	
	mg/day	%	mg/day	%	mg/day	%
**Vegetables, vegetable dishes**	**0.28**	**9.1**	**0.29**	**6.4**	**0.56**	**7.5**
fresh vegetables and vegetable salads	0.16	5.4	0.14	3.2	0.31	4.1
**Potatoes, potato dishes**	**0.13**	**4.4**	**0.04**	**0.9**	**0.17**	**2.3**
**Fruit, berries, fruit and berry products**	**0.38**	**12.7**	**0.18**	**4.1**	**0.57**	**7.6**
**Cereals, bakery products**	**0.57**	**18.8**	**0.70**	**15.5**	**1.26**	**16.8**
**Fats, oils, gravy**	**0.45**	**14.8**	**2.00**	**44.6**	**2.45**	**32.6**
margarine and fat spread	0.25	8.3	1.94	43.2	2.19	29.2
blended spread	0.11	3.6	0.00	0.0	0.11	1.5
**Fish, fish dishes**	**0.20**	**6.6**	**0.43**	**9.6**	**0.63**	**8.4**
**Eggs, egg dishes**	**0.04**	**1.4**	**0.03**	**0.6**	**0.07**	**0.9**
**Meat, meat dishes**	**0.57**	**18.9**	**0.60**	**13.4**	**1.17**	**15.6**
red meat dishes	0.31	10.1	0.27	5.9	0.57	7.6
poultry dishes	0.23	7.7	0.27	6.1	0.51	6.7
**Milk, dairy products**	**0.16**	**5.3**	**0.11**	**2.5**	**0.27**	**3.6**
**Sugar, sweets**	**0.01**	**0.4**	**0.00**	**0.0**	**0.01**	**0.2**
**Drinks**	**0.04**	**1.2**	**0.05**	**1.1**	**0.09**	**1.1**
**Miscellaneous**	**0.19**	**6.3**	**0.06**	**1.2**	**0.25**	**3.3**
**Total**	**3.01**	**100.0**	**4.49**	**100.0**	**7.51**	**100.0**

Table S 29: Sources of thiamine (mean and population proportion) in the diet on weekdays among Finnish 3- to 4-year-old preschool children who eat three meals at preschool. Main food groups and selected food groups are presented.

	**Outside preschool**		**Preschool**		**Total**	
	**mg/day**	**%**	**mg/day**	**%**	**mg/day**	**%**
**Vegetables, vegetable dishes**	**0.03**	**8.2**	**0.03**	**6.3**	**0.06**	**7.1**
**Potatoes, potato dishes**	**0.03**	**7.9**	**0.06**	**12.1**	**0.09**	**10.2**
**Fruit, berries, fruit and berry products**	**0.03**	**7.9**	**0.02**	**4.4**	**0.05**	**5.8**
fresh fruit	0.02	5.4	0.01	1.7	0.03	3.3
**Cereals, bakery products**	**0.07**	**19.6**	**0.12**	**24.9**	**0.19**	**22.5**
rye bread	0.01	2.5	0.02	3.4	0.03	3.0
rye crispbread	0.00	1.1	0.02	5.1	0.03	3.3
multi-grain bread	0.01	3.8	0.04	7.6	0.05	6.0
white bread	0.00	0.5	0.00	0.4	0.00	0.5
porridge	0.01	2.5	0.02	5.1	0.03	4.0
**Fats, oils, gravy**	**0.00**	**0.3**	**0.00**	**0.0**	**0.00**	**0.1**
**Fish, fish dishes**	**0.01**	**2.7**	**0.03**	**5.5**	**0.04**	**4.4**
**Eggs, egg dishes**	**0.00**	**0.8**	**0.00**	**0.6**	**0.01**	**0.7**
**Meat, meat dishes**	**0.09**	**25.1**	**0.10**	**21.1**	**0.19**	**22.9**
cold cuts	0.01	3.5	0.01	2.3	0.02	2.9
red meat dishes	0.06	15.3	0.05	11.0	0.11	12.7
poultry dishes	0.01	3.0	0.02	3.6	0.03	3.5
sausage dishes	0.01	3.0	0.02	4.0	0.03	3.6
**Milk, dairy products**	**0.08**	**21.5**	**0.11**	**22.8**	**0.19**	**22.3**
skimmed milk	0.02	6.3	0.08	16.1	0.10	11.8
milk with 1-1.5% fat content	0.03	9.3	0.02	4.2	0.05	6.4
**Sugar, sweets**	**0.00**	**0.3**	**0.00**	**0.0**	**0.00**	**0.1**
**Drinks**	**0.01**	**2.2**	**0.00**	**0.8**	**0.01**	**1.4**
**Miscellaneous**	**0.02**	**4.1**	**0.01**	**1.1**	**0.02**	**2.4**
**Total**	**0.37**	**100.0**	**0.47**	**100.0**	**0.84**	**100.0**

Table S 30: Sources of thiamine (mean and population proportion) in the diet on weekdays among Finnish 5- to 6-year-old preschool children who eat three meals at preschool. Main food groups and selected food groups are presented.

	Outside preschool		Preschool		Total	
	mg/day	%	mg/day	%	mg/day	%
**Vegetables, vegetable dishes**	**0.03**	**7.7**	**0.04**	**7.4**	**0.07**	**7.4**
**Potatoes, potato dishes**	**0.03**	**8.1**	**0.06**	**12.1**	**0.10**	**10.4**
**Fruit, berries, fruit and berry products**	**0.04**	**8.6**	**0.02**	**4.2**	**0.06**	**6.1**
fresh fruit	0.02	5.3	0.01	1.7	0.03	3.3
**Cereals, bakery products**	**0.08**	**20.1**	**0.13**	**24.1**	**0.21**	**22.3**
rye bread	0.01	2.6	0.01	2.7	0.03	2.6
rye crispbread	0.00	0.7	0.03	5.1	0.03	3.2
multi-grain bread	0.02	4.1	0.04	7.0	0.05	5.7
white bread	0.00	1.0	0.00	0.6	0.01	0.6
porridge	0.01	2.4	0.03	5.1	0.04	3.9
**Fats, oils, gravy**	**0.00**	**0.2**	**0.00**	**0.0**	**0.00**	**0.1**
**Fish, fish dishes**	**0.02**	**3.6**	**0.03**	**5.1**	**0.04**	**4.4**
**Eggs, egg dishes**	**0.00**	**0.7**	**0.00**	**0.4**	**0.01**	**0.5**
**Meat, meat dishes**	**0.11**	**25.1**	**0.12**	**22.2**	**0.22**	**23.5**
cold cuts	0.01	3.3	0.02	3.0	0.03	3.2
red meat dishes	0.06	15.3	0.06	11.9	0.13	13.4
poultry dishes	0.01	2.6	0.02	4.0	0.03	3.5
sausage dishes	0.02	3.6	0.02	2.8	0.03	3.2
**Milk, dairy products**	**0.08**	**20.1**	**0.12**	**22.9**	**0.21**	**21.7**
skimmed milk	0.03	6.9	0.08	15.5	0.11	11.6
milk with 1-1.5% fat content	0.03	6.5	0.03	4.7	0.05	5.5
**Sugar, sweets**	**0.00**	**0.2**	**0.00**	**0.0**	**0.00**	**0.1**
**Drinks**	**0.01**	**1.2**	**0.00**	**0.6**	**0.01**	**0.8**
**Miscellaneous**	**0.02**	**3.8**	**0.01**	**1.1**	**0.02**	**2.3**
**Total**	**0.42**	**100.0**	**0.53**	**100.0**	**0.95**	**100.0**

Table S 31: Sources of riboflavin (mean and population proportion) in the diet on weekdays among Finnish 3- to 4-year-old preschool children who eat three meals at preschool. Main food groups and selected food groups are presented.

	Outside preschool		Preschool		Total	
	mg/day	%	mg/day	%	mg/day	%
**Vegetables, vegetable dishes**	**0.04**	**5.4**	**0.04**	**3.6**	**0.08**	**4.4**
**Potatoes, potato dishes**	**0.01**	**1.2**	**0.01**	**1.3**	**0.02**	**1.3**
**Fruit, berries, fruit and berry products**	**0.03**	**4.3**	**0.03**	**3.1**	**0.06**	**3.6**
**Cereals, bakery products**	**0.08**	**10.1**	**0.11**	**11.1**	**0.19**	**10.7**
**Fats, oils, gravy**	**0.00**	**0.3**	**0.00**	**0.2**	**0.00**	**0.2**
**Fish, fish dishes**	**0.01**	**1.2**	**0.02**	**1.9**	**0.03**	**1.6**
**Eggs, egg dishes**	**0.01**	**1.2**	**0.01**	**0.9**	**0.02**	**1.0**
**Meat, meat dishes**	**0.11**	**13.8**	**0.08**	**8.4**	**0.19**	**10.8**
red meat dishes	0.07	9.6	0.04	4.3	0.12	6.6
poultry dishes	0.02	2.8	0.02	2.3	0.04	2.5
**Milk, dairy products**	**0.45**	**58.5**	**0.66**	**67.9**	**1.11**	**63.8**
skimmed milk	0.15	19.8	0.51	51.8	0.66	37.8
milk with 1-1.5% fat content	0.16	21.0	0.09	9.2	0.25	14.4
**Sugar, sweets**	**0.01**	**0.7**	**0.00**	**0.0**	**0.01**	**0.3**
**Drinks**	**0.02**	**2.2**	**0.01**	**1.1**	**0.03**	**1.6**
**Miscellaneous**	**0.01**	**1.3**	**0.00**	**0.4**	**0.01**	**0.7**
**Total**	**0.76**	**100.0**	**0.98**	**100.0**	**1.74**	**100.0**

Table S 32: Sources of riboflavin (mean and population proportion) in the diet on weekdays among Finnish 5- to 6-year-old preschool children who eat three meals at preschool. Main food groups and selected food groups are presented.

	**Outside preschool**		**Preschool**		**Total**	
	**mg/day**	**%**	**mg/day**	**%**	**mg/day**	**%**
**Vegetables, vegetable dishes**	**0.04**	**5.2**	**0.04**	**4.0**	**0.09**	**4.5**
**Potatoes, potato dishes**	**0.01**	**1.1**	**0.02**	**1.5**	**0.03**	**1.3**
**Fruit, berries, fruit and berry products**	**0.04**	**4.9**	**0.03**	**2.6**	**0.07**	**3.6**
**Cereals, bakery products**	**0.09**	**10.9**	**0.12**	**10.8**	**0.21**	**10.8**
**Fats, oils, gravy**	**0.00**	**0.5**	**0.00**	**0.2**	**0.01**	**0.3**
**Fish, fish dishes**	**0.01**	**1.6**	**0.02**	**1.9**	**0.03**	**1.8**
**Eggs, egg dishes**	**0.01**	**1.2**	**0.01**	**0.6**	**0.02**	**0.8**
**Meat, meat dishes**	**0.11**	**12.7**	**0.11**	**10.0**	**0.22**	**11.2**
red meat dishes	0.07	8.7	0.06	5.2	0.13	6.6
poultry dishes	0.02	2.5	0.03	2.6	0.05	2.5
**Milk, dairy products**	**0.49**	**58.4**	**0.74**	**67.3**	**1.22**	**63.4**
skimmed milk	0.19	23.0	0.54	49.4	0.73	38.0
milk with 1-1.5% fat content	0.13	15.4	0.11	10.4	0.24	12.5
**Sugar, sweets**	**0.01**	**1.0**	**0.00**	**0.1**	**0.01**	**0.5**
**Drinks**	**0.01**	**1.7**	**0.01**	**0.7**	**0.02**	**1.1**
**Miscellaneous**	**0.01**	**1.0**	**0.00**	**0.4**	**0.01**	**0.6**
**Total**	**0.83**	**100.0**	**1.10**	**100.0**	**1.93**	**100.0**

Table S 33: Sources of niacin equivalents (mean and population proportion) in the diet on weekdays among Finnish 3- to 4-year-old preschool children who eat three meals at preschool. Main food groups and selected food groups are presented.

	Outside preschool		Preschool		Total	
	mg/day	%	mg/day	%	mg/day	%
**Vegetables, vegetable dishes**	**0.5**	**5.2**	**0.4**	**4.1**	**0.9**	**4.6**
**Potatoes, potato dishes**	**0.1**	**1.2**	**0.2**	**1.8**	**0.3**	**1.5**
**Fruit, berries, fruit and berry products**	**0.4**	**4.2**	**0.3**	**2.8**	**0.7**	**3.5**
**Cereals, bakery products**	**1.6**	**17.7**	**2.3**	**21.9**	**3.9**	**19.9**
rye bread	0.1	1.3	0.2	2.1	0.3	1.7
rye crispbread	0.0	0.3	0.2	1.9	0.2	1.2
multi-grain bread	0.3	3.3	0.7	6.7	1.0	5.1
white bread	0.1	0.8	0.1	0.6	0.1	0.7
porridge	0.2	2.3	0.7	7.0	0.9	4.8
rice, pasta, etc.	0.2	2.6	0.1	1.0	0.3	1.7
**Fats, oils, gravy**	**0.0**	**0.2**	**0.0**	**0.1**	**0.0**	**0.1**
**Fish, fish dishes**	**0.7**	**7.5**	**1.1**	**10.4**	**1.8**	**9.0**
**Eggs, egg dishes**	**0.1**	**0.9**	**0.1**	**0.7**	**0.2**	**0.8**
**Meat, meat dishes**	**3.2**	**34.9**	**2.6**	**24.5**	**5.7**	**29.4**
cold cuts	0.2	2.4	0.2	2.3	0.5	2.3
red meat dishes	1.8	19.7	1.3	12.0	3.0	15.6
poultry dishes	1.0	10.5	0.7	6.5	1.6	8.4
sausage dishes	0.2	2.1	0.4	3.4	0.6	2.8
**Milk, dairy products**	**2.3**	**25.3**	**3.4**	**32.5**	**5.7**	**29.1**
skimmed milk	0.7	7.8	2.4	22.9	3.1	15.8
milk with 1-1.5% fat content	0.7	7.4	0.4	4.3	1.1	5.7
**Sugar, sweets**	**0.0**	**0.4**	**0.0**	**0.0**	**0.0**	**0.2**
**Drinks**	**0.1**	**1.0**	**0.1**	**0.7**	**0.2**	**0.8**
**Miscellaneous**	**0.1**	**1.6**	**0.0**	**0.3**	**0.2**	**0.9**
**Total**	**9.1**	**100.0**	**10.4**	**100.0**	**19.5**	**100.0**

Table S 34: Sources of niacin equivalents (mean and population proportion) in the diet on weekdays among Finnish 5- to 6-year-old preschool children who eat three meals at preschool. Main food groups and selected food groups are presented.

	**Outside preschool**		**Preschool**		**Total**	
	**mg/day**	**%**	**mg/day**	**%**	**mg/day**	**%**
**Vegetables, vegetable dishes**	**0.5**	**4.7**	**0.5**	**4.5**	**1.0**	**4.6**
**Potatoes, potato dishes**	**0.1**	**1.4**	**0.2**	**1.9**	**0.4**	**1.7**
**Fruit, berries, fruit and berry products**	**0.5**	**4.4**	**0.3**	**2.5**	**0.8**	**3.4**
**Cereals, bakery products**	**1.9**	**18.4**	**2.5**	**21.8**	**4.5**	**20.2**
rye bread	0.2	1.5	0.2	1.6	0.3	1.6
rye crispbread	0.0	0.2	0.2	1.9	0.2	1.1
multi-grain bread	0.4	3.5	0.7	6.0	1.1	4.8
white bread	0.1	1.0	0.1	1.0	0.2	1.0
porridge	0.2	2.0	0.8	7.2	1.0	4.7
rice, pasta, etc.	0.3	2.5	0.1	1.1	0.4	1.7
**Fats, oils, gravy**	**0.0**	**0.3**	**0.0**	**0.1**	**0.0**	**0.2**
**Fish, fish dishes**	**0.7**	**7.1**	**1.1**	**9.8**	**1.9**	**8.5**
**Eggs, egg dishes**	**0.1**	**0.8**	**0.1**	**0.5**	**0.1**	**0.7**
**Meat, meat dishes**	**3.7**	**35.4**	**3.0**	**25.5**	**6.6**	**30.2**
cold cuts	0.3	2.6	0.3	2.5	0.6	2.5
red meat dishes	2.1	20.7	1.4	12.0	3.5	16.1
poultry dishes	1.0	9.4	0.9	7.7	1.9	8.5
sausage dishes	0.3	2.6	0.3	2.4	0.5	2.5
**Milk, dairy products**	**2.5**	**24.5**	**3.8**	**32.4**	**6.3**	**28.7**
skimmed milk	0.9	8.7	2.6	21.9	3.5	15.7
milk with 1-1.5% fat content	0.5	5.2	0.6	4.7	1.1	5.0
**Sugar, sweets**	**0.0**	**0.5**	**0.0**	**0.1**	**0.1**	**0.2**
**Drinks**	**0.1**	**0.6**	**0.0**	**0.4**	**0.1**	**0.5**
**Miscellaneous**	**0.2**	**1.8**	**0.0**	**0.4**	**0.2**	**1.1**
**Total**	**10.3**	**100.0**	**11.7**	**100.0**	**22.0**	**100.0**

Table S 35: Sources of vitamin B6 (mean and population proportion) in the diet on weekdays among Finnish 3- to 4-year-old preschool children who eat three meals at preschool. Main food groups and selected food groups are presented.

	Outside preschool		Preschool		Total	
	mg/day	%	mg/day	%	mg/day	%
**Vegetables, vegetable dishes**	**0.04**	**7.8**	**0.04**	**5.6**	**0.08**	**6.7**
**Potatoes, potato dishes**	**0.02**	**3.4**	**0.04**	**6.3**	**0.06**	**4.9**
**Fruit, berries, fruit and berry products**	**0.12**	**21.2**	**0.07**	**10.8**	**0.19**	**15.8**
**Cereals, bakery products**	**0.07**	**11.5**	**0.11**	**17.0**	**0.17**	**14.4**
**Fats, oils, gravy**	**0.00**	**0.2**	**0.00**	**0.0**	**0.00**	**0.1**
**Fish, fish dishes**	**0.03**	**5.8**	**0.06**	**9.2**	**0.09**	**7.6**
**Eggs, egg dishes**	**0.00**	**0.7**	**0.00**	**0.5**	**0.01**	**0.6**
**Meat, meat dishes**	**0.12**	**21.8**	**0.12**	**19.3**	**0.24**	**20.5**
**Milk, dairy products**	**0.12**	**21.1**	**0.18**	**28.8**	**0.30**	**25.2**
skimmed milk	0.04	7.1	0.14	21.9	0.18	14.8
milk with 1-1.5% fat content	0.04	7.6	0.03	4.2	0.07	5.8
**Sugar, sweets**	**0.00**	**0.2**	**0.00**	**0.0**	**0.00**	**0.1**
**Drinks**	**0.02**	**3.7**	**0.01**	**1.6**	**0.03**	**2.6**
**Miscellaneous**	**0.01**	**2.5**	**0.00**	**0.6**	**0.02**	**1.6**
**Total**	**0.57**	**100.0**	**0.62**	**100.0**	**1.19**	**100.0**

Table S 36: Sources of vitamin B6 (mean and population proportion) in the diet on weekdays among Finnish 5- to 6-year-old preschool children who eat three meals at preschool. Main food groups and selected food groups are presented.

	Outside preschool		Preschool		Total	
	mg/day	%	mg/day	%	mg/day	%
**Vegetables, vegetable dishes**	**0.04**	**6.9**	**0.04**	**6.1**	**0.09**	**6.5**
**Potatoes, potato dishes**	**0.02**	**3.4**	**0.05**	**6.6**	**0.07**	**5.1**
**Fruit, berries, fruit and berry products**	**0.13**	**20.5**	**0.07**	**10.5**	**0.20**	**15.2**
**Cereals, bakery products**	**0.08**	**12.0**	**0.12**	**16.5**	**0.19**	**14.3**
**Fats, oils, gravy**	**0.00**	**0.2**	**0.00**	**0.1**	**0.00**	**0.2**
**Fish, fish dishes**	**0.04**	**5.6**	**0.06**	**8.8**	**0.10**	**7.3**
**Eggs, egg dishes**	**0.00**	**0.6**	**0.00**	**0.4**	**0.01**	**0.5**
**Meat, meat dishes**	**0.14**	**22.8**	**0.15**	**20.8**	**0.29**	**21.7**
**Milk, dairy products**	**0.13**	**20.8**	**0.20**	**28.1**	**0.33**	**24.7**
skimmed milk	0.05	8.2	0.15	20.6	0.20	14.8
milk with 1-1.5% fat content	0.04	5.6	0.03	4.5	0.07	5.0
**Sugar, sweets**	**0.00**	**0.3**	**0.00**	**0.0**	**0.00**	**0.2**
**Drinks**	**0.03**	**4.0**	**0.01**	**1.3**	**0.03**	**2.6**
**Miscellaneous**	**0.02**	**2.9**	**0.01**	**0.8**	**0.02**	**1.8**
**Total**	**0.62**	**100.0**	**0.71**	**100.0**	**1.33**	**100.0**

Table S 37: Sources of total folate including folic acid (mean and population proportion) in the diet on weekdays among Finnish 3- to 4-year-old preschool children who eat three meals at preschool. Main food groups and selected food groups are presented.

	Outside preschool		Preschool		Total	
	μg/day	%	μg/day	%	μg/day	%
**Vegetables, vegetable dishes**	**10**	**14.3**	**11**	**12.8**	**21**	**13.5**
fresh vegetables and vegetable salads	6	8.8	7	8.6	13	8.7
vegetarian dishes	2	3.1	3	3.4	5	3.3
warm side dish vegetables	1	2.1	1	0.9	2	1.4
**Potatoes, potato dishes**	**3**	**3.9**	**5**	**5.7**	**8**	**4.9**
**Fruit, berries, fruit and berry products**	**8**	**11.9**	**7**	**7.8**	**15**	**9.6**
fresh fruit	5	7.5	2	2.4	7	4.6
100% juice	1	1.6	1	1.0	2	1.2
**Cereals, bakery products**	**17**	**24.9**	**30**	**34.6**	**47**	**30.3**
rye bread	4	5.4	7	7.5	10	6.6
rye crispbread	1	1.4	7	7.6	8	4.9
multi-grain bread	4	5.2	9	10.0	12	7.9
white bread	1	1.1	1	0.8	1	0.9
porridge	2	2.5	5	5.9	7	4.4
rice, pasta, etc.	1	2.1	1	0.7	2	1.3
**Fats, oils, gravy**	**0**	**0.3**	**0**	**0.1**	**0**	**0.2**
**Fish, fish dishes**	**1**	**1.8**	**4**	**4.7**	**5**	**3.4**
**Eggs, egg dishes**	**1**	**2.2**	**1**	**1.6**	**3**	**1.8**
**Meat, meat dishes**	**12**	**17.4**	**10**	**11.4**	**22**	**14.0**
red meat dishes	9	13.1	6	6.7	15	9.5
poultry dishes	2	2.9	2	2.7	4	2.8
**Milk, dairy products**	**12**	**18.0**	**17**	**19.4**	**29**	**18.8**
skimmed milk	3	4.9	11	12.8	14	9.4
milk with 1-1.5% fat content	4	5.2	2	2.4	6	3.6
yoghurt and Finnish cultured milk	2	3.0	1	0.6	3	1.7
cheese	1	2.1	1	0.7	2	1.3
**Sugar, sweets**	**0**	**0.2**	**0**	**0.0**	**0**	**0.1**
**Drinks**	**2**	**2.8**	**1**	**1.6**	**3**	**2.1**
non-dairy milk substitutes	1	1.9	1	1.5	3	1.7
**Miscellaneous**	**2**	**2.3**	**0**	**0.3**	**2**	**1.2**
**Total**	**68**	**100.0**	**87**	**100.0**	**154**	**100.0**

Table S 38: Sources of total folate including folic acid (mean and population proportion) in the diet on weekdays among Finnish 5- to 6-year-old preschool children who eat three meals at preschool. Main food groups and selected food groups are presented.

	Outside preschool		Preschool		Total	
	μg/day	%	μg/day	%	μg/day	%
**Vegetables, vegetable dishes**	**10**	**13.5**	**14**	**14.2**	**24**	**13.9**
fresh vegetables and vegetable salads	7	8.9	10	9.8	16	9.4
vegetarian dishes	2	2.4	3	3.4	5	3.0
warm side dish vegetables	1	2.0	1	1.0	2	1.4
**Potatoes, potato dishes**	**3**	**4.1**	**5**	**5.4**	**8**	**4.9**
**Fruit, berries, fruit and berry products**	**11**	**14.7**	**7**	**6.7**	**18**	**10.1**
fresh fruit	6	7.6	2	1.9	8	4.3
100% juice	2	2.4	1	1.3	3	1.8
**Cereals, bakery products**	**19**	**26.2**	**33**	**32.6**	**52**	**29.9**
rye bread	4	5.9	6	5.8	10	5.9
rye crispbread	1	0.9	7	7.3	8	4.6
multi-grain bread	4	5.7	9	9.2	14	7.8
white bread	1	1.3	1	1.2	2	1.2
porridge	2	2.4	6	6.1	8	4.5
rice, pasta, etc.	1	2.0	1	0.7	2	1.2
**Fats, oils, gravy**	**0**	**0.5**	**0**	**0.1**	**1**	**0.3**
**Fish, fish dishes**	**2**	**2.4**	**5**	**4.8**	**7**	**3.8**
**Eggs, egg dishes**	**1**	**2.0**	**1**	**1.1**	**3**	**1.5**
**Meat, meat dishes**	**10**	**13.3**	**15**	**15.1**	**25**	**14.3**
red meat dishes	7	10.1	7	6.5	14	8.0
poultry dishes	2	2.4	3	3.1	5	2.8
**Milk, dairy products**	**14**	**19.5**	**19**	**18.4**	**33**	**18.9**
skimmed milk	5	6.5	12	11.9	17	9.6
milk with 1-1.5% fat content	3	4.0	3	2.6	6	3.2
yoghurt and Finnish cultured milk	2	3.2	0	0.5	3	1.6
cheese	1	1.9	1	0.7	2	1.2
**Sugar, sweets**	**0**	**0.2**	**0**	**0.0**	**0**	**0.1**
**Drinks**	**1**	**1.6**	**1**	**1.2**	**2**	**1.4**
non-dairy milk substitutes	1	0.9	1	1.1	2	1.0
**Miscellaneous**	**2**	**2.0**	**0**	**0.3**	**2**	**1.1**
**Total**	**74**	**100.0**	**100**	**100.0**	**174**	**100.0**

Table S 39: Sources of vitamin B12 (mean and population proportion) in the diet on weekdays among Finnish 3- to 4-year-old preschool children who eat three meals at preschool. Main food groups and selected food groups are presented.

	Outside preschool		Preschool		Total	
	μg/day	%	μg/day	%	μg/day	%
**Vegetables, vegetable dishes**	**0.02**	**0.9**	**0.02**	**1.0**	**0.04**	**1.0**
**Potatoes, potato dishes**	**0.01**	**0.4**	**0.00**	**0.1**	**0.01**	**0.2**
**Fruit, berries, fruit and berry products**	**0.01**	**0.3**	**0.02**	**0.9**	**0.03**	**0.6**
**Cereals, bakery products**	**0.10**	**4.5**	**0.09**	**3.8**	**0.19**	**4.1**
**Fats, oils, gravy**	**0.01**	**0.3**	**0.00**	**0.1**	**0.01**	**0.2**
**Fish, fish dishes**	**0.28**	**12.5**	**0.31**	**13.9**	**0.59**	**13.2**
**Eggs, egg dishes**	**0.06**	**2.7**	**0.06**	**2.5**	**0.12**	**2.6**
**Meat, meat dishes**	**0.77**	**34.7**	**0.29**	**12.8**	**1.06**	**23.7**
cold cuts	0.02	0.7	0.02	0.7	0.03	0.7
red meat dishes	0.63	28.3	0.15	6.7	0.78	17.4
poultry dishes	0.09	4.2	0.07	3.0	0.16	3.6
sausage dishes	0.02	0.8	0.03	1.5	0.05	1.2
**Milk, dairy products**	**0.93**	**41.9**	**1.42**	**63.1**	**2.35**	**52.6**
skimmed milk	0.32	14.2	1.06	47.0	1.38	30.7
milk with 1-1.5% fat content	0.34	15.2	0.20	8.9	0.54	12.0
yoghurt and Finnish cultured milk	0.10	4.5	0.03	1.1	0.12	2.8
cheese	0.07	3.3	0.06	2.6	0.13	3.0
dairy-based desserts	0.02	1.1	0.04	1.9	0.07	1.5
ice-cream	0.01	0.5	0.00	0.1	0.02	0.3
**Sugar, sweets**	**0.00**	**0.0**	**0.00**	**0.0**	**0.00**	**0.0**
**Drinks**	**0.04**	**1.6**	**0.04**	**1.8**	**0.08**	**1.7**
**Miscellaneous**	**0.00**	**0.1**	**0.00**	**0.0**	**0.00**	**0.1**
**Total**	**2.22**	**100.0**	**2.25**	**100.0**	**4.48**	**100.0**

Table S 40: Sources of vitamin B12 (mean and population proportion) in the diet on weekdays among Finnish 5- to 6-year-old preschool children who eat three meals at preschool. Main food groups and selected food groups are presented.

	Outside preschool		Preschool		Total	
	μg/day	%	μg/day	%	μg/day	%
**Vegetables, vegetable dishes**	**0.03**	**1.4**	**0.03**	**1.1**	**0.06**	**1.3**
**Potatoes, potato dishes**	**0.01**	**0.4**	**0.00**	**0.1**	**0.01**	**0.2**
**Fruit, berries, fruit and berry products**	**0.01**	**0.5**	**0.02**	**0.7**	**0.03**	**0.6**
**Cereals, bakery products**	**0.12**	**5.2**	**0.10**	**3.8**	**0.21**	**4.5**
**Fats, oils, gravy**	**0.02**	**0.7**	**0.00**	**0.1**	**0.02**	**0.4**
**Fish, fish dishes**	**0.29**	**13.0**	**0.32**	**12.5**	**0.61**	**12.7**
**Eggs, egg dishes**	**0.06**	**2.6**	**0.05**	**1.8**	**0.10**	**2.2**
**Meat, meat dishes**	**0.70**	**30.8**	**0.40**	**15.7**	**1.10**	**22.8**
cold cuts	0.02	0.8	0.02	0.7	0.04	0.8
red meat dishes	0.56	24.5	0.18	7.1	0.73	15.3
poultry dishes	0.09	3.9	0.09	3.4	0.18	3.7
sausage dishes	0.03	1.1	0.03	1.0	0.05	1.1
**Milk, dairy products**	**1.00**	**44.2**	**1.58**	**62.5**	**2.59**	**53.9**
skimmed milk	0.41	17.9	1.14	44.8	1.54	32.1
milk with 1-1.5% fat content	0.27	11.9	0.25	9.9	0.52	10.8
yoghurt and Finnish cultured milk	0.11	4.9	0.02	0.8	0.13	2.7
cheese	0.07	2.9	0.07	2.6	0.13	2.7
dairy-based desserts	0.04	1.7	0.05	2.1	0.09	1.9
ice-cream	0.02	0.7	0.01	0.2	0.02	0.5
**Sugar, sweets**	**0.00**	**0.0**	**0.00**	**0.0**	**0.00**	**0.0**
**Drinks**	**0.03**	**1.4**	**0.04**	**1.5**	**0.07**	**1.5**
**Miscellaneous**	**0.00**	**0.0**	**0.00**	**0.0**	**0.00**	**0.0**
**Total**	**2.27**	**100.0**	**2.53**	**100.0**	**4.80**	**100.0**

Table S 41: Sources of vitamin C (mean and population proportion) in the diet on weekdays among Finnish 3- to 4-year-old preschool children who eat three meals at preschool. Main food groups and selected food groups are presented.

	Outside preschool		Preschool		Total	
	mg/day	%	mg/day	%	mg/day	%
**Vegetables, vegetable dishes**	**9.0**	**27.7**	**7.1**	**21.6**	**16.1**	**24.6**
fresh vegetables and vegetable salads	6.9	21.3	5.8	17.6	12.7	19.5
vegetarian dishes	0.9	2.7	1.0	2.9	1.8	2.8
warm side dish vegetables	1.2	3.5	0.3	1.0	1.5	2.3
**Potatoes, potato dishes**	**1.3**	**3.9**	**2.6**	**7.8**	**3.8**	**5.8**
**Fruit, berries, fruit and berry products**	**14.3**	**44.1**	**13.4**	**40.6**	**27.7**	**42.3**
fresh fruit	8.4	25.7	3.5	10.6	11.8	18.1
berries	1.8	5.6	0.1	0.4	2.0	3.0
fruit and berry soups	0.4	1.1	3.9	11.9	4.3	6.6
100% juice	2.6	7.9	2.3	6.8	4.8	7.4
smoothies	0.3	0.9	1.1	3.3	1.4	2.1
**Cereals, bakery products**	**0.4**	**1.4**	**0.6**	**1.9**	**1.1**	**1.6**
**Fats, oils, gravy**	**0.0**	**0.0**	**0.0**	**0.1**	**0.0**	**0.1**
**Fish, fish dishes**	**0.2**	**0.5**	**0.9**	**2.7**	**1.0**	**1.6**
**Eggs, egg dishes**	**0.0**	**0.0**	**0.0**	**0.0**	**0.0**	**0.0**
**Meat, meat dishes**	**2.6**	**7.9**	**2.9**	**8.8**	**5.5**	**8.4**
**Milk, dairy products**	**3.3**	**10.0**	**5.0**	**15.3**	**8.3**	**12.7**
**Sugar, sweets**	**0.0**	**0.1**	**0.0**	**0.0**	**0.0**	**0.0**
**Drinks**	**0.9**	**2.7**	**0.2**	**0.7**	**1.1**	**1.7**
**Miscellaneous**	**0.5**	**1.7**	**0.2**	**0.5**	**0.7**	**1.1**
**Total**	**32.5**	**100.0**	**32.9**	**100.0**	**65.4**	**100.0**

Table S 42: Sources of vitamin C (mean and population proportion) in the diet on weekdays among Finnish 5- to 6-year-old preschool children who eat three meals at preschool. Main food groups and selected food groups are presented.

	Outside preschool		Preschool		Total	
	mg/day	%	mg/day	%	mg/day	%
**Vegetables, vegetable dishes**	**8.8**	**22.7**	**8.4**	**23.9**	**17.2**	**23.3**
fresh vegetables and vegetable salads	6.8	17.6	6.7	19.1	13.6	18.3
vegetarian dishes	1.1	2.7	1.1	3.2	2.2	2.9
warm side dish vegetables	0.9	2.3	0.6	1.6	1.5	2.0
**Potatoes, potato dishes**	**1.4**	**3.6**	**2.9**	**8.1**	**4.3**	**5.7**
**Fruit, berries, fruit and berry products**	**20.1**	**51.8**	**12.4**	**35.1**	**32.5**	**43.9**
fresh fruit	9.5	24.4	3.5	10.0	13.0	17.5
berries	3.3	8.6	0.1	0.3	3.5	4.7
fruit and berry soups	1.4	3.6	2.0	5.8	3.4	4.6
100% juice	4.2	10.8	2.9	8.1	7.0	9.5
smoothies	0.5	1.4	1.0	2.8	1.5	2.1
**Cereals, bakery products**	**0.7**	**1.7**	**1.1**	**3.0**	**1.7**	**2.3**
**Fats, oils, gravy**	**0.0**	**0.1**	**0.1**	**0.1**	**0.1**	**0.1**
**Fish, fish dishes**	**0.4**	**1.0**	**0.9**	**2.4**	**1.2**	**1.7**
**Eggs, egg dishes**	**0.0**	**0.0**	**0.0**	**0.0**	**0.0**	**0.0**
**Meat, meat dishes**	**2.1**	**5.5**	**3.6**	**10.2**	**5.7**	**7.7**
**Milk, dairy products**	**3.4**	**8.9**	**5.6**	**15.7**	**9.0**	**12.2**
**Sugar, sweets**	**0.3**	**0.9**	**0.0**	**0.0**	**0.3**	**0.5**
**Drinks**	**1.2**	**3.0**	**0.4**	**1.0**	**1.5**	**2.0**
**Miscellaneous**	**0.3**	**0.8**	**0.1**	**0.4**	**0.5**	**0.6**
**Total**	**38.7**	**100.0**	**35.3**	**100.0**	**74.0**	**100.0**

Table S 43: Sources of salt (mean and population proportion) in the diet on weekdays among Finnish 3- to 4-year-old preschool children who eat three meals at preschool. Main food groups and selected food groups are presented.

	**Outside preschool**		**Preschool**		**Total**	
	**g/day**	**%**	**g/day**	**%**	**g/day**	**%**
**Vegetables, vegetable dishes**	**0.1**	**4.6**	**0.1**	**3.7**	**0.2**	**4.1**
**Potatoes, potato dishes**	**0.1**	**3.6**	**0.0**	**1.1**	**0.1**	**2.1**
**Fruit, berries, fruit and berry products**	**0.0**	**0.3**	**0.0**	**0.5**	**0.0**	**0.4**
**Cereals, bakery products**	**0.6**	**26.5**	**1.3**	**41.0**	**1.9**	**35.2**
rye bread	0.1	3.6	0.1	4.0	0.2	3.8
rye crispbread	0.0	1.0	0.2	5.0	0.2	3.5
multi-grain bread	0.1	4.7	0.2	6.8	0.3	6.0
white bread	0.0	1.1	0.0	0.9	0.1	1.0
porridge	0.1	5.2	0.7	21.1	0.8	14.8
rice, pasta, etc.	0.1	3.0	0.0	0.3	0.1	1.4
savoury bakery products, hamburgers, pizza	0.1	3.0	0.0	0.8	0.1	1.7
pancakes, crpes	0.0	0.7	0.0	1.0	0.0	0.9
**Fats, oils, gravy**	**0.1**	**3.3**	**0.2**	**6.0**	**0.3**	**4.9**
**Fish, fish dishes**	**0.1**	**5.6**	**0.2**	**7.6**	**0.4**	**6.8**
**Eggs, egg dishes**	**0.0**	**0.6**	**0.0**	**0.2**	**0.0**	**0.3**
**Meat, meat dishes**	**0.7**	**32.2**	**0.8**	**23.7**	**1.4**	**27.1**
cold cuts	0.1	2.6	0.0	1.4	0.1	1.9
red meat dishes	0.4	19.1	0.4	11.1	0.8	14.3
poultry dishes	0.1	6.7	0.2	6.2	0.3	6.4
sausage dishes	0.1	3.6	0.2	4.8	0.2	4.3
**Milk, dairy products**	**0.4**	**17.2**	**0.4**	**14.0**	**0.8**	**15.3**
skimmed milk	0.1	4.2	0.3	9.3	0.4	7.3
milk with 1-1.5% fat content	0.1	4.2	0.1	1.6	0.1	2.6
yoghurt and Finnish cultured milk	0.0	2.0	0.0	0.4	0.1	1.0
cheese	0.1	4.4	0.1	1.7	0.1	2.8
**Sugar, sweets**	**0.0**	**0.4**	**0.0**	**0.0**	**0.0**	**0.2**
**Drinks**	**0.0**	**0.9**	**0.0**	**0.4**	**0.0**	**0.6**
**Miscellaneous**	**0.1**	**4.9**	**0.1**	**1.7**	**0.2**	**3.0**
**Total**	**2.1**	**100.0**	**3.2**	**100.0**	**5.3**	**100.0**

Table S 44: Sources of salt (mean and population proportion) in the diet on weekdays among Finnish 5- to 6-year-old preschool children who eat three meals at preschool. Main food groups and selected food groups are presented.

	Outside preschool		Preschool		Total	
	g/day	%	g/day	%	g/day	%
**Vegetables, vegetable dishes**	**0.1**	**4.4**	**0.2**	**4.2**	**0.3**	**4.3**
**Potatoes, potato dishes**	**0.1**	**3.5**	**0.1**	**1.5**	**0.1**	**2.3**
**Fruit, berries, fruit and berry products**	**0.0**	**0.5**	**0.0**	**0.4**	**0.0**	**0.5**
**Cereals, bakery products**	**0.6**	**26.7**	**1.5**	**40.6**	**2.1**	**35.1**
rye bread	0.1	3.7	0.1	3.1	0.2	3.3
rye crispbread	0.0	0.7	0.2	4.9	0.2	3.2
multi-grain bread	0.1	5.3	0.2	6.2	0.4	5.9
white bread	0.0	1.3	0.0	1.4	0.1	1.3
porridge	0.1	5.0	0.8	21.5	0.9	14.9
rice, pasta, etc.	0.1	2.7	0.0	0.3	0.1	1.3
savoury bakery products, hamburgers, pizza	0.1	2.7	0.0	1.2	0.1	1.8
pancakes, crpes	0.0	0.5	0.0	1.0	0.0	0.8
**Fats, oils, gravy**	**0.1**	**3.0**	**0.2**	**6.2**	**0.3**	**4.9**
**Fish, fish dishes**	**0.2**	**7.3**	**0.2**	**6.7**	**0.4**	**6.9**
**Eggs, egg dishes**	**0.0**	**0.6**	**0.0**	**0.1**	**0.0**	**0.3**
**Meat, meat dishes**	**0.7**	**31.4**	**0.9**	**24.8**	**1.6**	**27.4**
cold cuts	0.1	2.6	0.1	1.6	0.1	2.0
red meat dishes	0.5	19.0	0.4	11.9	0.9	14.8
poultry dishes	0.1	5.5	0.3	7.4	0.4	6.6
sausage dishes	0.1	4.0	0.1	3.5	0.2	3.7
**Milk, dairy products**	**0.4**	**16.7**	**0.5**	**13.8**	**0.9**	**14.9**
skimmed milk	0.1	4.7	0.3	8.8	0.4	7.1
milk with 1-1.5% fat content	0.1	2.9	0.1	1.8	0.1	2.3
yoghurt and Finnish cultured milk	0.0	2.1	0.0	0.3	0.1	1.0
cheese	0.1	4.4	0.1	1.4	0.2	2.6
**Sugar, sweets**	**0.0**	**1.0**	**0.0**	**0.1**	**0.0**	**0.4**
**Drinks**	**0.0**	**0.5**	**0.0**	**0.2**	**0.0**	**0.3**
**Miscellaneous**	**0.1**	**4.5**	**0.1**	**1.5**	**0.2**	**2.7**
**Total**	**2.4**	**100.0**	**3.6**	**100.0**	**6.0**	**100.0**

Table S 45: Sources of potassium (mean and population proportion) in the diet on weekdays among Finnish 3- to 4-year-old preschool children who eat three meals at preschool. Main food groups and selected food groups are presented.

	Outside preschool		Preschool		Total	
	mg/day	%	mg/day	%	mg/day	%
**Vegetables, vegetable dishes**	**139**	**12**	**136**	**9**	**275**	**10**
**Potatoes, potato dishes**	**77**	**7**	**154**	**10**	**231**	**9**
**Fruit, berries, fruit and berry products**	**159**	**14**	**108**	**7**	**267**	**10**
**Cereals, bakery products**	**127**	**11**	**240**	**16**	**367**	**14**
**Fats, oils, gravy**	**2**	**0**	**2**	**0**	**4**	**0**
**Fish, fish dishes**	**34**	**3**	**91**	**6**	**125**	**5**
**Eggs, egg dishes**	**3**	**0**	**3**	**0**	**6**	**0**
**Meat, meat dishes**	**171**	**15**	**209**	**14**	**380**	**14**
**Milk, dairy products**	**371**	**33**	**558**	**37**	**929**	**35**
skimmed milk	127	11	424	28	550	21
milk with 1-1.5% fat content	126	11	75	5	201	8
**Sugar, sweets**	**9**	**1**	**2**	**0**	**11**	**0**
**Drinks**	**15**	**1**	**8**	**1**	**23**	**1**
**Miscellaneous**	**27**	**2**	**10**	**1**	**37**	**1**
**Total**	**1135**	**100**	**1521**	**100**	**2656**	**100**

Table S 46: Sources of potassium (mean and population proportion) in the diet on weekdays among Finnish 5- to 6-year-old preschool children who eat three meals at preschool. Main food groups and selected food groups are presented.

	mg/day	%	mg/day	%	mg/day	%
**Vegetables, vegetable dishes**	**146**	**11**	**168**	**10**	**315**	**10**
**Potatoes, potato dishes**	**89**	**7**	**179**	**10**	**268**	**9**
**Fruit, berries, fruit and berry products**	**181**	**14**	**110**	**6**	**291**	**10**
**Cereals, bakery products**	**141**	**11**	**266**	**15**	**407**	**14**
**Fats, oils, gravy**	**3**	**0**	**2**	**0**	**5**	**0**
**Fish, fish dishes**	**48**	**4**	**98**	**6**	**146**	**5**
**Eggs, egg dishes**	**4**	**0**	**2**	**0**	**6**	**0**
**Meat, meat dishes**	**190**	**15**	**259**	**15**	**449**	**15**
**Milk, dairy products**	**411**	**32**	**622**	**36**	**1034**	**34**
skimmed milk	159	13	454	26	614	20
milk with 1-1.5% fat content	101	8	94	5	195	6
**Sugar, sweets**	**15**	**1**	**3**	**0**	**18**	**1**
**Drinks**	**11**	**1**	**5**	**0**	**16**	**1**
**Miscellaneous**	**34**	**3**	**11**	**1**	**44**	**1**
**Total**	**1274**	**100**	**1726**	**100**	**2999**	**100**

Table S 47: Sources of phosphorous (mean and population proportion) in the diet on weekdays among Finnish 3- to 4-year-old preschool children who eat three meals at preschool. Main food groups and selected food groups are presented.

	Outside preschool		Preschool		Total	
	mg/day	%	mg/day	%	mg/day	%
**Vegetables, vegetable dishes**	**23**	**5**	**23**	**3**	**46**	**4**
**Potatoes, potato dishes**	**9**	**2**	**17**	**3**	**27**	**2**
**Fruit, berries, fruit and berry products**	**16**	**3**	**16**	**2**	**32**	**3**
**Cereals, bakery products**	**97**	**19**	**176**	**26**	**273**	**23**
**Fats, oils, gravy**	**2**	**0**	**1**	**0**	**3**	**0**
**Fish, fish dishes**	**17**	**3**	**32**	**5**	**49**	**4**
**Eggs, egg dishes**	**5**	**1**	**5**	**1**	**10**	**1**
**Meat, meat dishes**	**81**	**16**	**71**	**10**	**152**	**13**
**Milk, dairy products**	**235**	**47**	**335**	**49**	**570**	**48**
skimmed milk	71	14	238	35	309	26
milk with 1-1.5% fat content	76	15	43	6	119	10
**Sugar, sweets**	**4**	**1**	**0**	**0**	**4**	**0**
**Drinks**	**6**	**1**	**5**	**1**	**11**	**1**
**Miscellaneous**	**8**	**2**	**2**	**0**	**10**	**1**
**Total**	**503**	**100**	**684**	**100**	**1187**	**100**

Table S 48: Sources of phosphorous (mean and population proportion) in the diet on weekdays among Finnish 5- to 6-year-old preschool children who eat three meals at preschool. Main food groups and selected food groups are presented.

	Outside preschool		Preschool		Total	
	mg/day	%	mg/day	%	mg/day	%
**Vegetables, vegetable dishes**	**24**	**4**	**29**	**4**	**53**	**4**
**Potatoes, potato dishes**	**10**	**2**	**20**	**3**	**30**	**2**
**Fruit, berries, fruit and berry products**	**20**	**4**	**16**	**2**	**35**	**3**
**Cereals, bakery products**	**106**	**19**	**194**	**25**	**299**	**23**
**Fats, oils, gravy**	**3**	**0**	**1**	**0**	**4**	**0**
**Fish, fish dishes**	**21**	**4**	**35**	**5**	**56**	**4**
**Eggs, egg dishes**	**5**	**1**	**4**	**1**	**9**	**1**
**Meat, meat dishes**	**90**	**16**	**89**	**12**	**179**	**14**
**Milk, dairy products**	**261**	**47**	**374**	**49**	**635**	**48**
skimmed milk	90	16	256	33	345	26
milk with 1-1.5% fat content	60	11	54	7	115	9
**Sugar, sweets**	**6**	**1**	**1**	**0**	**7**	**1**
**Drinks**	**4**	**1**	**2**	**0**	**6**	**0**
**Miscellaneous**	**9**	**2**	**2**	**0**	**12**	**1**
**Total**	**559**	**100**	**767**	**100**	**1326**	**100**

Table S 49: Sources of calcium (mean and population proportion) in the diet on weekdays among Finnish 3- to 4-year-old preschool children who eat three meals at preschool. Main food groups and selected food groups are presented.

	**Outside preschool**		**Preschool**		**Total**	
	**mg/day**	**%**	**mg/day**	**%**	**mg/day**	**%**
**Vegetables, vegetable dishes**	**15**	**3.4**	**15**	**2.6**	**30**	**3.0**
**Potatoes, potato dishes**	**3**	**0.8**	**4**	**0.7**	**8**	**0.8**
**Fruit, berries, fruit and berry products**	**12**	**2.8**	**18**	**3.1**	**30**	**3.0**
**Cereals, bakery products**	**31**	**7.1**	**39**	**6.9**	**70**	**7.0**
**Fats, oils, gravy**	**2**	**0.3**	**2**	**0.3**	**3**	**0.3**
**Fish, fish dishes**	**4**	**1.0**	**9**	**1.5**	**13**	**1.3**
**Eggs, egg dishes**	**2**	**0.4**	**1**	**0.2**	**3**	**0.3**
**Meat, meat dishes**	**21**	**4.8**	**20**	**3.5**	**41**	**4.1**
**Milk, dairy products**	**318**	**73.4**	**448**	**78.2**	**767**	**76.1**
skimmed milk	96	22.0	320	55.9	416	41.3
milk with 1-1.5% fat content	101	23.3	60	10.5	161	16.0
yoghurt and Finnish cultured milk	44	10.1	13	2.2	57	5.6
cheese	45	10.3	32	5.6	77	7.6
milk-based desserts	7	1.7	11	1.8	18	1.8
**Sugar, sweets**	**6**	**1.4**	**2**	**0.3**	**8**	**0.8**
**Drinks**	**16**	**3.7**	**14**	**2.4**	**30**	**3.0**
**Miscellaneous**	**4**	**0.8**	**1**	**0.2**	**4**	**0.4**
**Total**	**434**	**100.0**	**573**	**100.0**	**1007**	**100.0**

Table S 50: Sources of calcium (mean and population proportion) in the diet on weekdays among Finnish 5- to 6-year-old preschool children who eat three meals at preschool. Main food groups and selected food groups are presented.

	**Outside preschool**		**Preschool**		**Total**	
	**mg/day**	**%**	**mg/day**	**%**	**mg/day**	**%**
**Vegetables, vegetable dishes**	**16**	**3.3**	**19**	**3.1**	**35**	**3.2**
**Potatoes, potato dishes**	**3**	**0.7**	**5**	**0.9**	**9**	**0.8**
**Fruit, berries, fruit and berry products**	**17**	**3.6**	**15**	**2.4**	**33**	**2.9**
**Cereals, bakery products**	**39**	**8.0**	**44**	**6.9**	**82**	**7.4**
**Fats, oils, gravy**	**2**	**0.4**	**2**	**0.3**	**4**	**0.4**
**Fish, fish dishes**	**6**	**1.3**	**9**	**1.5**	**16**	**1.4**
**Eggs, egg dishes**	**1**	**0.3**	**1**	**0.2**	**3**	**0.2**
**Meat, meat dishes**	**22**	**4.7**	**26**	**4.1**	**48**	**4.3**
**Milk, dairy products**	**350**	**72.7**	**498**	**78.8**	**848**	**76.2**
skimmed milk	122	25.3	343	54.4	465	41.8
milk with 1-1.5% fat content	81	16.8	75	11.9	156	14.0
yoghurt and Finnish cultured milk	52	10.9	12	1.8	64	5.7
cheese	48	9.9	36	5.7	84	7.5
milk-based desserts	11	2.2	13	2.1	24	2.2
**Sugar, sweets**	**10**	**2.0**	**2**	**0.3**	**12**	**1.0**
**Drinks**	**12**	**2.4**	**9**	**1.4**	**20**	**1.8**
**Miscellaneous**	**2**	**0.5**	**1**	**0.1**	**3**	**0.3**
**Total**	**481**	**100.0**	**632**	**100.0**	**1113**	**100.0**

Table S 51: Sources of magnesium (mean and population proportion) in the diet on weekdays among Finnish 3- to 4-year-old preschool children who eat three meals at preschool. Main food groups and selected food groups are presented.

	Outside preschool		Preschool		Total	
	mg/day	%	mg/day	%	mg/day	%
**Vegetables, vegetable dishes**	**8.5**	**8.3**	**8.2**	**5.6**	**16.8**	**6.7**
**Potatoes, potato dishes**	**4.2**	**4.0**	**8.4**	**5.7**	**12.5**	**5.0**
**Fruit, berries, fruit and berry products**	**11.0**	**10.7**	**7.7**	**5.2**	**18.7**	**7.5**
**Cereals, bakery products**	**27.2**	**26.5**	**55.9**	**37.9**	**83.1**	**33.2**
rye bread	4.5	4.4	7.3	5.0	11.8	4.7
rye crispbread	1.8	1.8	15.2	10.3	17.0	6.8
multi-grain bread	4.7	4.6	10.7	7.2	15.3	6.1
white bread	0.6	0.5	0.5	0.3	1.0	0.4
porridge	5.2	5.1	17.4	11.8	22.7	9.1
rice, pasta, etc.	3.9	3.8	1.9	1.3	5.9	2.3
**Fats, oils, gravy**	**0.2**	**0.2**	**0.4**	**0.3**	**0.6**	**0.3**
**Fish, fish dishes**	**2.3**	**2.2**	**5.3**	**3.6**	**7.6**	**3.0**
**Eggs, egg dishes**	**0.3**	**0.3**	**0.3**	**0.2**	**0.6**	**0.3**
**Meat, meat dishes**	**12.9**	**12.5**	**15.3**	**10.4**	**28.2**	**11.3**
**Milk, dairy products**	**28.5**	**27.7**	**42.7**	**28.9**	**71.2**	**28.4**
skimmed milk	9.5	9.2	31.8	21.6	41.3	16.5
milk with 1-1.5% fat content	9.3	9.0	5.5	3.7	14.8	5.9
**Sugar, sweets**	**1.8**	**1.8**	**0.6**	**0.4**	**2.5**	**1.0**
**Drinks**	**2.6**	**2.6**	**1.9**	**1.3**	**4.5**	**1.8**
**Miscellaneous**	**3.3**	**3.2**	**0.7**	**0.5**	**4.0**	**1.6**
**Total**	**102.9**	**100.0**	**147.4**	**100.0**	**250.3**	**100.0**

Table S 52: Sources of magnesium (mean and population proportion) in the diet on weekdays among Finnish 5- to 6-year-old preschool children who eat three meals at preschool. Main food groups and selected food groups are presented.

	Outside preschool		Preschool		Total	
	mg/day	%	mg/day	%	mg/day	%
**Vegetables, vegetable dishes**	**8.7**	**7.7**	**10.2**	**6.2**	**18.9**	**6.8**
**Potatoes, potato dishes**	**4.7**	**4.2**	**9.7**	**5.9**	**14.4**	**5.2**
**Fruit, berries, fruit and berry products**	**12.8**	**11.3**	**7.6**	**4.7**	**20.5**	**7.4**
**Cereals, bakery products**	**28.8**	**25.4**	**61.5**	**37.5**	**90.3**	**32.5**
rye bread	5.2	4.5	6.6	4.0	11.8	4.2
rye crispbread	1.3	1.1	16.9	10.3	18.2	6.5
multi-grain bread	5.7	5.0	11.1	6.7	16.8	6.0
white bread	0.8	0.7	0.9	0.5	1.6	0.6
porridge	5.4	4.8	19.5	11.9	24.9	9.0
rice, pasta, etc.	3.9	3.4	2.6	1.6	6.5	2.3
**Fats, oils, gravy**	**0.3**	**0.3**	**0.5**	**0.3**	**0.8**	**0.3**
**Fish, fish dishes**	**2.9**	**2.6**	**5.4**	**3.3**	**8.4**	**3.0**
**Eggs, egg dishes**	**0.3**	**0.3**	**0.2**	**0.1**	**0.6**	**0.2**
**Meat, meat dishes**	**14.6**	**12.8**	**19.1**	**11.6**	**33.7**	**12.1**
**Milk, dairy products**	**31.4**	**27.6**	**47.4**	**28.9**	**78.7**	**28.3**
skimmed milk	12.0	10.5	34.1	20.8	46.0	16.6
milk with 1-1.5% fat content	7.4	6.5	6.9	4.2	14.3	5.1
**Sugar, sweets**	**3.1**	**2.8**	**0.7**	**0.4**	**3.8**	**1.4**
**Drinks**	**1.7**	**1.5**	**1.0**	**0.6**	**2.7**	**1.0**
**Miscellaneous**	**4.1**	**3.6**	**0.9**	**0.6**	**5.0**	**1.8**
**Total**	**113.5**	**100.0**	**164.2**	**100.0**	**277.7**	**100.0**

Table S 53: Sources of iron (mean and population proportion) in the diet on weekdays among Finnish 3- to 4-year-old preschool children who eat three meals at preschool. Main food groups and selected food groups are presented.

	**Outside preschool**		**Preschool**		**Total**	
	**mg/day**	**%**	**mg/day**	**%**	**mg/day**	**%**
**Vegetables, vegetable dishes**	**0.25**	**7.7**	**0.27**	**6.6**	**0.51**	**7.1**
**Potatoes, potato dishes**	**0.12**	**3.7**	**0.25**	**6.1**	**0.37**	**5.0**
**Fruit, berries, fruit and berry products**	**0.22**	**6.8**	**0.16**	**3.9**	**0.38**	**5.2**
**Cereals, bakery products**	**1.16**	**36.4**	**2.19**	**54.0**	**3.36**	**46.2**
rye bread	0.18	5.6	0.28	7.0	0.46	6.4
rye crispbread	0.07	2.3	0.57	14.1	0.65	8.9
multi-grain bread	0.20	6.3	0.46	11.2	0.66	9.0
white bread	0.03	0.9	0.03	0.6	0.06	0.8
porridge	0.19	6.0	0.61	15.1	0.80	11.1
rice, pasta, etc.	0.15	4.7	0.08	1.9	0.23	3.1
**Fats, oils, gravy**	**0.01**	**0.4**	**0.03**	**0.7**	**0.04**	**0.6**
**Fish, fish dishes**	**0.08**	**2.4**	**0.15**	**3.6**	**0.22**	**3.1**
**Eggs, egg dishes**	**0.06**	**2.0**	**0.06**	**1.4**	**0.12**	**1.7**
**Meat, meat dishes**	**0.84**	**26.1**	**0.68**	**16.7**	**1.51**	**20.8**
cold cuts	0.02	0.7	0.02	0.4	0.04	0.5
red meat dishes	0.63	19.7	0.45	11.0	1.08	14.9
poultry dishes	0.11	3.5	0.13	3.2	0.24	3.3
sausage dishes	0.06	1.9	0.07	1.8	0.13	1.8
liver and blood foods	0.01	0.4	0.01	0.3	0.03	0.4
**Milk, dairy products**	**0.18**	**5.5**	**0.17**	**4.1**	**0.34**	**4.7**
**Sugar, sweets**	**0.07**	**2.0**	**0.01**	**0.3**	**0.08**	**1.1**
**Drinks**	**0.10**	**3.1**	**0.07**	**1.8**	**0.17**	**2.4**
**Miscellaneous**	**0.12**	**3.8**	**0.03**	**0.7**	**0.15**	**2.1**
**Total**	**3.20**	**100.0**	**4.06**	**100.0**	**7.26**	**100.0**

Table S 54: Sources of iron (mean and population proportion) in the diet on weekdays among Finnish 5- to 6-year-old preschool children who eat three meals at preschool. Main food groups and selected food groups are presented.

	Outside preschool		Preschool		Total	
	mg/day	%	mg/day	%	mg/day	%
**Vegetables, vegetable dishes**	**0.25**	**7.0**	**0.33**	**7.2**	**0.58**	**7.1**
**Potatoes, potato dishes**	**0.13**	**3.7**	**0.29**	**6.3**	**0.42**	**5.2**
**Fruit, berries, fruit and berry products**	**0.29**	**7.9**	**0.18**	**3.9**	**0.46**	**5.6**
**Cereals, bakery products**	**1.30**	**36.0**	**2.42**	**53.1**	**3.72**	**45.6**
rye bread	0.20	5.6	0.26	5.7	0.46	5.6
rye crispbread	0.05	1.4	0.64	14.0	0.69	8.4
multi-grain bread	0.25	6.9	0.48	10.4	0.73	8.9
white bread	0.04	1.1	0.05	1.0	0.09	1.1
porridge	0.19	5.2	0.69	15.0	0.87	10.7
rice, pasta, etc.	0.19	5.2	0.10	2.3	0.29	3.6
**Fats, oils, gravy**	**0.02**	**0.6**	**0.03**	**0.7**	**0.05**	**0.6**
**Fish, fish dishes**	**0.09**	**2.5**	**0.14**	**3.0**	**0.23**	**2.8**
**Eggs, egg dishes**	**0.06**	**1.7**	**0.05**	**1.0**	**0.11**	**1.3**
**Meat, meat dishes**	**0.97**	**26.8**	**0.85**	**18.6**	**1.82**	**22.2**
cold cuts	0.03	0.7	0.02	0.4	0.05	0.6
red meat dishes	0.70	19.5	0.55	12.1	1.26	15.4
poultry dishes	0.12	3.3	0.17	3.8	0.29	3.6
sausage dishes	0.05	1.3	0.06	1.3	0.11	1.3
liver and blood foods	0.07	2.0	0.05	1.0	0.12	1.5
**Milk, dairy products**	**0.19**	**5.3**	**0.19**	**4.1**	**0.38**	**4.6**
**Sugar, sweets**	**0.13**	**3.5**	**0.02**	**0.5**	**0.15**	**1.8**
**Drinks**	**0.06**	**1.7**	**0.04**	**0.8**	**0.10**	**1.2**
**Miscellaneous**	**0.12**	**3.4**	**0.04**	**0.8**	**0.16**	**1.9**
**Total**	**3.61**	**100.0**	**4.56**	**100.0**	**8.17**	**100.0**

Table S 55: Sources of zinc (mean and population proportion) in the diet on weekdays among Finnish 3- to 4-year-old preschool children who eat three meals at preschool. Main food groups and selected food groups are presented.

	**Outside preschool**		**Preschool**		**Total**	
	**mg/day**	**%**	**mg/day**	**%**	**mg/day**	**%**
**Vegetables, vegetable dishes**	**0.17**	**4.7**	**0.18**	**4.1**	**0.35**	**4.4**
**Potatoes, potato dishes**	**0.06**	**1.6**	**0.11**	**2.4**	**0.17**	**2.1**
**Fruit, berries, fruit and berry products**	**0.09**	**2.6**	**0.09**	**1.9**	**0.18**	**2.2**
**Cereals, bakery products**	**0.88**	**24.4**	**1.66**	**36.8**	**2.54**	**31.3**
rye bread	0.14	3.8	0.22	4.8	0.35	4.3
rye crispbread	0.05	1.4	0.42	9.4	0.48	5.9
multi-grain bread	0.15	4.1	0.33	7.3	0.48	5.9
white bread	0.02	0.6	0.02	0.4	0.04	0.5
porridge	0.17	4.7	0.51	11.2	0.68	8.3
rice, pasta, etc.	0.12	3.3	0.07	1.5	0.19	2.3
**Fats, oils, gravy**	**0.01**	**0.2**	**0.01**	**0.1**	**0.01**	**0.2**
**Fish, fish dishes**	**0.06**	**1.7**	**0.11**	**2.5**	**0.18**	**2.2**
**Eggs, egg dishes**	**0.04**	**1.0**	**0.03**	**0.7**	**0.07**	**0.9**
**Meat, meat dishes**	**1.02**	**28.3**	**0.89**	**19.7**	**1.91**	**23.5**
cold cuts	0.04	1.2	0.04	0.9	0.08	1.0
red meat dishes	0.76	21.0	0.57	12.6	1.33	16.4
poultry dishes	0.14	4.0	0.14	3.1	0.29	3.5
sausage dishes	0.07	1.9	0.13	2.9	0.20	2.4
**Milk, dairy products**	**1.13**	**31.4**	**1.38**	**30.6**	**2.51**	**31.0**
skimmed milk	0.32	8.8	1.06	23.5	1.38	16.9
milk with 1-1.5% fat content	0.33	9.1	0.02	0.4	0.35	4.3
**Sugar, sweets**	**0.03**	**0.8**	**0.00**	**0.0**	**0.03**	**0.4**
**Drinks**	**0.05**	**1.5**	**0.04**	**0.9**	**0.09**	**1.1**
**Miscellaneous**	**0.06**	**1.8**	**0.01**	**0.2**	**0.08**	**0.9**
**Total**	**3.61**	**100.0**	**4.51**	**100.0**	**8.12**	**100.0**

Table S 56: Sources of zinc (mean and population proportion) in the diet on weekdays among Finnish 5- to 6-year-old preschool children who eat three meals at preschool. Main food groups and selected food groups are presented.

	**Outside preschool**		**Preschool**		**Total**	
	**mg/day**	**%**	**mg/day**	**%**	**mg/day**	**%**
**Vegetables, vegetable dishes**	**0.19**	**4.6**	**0.23**	**4.6**	**0.42**	**4.6**
**Potatoes, potato dishes**	**0.06**	**1.6**	**0.13**	**2.5**	**0.19**	**2.1**
**Fruit, berries, fruit and berry products**	**0.11**	**2.7**	**0.09**	**1.7**	**0.20**	**2.2**
**Cereals, bakery products**	**0.97**	**23.9**	**1.82**	**36.2**	**2.79**	**30.7**
rye bread	0.16	3.9	0.20	3.9	0.36	3.9
rye crispbread	0.04	0.9	0.47	9.3	0.51	5.6
multi-grain bread	0.18	4.5	0.34	6.8	0.53	5.8
white bread	0.03	0.7	0.03	0.7	0.06	0.7
porridge	0.17	4.2	0.56	11.1	0.73	8.0
rice, pasta, etc.	0.13	3.3	0.08	1.6	0.21	2.4
**Fats, oils, gravy**	**0.02**	**0.4**	**0.01**	**0.1**	**0.02**	**0.2**
**Fish, fish dishes**	**0.08**	**1.9**	**0.12**	**2.3**	**0.20**	**2.1**
**Eggs, egg dishes**	**0.04**	**0.9**	**0.03**	**0.5**	**0.06**	**0.7**
**Meat, meat dishes**	**1.21**	**29.9**	**1.07**	**21.3**	**2.28**	**25.1**
cold cuts	0.05	1.2	0.05	1.0	0.10	1.1
red meat dishes	0.91	22.5	0.71	14.2	1.62	17.9
poultry dishes	0.16	3.8	0.18	3.6	0.34	3.7
sausage dishes	0.09	2.3	0.10	2.0	0.19	2.1
**Milk, dairy products**	**1.24**	**30.5**	**1.51**	**30.0**	**2.75**	**30.2**
skimmed milk	0.40	9.8	1.14	22.5	1.53	16.9
milk with 1-1.5% fat content	0.26	6.5	0.04	0.8	0.30	3.3
**Sugar, sweets**	**0.05**	**1.1**	**0.01**	**0.1**	**0.05**	**0.6**
**Drinks**	**0.03**	**0.7**	**0.02**	**0.3**	**0.05**	**0.5**
**Miscellaneous**	**0.07**	**1.8**	**0.02**	**0.3**	**0.09**	**1.0**
**Total**	**4.05**	**100.0**	**5.04**	**100.0**	**9.09**	**100.0**

Table S 57: Sources of iodine (mean and population proportion) in the diet on weekdays among Finnish 3- to 4-year-old preschool children who eat three meals at preschool. Main food groups and selected food groups are presented.

	Outside preschool		Preschool		Total	
	μg/day	%	μg/day	%	μg/day	%
**Vegetables, vegetable dishes**	**2.3**	**2.8**	**2.4**	**2.1**	**4.6**	**2.4**
**Potatoes, potato dishes**	**2.3**	**2.8**	**1.0**	**0.8**	**3.2**	**1.7**
**Fruit, berries, fruit and berry products**	**1.2**	**1.4**	**1.9**	**1.7**	**3.1**	**1.6**
**Cereals, bakery products**	**13.2**	**16.2**	**27.4**	**24.0**	**40.6**	**20.8**
porridge	4.5	5.5	20.9	18.4	25.4	13.0
**Fats, oils, gravy**	**0.8**	**1.0**	**0.6**	**0.6**	**1.4**	**0.7**
**Fish, fish dishes**	**5.8**	**7.1**	**12.3**	**10.8**	**18.2**	**9.3**
**Eggs, egg dishes**	**1.3**	**1.6**	**1.0**	**0.9**	**2.3**	**1.2**
**Meat, meat dishes**	**12.8**	**15.8**	**9.5**	**8.3**	**22.3**	**11.4**
**Milk, dairy products**	**38.9**	**47.8**	**56.7**	**49.8**	**95.6**	**49.0**
skimmed milk	12.7	15.5	42.4	37.2	55.0	28.2
milk with 1-1.5% fat content	13.5	16.5	8.0	7.0	21.5	11.0
**Sugar, sweets**	**0.7**	**0.8**	**0.1**	**0.0**	**0.7**	**0.4**
**Drinks**	**0.4**	**0.5**	**0.2**	**0.2**	**0.6**	**0.3**
**Miscellaneous**	**1.7**	**2.1**	**0.9**	**0.8**	**2.6**	**1.3**
**Total**	**81.4**	**100.0**	**113.9**	**100.0**	**195.3**	**100.0**

Table S 58: Sources of iodine (mean and population proportion) in the diet on weekdays among Finnish 5- to 6-year-old preschool children who eat three meals at preschool. Main food groups and selected food groups are presented.

	Outside preschool		Preschool		Total	
	μg/day	%	μg/day	%	μg/day	%
**Vegetables, vegetable dishes**	**2.6**	**2.8**	**3.0**	**2.3**	**5.5**	**2.5**
**Potatoes, potato dishes**	**2.6**	**2.8**	**1.2**	**1.0**	**3.8**	**1.7**
**Fruit, berries, fruit and berry products**	**1.5**	**1.6**	**1.8**	**1.4**	**3.3**	**1.5**
**Cereals, bakery products**	**15.3**	**16.6**	**31.8**	**24.8**	**47.1**	**21.4**
porridge	4.8	5.3	24.1	18.8	29.0	13.1
**Fats, oils, gravy**	**0.9**	**1.0**	**0.8**	**0.7**	**1.8**	**0.8**
**Fish, fish dishes**	**8.3**	**9.0**	**13.6**	**10.6**	**21.8**	**9.9**
**Eggs, egg dishes**	**1.2**	**1.3**	**0.8**	**0.6**	**2.1**	**0.9**
**Meat, meat dishes**	**14.1**	**15.3**	**11.3**	**8.8**	**25.4**	**11.5**
**Milk, dairy products**	**42.5**	**46.1**	**62.9**	**49.0**	**105.4**	**47.8**
skimmed milk	15.9	17.3	45.4	35.4	61.4	27.9
milk with 1-1.5% fat content	10.8	11.7	10.0	7.8	20.8	9.4
**Sugar, sweets**	**1.3**	**1.5**	**0.2**	**0.1**	**1.5**	**0.7**
**Drinks**	**0.3**	**0.4**	**0.1**	**0.1**	**0.5**	**0.2**
**Miscellaneous**	**1.4**	**1.5**	**0.8**	**0.6**	**2.2**	**1.0**
**Total**	**92.0**	**100.0**	**128.4**	**100.0**	**220.4**	**100.0**
